# Divergent evolutionary rates in vertebrate and mammalian specific conserved non-coding elements (CNEs) in echolocating mammals

**DOI:** 10.1186/s12862-014-0261-5

**Published:** 2014-12-19

**Authors:** Kalina TJ Davies, Georgia Tsagkogeorga, Stephen J Rossiter

**Affiliations:** School of Biological & Chemical Sciences, Queen Mary University of London, Mile End Road, London, E1 4NS UK

**Keywords:** Bats, Conserved non-coding elements, Ear development, Hearing/deafness

## Abstract

**Background:**

The majority of DNA contained within vertebrate genomes is non-coding, with a certain proportion of this thought to play regulatory roles during development. Conserved Non-coding Elements (CNEs) are an abundant group of putative regulatory sequences that are highly conserved across divergent groups and thus assumed to be under strong selective constraint. Many CNEs may contain regulatory factor binding sites, and their frequent spatial association with key developmental genes – such as those regulating sensory system development – suggests crucial roles in regulating gene expression and cellular patterning. Yet surprisingly little is known about the molecular evolution of CNEs across diverse mammalian taxa or their role in specific phenotypic adaptations. We examined 3,110 vertebrate-specific and ~82,000 mammalian-specific CNEs across 19 and 9 mammalian orders respectively, and tested for changes in the rate of evolution of CNEs located in the proximity of genes underlying the development or functioning of auditory systems. As we focused on CNEs putatively associated with genes underlying the development/functioning of auditory systems, we incorporated echolocating taxa in our dataset because of their highly specialised and derived auditory systems.

**Results:**

Phylogenetic reconstructions of concatenated CNEs broadly recovered accepted mammal relationships despite high levels of sequence conservation. We found that CNE substitution rates were highest in rodents and lowest in primates, consistent with previous findings. Comparisons of CNE substitution rates from several genomic regions containing genes linked to auditory system development and hearing revealed differences between echolocating and non-echolocating taxa. Wider taxonomic sampling of four CNEs associated with the homeobox genes *Hmx2* and *Hmx3 –* which are required for inner ear development *–* revealed family-wise variation across diverse bat species. Specifically within one family of echolocating bats that utilise frequency-modulated echolocation calls varying widely in frequency and intensity high levels of sequence divergence were found.

**Conclusions:**

Levels of selective constraint acting on CNEs differed both across genomic locations and taxa, with observed variation in substitution rates of CNEs among bat species. More work is needed to determine whether this variation can be linked to echolocation, and wider taxonomic sampling is necessary to fully document levels of conservation in CNEs across diverse taxa.

**Electronic supplementary material:**

The online version of this article (doi:10.1186/s12862-014-0261-5) contains supplementary material, which is available to authorized users.

## Background

Many Conserved Non-coding Elements (CNEs) are likely candidates for regulatory regions of gene expression [1]. Empirical evidence suggests that as little as a single nucleotide substitution in such a regulatory element is sufficient to significantly affect an organism’s morphology [2,3]. Moreover, taxon-specific sequence variation in CNEs may underpin particular phenotypic adaptations, such as increased forelimb length in bats [4] and craniofacial morphology in therian mammals [5], while in the three-spined stickleback (*Gasterosteus aculeatus*) the loss of a single CNE that is thought to be an enhancer of *Pitx1* is associated with pelvic reduction [6]. Conversely, however, the deletion of over 1,000 CNEs in mutant strains of mice was not seen to cause any detectable deleterious effects in terms of phenotype, gene expression and fitness [7]. A recent study quantified the presence or absence of 231,653 CNEs across seven diverse mammals, and found that many elements showed evidence of independent loss in several mammal lineages, although the phenotypic significance of this remains untested [8]. Recent evidence from Highland cattle (*Bos taurus*) and ‘fancy’ rats (*Rattus norvegicus*), have linked mutations in a conserved genomic region proximate to *Hmx1* to abnormal pinna development [9,10]. Interestingly, in rats the mutation involves a deletion of >5,000 base pairs whereas in cows there is a duplication of 76 base pairs. Although, the region affected by these mutations is many kilo-bases away from *Hmx1*, its altered expression is the likely cause behind the observed pinna phenotypes [9,10]. It therefore appears that the selective constraints acting on CNEs vary across different taxa, and that different genomic regions may all contribute differentially to the overall phenotype of an organism.

CNEs have been shown to either display high levels of conservation over wide evolutionary periods, or otherwise occur as group-specific elements (e.g. vertebrate, amniote and eutherian-specific) [11-13]. While some CNEs are known to act as *cis-*regulatory modules and are thus essential for the correct spatial and temporal expression of early developmental regulators [1], the precise functions of many CNEs remain unclear (see [14,15]). Numerous CNEs are located in clusters physically linked to genes governing development [1]. In cases where one or more CNE occur together with a single gene in a large genomic region, then a regulator role may potentially be assumed, however, where multiple genes exist it is not possible to unequivocally functionally assign a CNE to its most proximate gene [1]. Additionally, interactive databases have facilitated the identification of CNEs associated with the development of particular regions or by proximity to key developmental genes across evolutionary diverse taxa (e.g. [16,17]). The mode of action of some CNEs has been demonstrated empirically; for example, *in vivo* Zebrafish, *Danio rerio*, embryo assays can identify tissue-specific enhancer activity (e.g. [18]).

In this study we examined the molecular evolution of CNEs across mammalian lineages, with an emphasis on correlating differences in evolutionary rates in specific CNEs with divergent morphological features. In particular we examined the substitution rate of 3,110 vertebrate-specific and ~82,000 mammalian-specific CNEs, which are hypothesised to regulate the differential expression of genes at different stages during development and beyond. To test this hypothesis, we focused on the auditory system, and, in particular, echolocating bats and toothed whales, which have undergone structural modifications in their inner ears as well as neural adaptations to cope with the demands of processing the high frequency sounds produced during echolocation [19,20].

The evolutionary history and genetic control behind the development of the outer, middle and inner ears, which together form the mammalian auditory system, are well documented (e.g. [21,22]). The auditory system develops from the three germ layers and neural crest cells, with the mammalian cochlea being evident from ~10 days into embryonic development (for detailed review see [23]). Several genes implicated in the regulation of vertebrate ear development belong to the *PAX* and *SOX* gene families of tissue-specific transcription factors (TF), which are highly conserved across vertebrates (see [23-25]). Experimental evidence, mainly from gene knock-out mouse models, has shown that many of these genes are involved in controlling correct cochlear coiling and semicircular canal development (e.g. [24,26-28]). Many of these regulatory genes are under strong purifying selection with highly conserved amino acid sequences across divergent species (e.g. [29,30]). This suggests that apart from differences in coding sequences, other factors such as differences in expression levels may also explain the morphological diversity seen across vertebrates. Given that many CNEs are either known (or hypothesized) to regulate the expression of genes implicated in auditory system development, studying these elements may help to explain the observed morphological variation among mammalian auditory systems.

We predicted that CNEs located in the same genomic region as genes involved in auditory system development would show increased rates of substitution in echolocating bats and toothed whales compared to non-echolocating mammals that have less specialised auditory systems. In particular, we predicted that laryngeal echolocating bat species would show higher substitution rates in putative ‘ear development’ CNEs than those of non-echolocating Old World fruit bat species (Pteropodidae), in spite of the fact that echolocating bat taxa are not a true monophyletic group [31].

## Results

### Identification of CNEs

#### (A) Vertebrate-specific CNEs

In total 3,110 vertebrate-specific CNEs, defined as putative regulatory regions present in both mammals and fish, were downloaded from the COnserved Non-coDing Orthologous Regions (CONDOR) database [16], the *Homo* CNE orthologue sequences were then used to perform cross-species blastn searches (see Methods for parameters) in 45 mammalian species, including six bat genomes (see Additional file 1: Table S1). We calculated the percentage of missing data for each taxon, and excluded sequences from 19 species with >10% missing data, leaving a final alignment of 594,763 nucleotides from 26 species (Additional file 1: Table S1).

#### (B) Mammalian-specific CNEs

Our mammalian-specific CNEs are taken from Kim and Pritchard [32], and are characterised as being conserved across human, chimpanzee, dog, mouse and rat, but absent in chicken or fugu. Results of blastn searches using 82,064 of these mammalian-specific CNE queries across 19 mammalian genomes, gave broadly consistent results. In 17 of 19 species >70,000 CNEs were recovered (Additional file 1: Table S1c), while the number of significant hits was much lower (~50,000) in the hedgehog and shrew probably due to the low-coverage genomes.

### Phylogenetic analyses

#### (A) Vertebrate-specific CNEs

**Figure 1:**
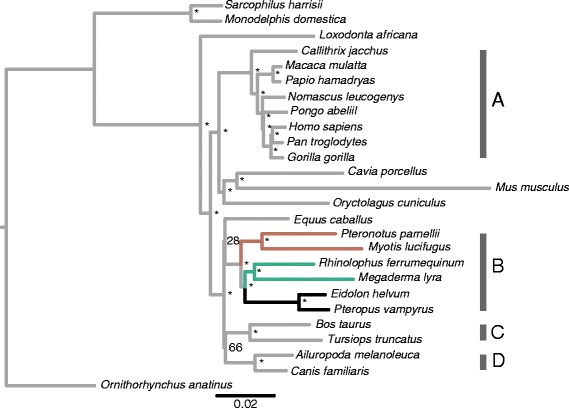
**Maximum likelihood phylogenetic tree based on concatenated CNEs (594,763 base-pairs) for 26 mammalian species with** ≥**90% sequence coverage of the 3,110 CNEs downloaded from CONDOR.** All nodes were recovered with 100% bootstrap support, unless otherwise shown. Bat branches are coloured as follows: Old World fruit bats (black); echolocating Yinpterochiroptera (green); Yangochiroptera (brown). Major clades are labelled as follows: Primates **(A)**; Chiroptera **(B)**; Cetartiodactyla **(C)** and Carnivora **(D)**.

A maximum likelihood (ML) tree summarising overall phylogenetic signal and lineage-specific nucleotide substitution rates of the concatenated alignment of 3,110 vertebrate CNEs for 26 species (Figure 1) correctly recovered the divisions between marsupials and placental mammals, and between the two superorders (Laurasiatheria and Euarchontoglires) (also see Additional file 1: Table S1). All nodes, including the bat sub-ordinal division of Yinpterochiroptera and Yangochiroptera, received maximum support with two notable exceptions that have also been questioned by recent studies (see Discussion): the node grouping Cetartiodactyla and Carnivora (66% bootstrap, BS), and the node uniting the horse with Chiroptera (28% BS). Examination of branch lengths revealed considerable differences within the Euarchontoglires; in particular between the Glires and Primates. The mouse *Mus musculus* was consistently characterised by the longest branch lengths, and thus had the greatest substitution rate, while apes and monkeys had relatively shorter branches and thus slower substitution rates. Within the superorder Laurasiatheria, all six bat species examined had similar branch lengths to the other five representative members.

#### (B) Mammalian-specific CNEs

Using the annotations provided by Kim and Pritchard [32], the 75,368 alignments that consisted of ≥15 taxa were concatenated into 9,806 alignment groupings each based on proximity to the same gene. Distances between each CNE and the nearest gene varied considerably, from being located in the intron to a distance greater than 100 kilo-bases, based on a reference human genome [32]. Of these concatenated sections, 6,109 alignments contained sequence information for all 20 taxa and were used to construct ML phylogenies. In order to visualise phylogenetic signal across wider chromosomal regions the resultant trees were grouped according to the 22 chromosomes of humans, (number of trees varied per chromosome considerably; Chromosome 19: 55 – Chromosome 1: 572), and were summarized using majority consensus trees (Additional file 2: Figure S1). Within Laurasiatheria, the bat, carnivore and cetartiodactyl clades were each consistently recovered as monophyletic. However, the position of each of these clades with respect to each other and to that of the horse, shrew and hedgehog varied considerably between regions. Within bats, the subdivisions of Yinpterochiroptera and Yangochiroptera were recovered by all consensus trees, apart from that for Chromosome 19, in which a polytomy was recovered. Within each subdivision, the correct familial placements were typically found; for example, within Yangochiroptera *Pteronotus parnellii* (Mormoopidae) was always found outside Vespertilionidae.

### Estimating substitution rates

#### (A) Vertebrate-specific CNEs

Results of Tajima’s Relative Rate Test [33] used to test for lineage-specific substitution rates (comparing each to the horse as a reference) revealed significant differences in the bats, cetartiodactyls and the two carnivores following corrections for the multiple corrections made (*P* < 0.05 in all cases; see Additional file 1: Table S2).

#### Vertebrate CNEs proximally located to ear development genes

**Figure 2:**
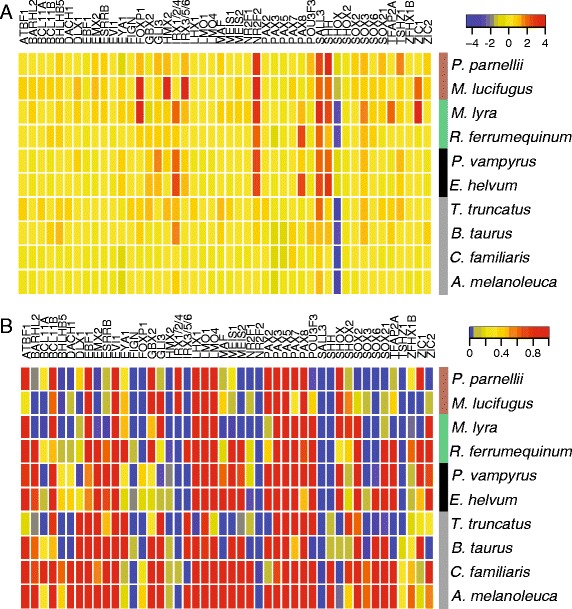
**Heat maps showing (A) the direction of change in substitution rates and (B) the significance level of this change, among lineages in 46 genomic regions.** In each comparison the rate is compared to that of the horse. Species and genomic regions are ordered according to similarity; this clustering is represented graphically by vertical and horizontal dendrograms respectively. Coloured bars indicate taxa: placental mammals (grey); Old World fruit bats (black); echolocating Yinpterochiroptera (green); Yangochiroptera (brown).

Tajima’s Relative Rates Test were applied to 46 alignments consisting of CNEs located in the same genomic region as genes with putative roles in ear development, and summarised with heat maps. Across species and gene regions there is considerable variation in terms of significant differences in substitution rate as well as either an increase or decrease in rates (Figure 2 and Table 1). All six bat species were found to have significantly greater substitution rates in CNEs from the *SHH* and *TSHZ1* genomic regions, compared to the horse. The bottlenose dolphin also had a significantly increased substitution rate in CNEs from the *TSHZ1* genomic region; this was not found in any of the other taxa (see Table 1). All four Yinpterochiroptera bats had significantly higher substitution rates in CNEs from the *IRX1/2/4* genomic region*.* All four species of laryngeal echolocating bats (i.e. excluding the Old World fruit bats) had significantly higher substitution rates compared to the horse for CNEs in the *FOXP1* and *HMX2/3* genomic region. The latter genomic regions also displayed significantly higher substitution rates in the bottlenose dolphin, but not the cow sequences. Therefore, CNEs from the *HMX2/*3 genomic region were found to have significantly higher substitution rates in all echolocating taxa but not in any of the non-echolocating taxa. Raw and *P*-values corrected for the multiple comparisons made across loci by applying both FDR and Holm correction are provided (Additional file 1: Table S3).

**Table 1:** **Summary of significant changes in relative rate found in a sample of 46 concatenated alignments of vertebrate-specific CNEs, grouped according to their location in the proximate genomic region of key developmental genes**

**Group**	**Sub-clade**	**Species**	**CNE alignment defined by genomic region:**
All species			*NR2F2* (H), *SALL3* (H)
	Carnivora		*FIGN* (H), *SHOX* (L)
		*A. melanoleuca*	*IRX1/2/4* (H)
		*C. familiaris*	*IRX3/5/6* (L)
	Cetartiodactyla		*BHLHB5* (H)*, DACH1* (H)*, FOXP1* (H)*, IRX1/2/4* (H)*, MAF* (H)*, SOX3* (H), *TFAP2A* (H)
		*B. taurus*	*NR2F1*(H)*, POU3F3* (H), *ZIC2* (H)
		*T. truncatus*	*BARHL2* (H)*, BCL11A* (H)*, FIGN* (H)*, HMX2/3* (H)*, LHX1* (H)*, MEIS1* (H)*, MEIS2* (H)*, PAX2* (H)*, SHOX* (L), *SOX6* (H), *SOX21* (H)*, TSHZ1*(H)
	Chiroptera		*SHH* (H), *TSHZ1* (H)
	Yinpterochiroptera		*IRX1/2/4* (H)
	Echolocating Yinpterochiroptera		*FOXP1* (H), *HMX2/3* (H), *SOX3* (H), *SOX6* (H)
		*M. lyra*	*BARHL2* (H)*, BCL11A* (H)*, BHLHB5* (H)*, DACH1* (H)*, EMX2* (H), *ESRRB* (H)*, FIGN* (H)*, IRX3/5/6* (H), *MAF* (H)*, MEIS1*(H), *MEIS2* (H)*, NR2F1* (H)*, TFAP2A* (H)*, ZFHX1B* (H)*, ZIC1* (H)
		*R. ferrumequinum*	*NA*
	Old World fruit bats		*FIGN* (H)*, IRX3/5/6* (H)
		*E. helvum*	*NR2F1* (H)
		*P. vampyrus*	*BCL11A* (H)*, DLX1* (H), *GLI3* (H), *SOX3* (H), *SOX6* (H)
	Yangochiroptera		*BARHL2* (H)*, BHLHB5* (H)*, EMX2* (H), *FIGN* (H), *FOXP1* (H), *HMX2/3* (H), *IRX3/5/6* (H), *MEIS2* (H)*, SOX6* (H)
		*P. parnellii*	*DACH1* (H)*, NR2F1* (H), *POU3F3* (H), *SOX2* (H), *SOX3* (H), *SOX21* (H), *ZIC2* (H)
		*M. lucifugus*	*BCL11A* (H)*, DLX1* (H)*, ZFHX1B* (H), *ZIC1* (H)

Significant differences in relative rates are defined as *P*-values less than 0.05 following Holm’s correction method for the multiple comparisons made. Higher rate in focal taxon – (H); lower rate in focal taxon – (L).

**Figure 3:**
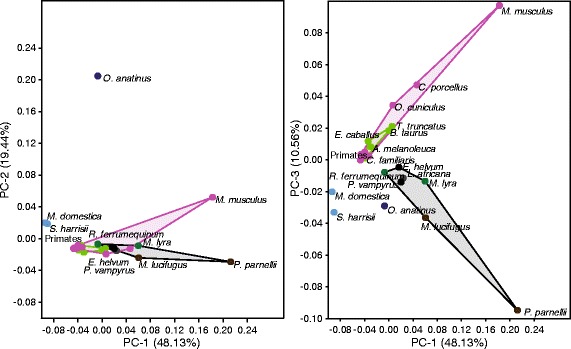
**PCA of CNE substitution rates across 26 mammal species.** Species positions are indicated with points: marsupials (light blue); monotremes (dark blue); Afrotheria (grey); Euarchontoglires (pink); non-bat Laurasiatheria (green); Old World fruit bats (black); echolocating Yinpterochiroptera (green); Yangochiroptera (brown). Convex hulls connect the following taxonomic groups: Chiroptera (black); Euarchontoglires (pink); non-bat Laurasiatheria (green).

Lineage-specific nucleotide substitution rate variation (summed branch lengths root-to-tip) for all 26 taxa and 83 CNE genomic regions (after excluding datasets with high proportions of missing data or any missing taxa), was visualised with a Principal Component Analysis (PCA). The first three principal components accounted for a total of 78.13% of the sample variance (PC1 – 48.13%; PC2 – 19.44%; PC3 – 10.56%). A PCA based on all CNEs for the 26 species (Figure 3) showed separation between placental mammals and marsupials along the first axis, and, within the placental mammals, some additional separation of *M. musculus* and *P. parnellii* from the other taxa. Loadings along PC1 were either positive or ~0; therefore, this axis corresponds to an overall substitution rate across CNEs (Additional file 3: Figure S2A). Principal component 2 separated the two Yangochiroptera from the remaining placental mammal species. The lowest loadings along PC2, and so corresponding to the placement of the Yangochiroptera, included CNEs from the *SHH, NR2F2* and *MAB21L1* genomic regions; whereas the highest loadings corresponded to the *EYA1* and *AUTS2* genomic regions (Additional file 3: Figure S2B). The final principal component examined, PC3, separated all bats with the African elephant from the other placental mammals. Moreover, within bats, PC3 separated Yangochiroptera from Yinpterochiroptera. The low loadings along this axis include CNEs from the *MAB21L1*, *SHH*, *EBF3* and *NR2F2* genomic regions; and the highest include CNEs from the *MAF* and *PAX7* genomic regions (Additional file 3: Figure S2C)*.*

Branch-specific substitution rates plotted for all tip and ancestral bat and Cetartiodactyla branches for the 83 CNE genomic regions (Additional file 4: Figure S3) corroborated the high degree of variation seen in substitution rates across species and genomic regions. Within bats, typically the ancestral branches (i.e. common bat, common Yinpterochiroptera, common echolocating Yinpterochiroptera and common Yangochiroptera) had lower substitution rates than the tips, although the ancestral Old World fruit bat branch had a higher substitution rate than the two Old World fruit bat tips. Within Cetartiodactyla, the echolocating dolphin and cow generally had consistent substitution rates which were typically higher than the ancestral branch.

#### Analysis of vertebrate CNE conservation or accelerated evolution

We conducted likelihood ratio tests to identify specific sites that were subject to either statistically evolutionary conservation or acceleration in the focal clades, and found evidence of strong conservation overall. Across all 26 species considered, at least 40% of sites showed high levels of conservation (i.e. a positive conservation score) and ~5% showed evidence of being under significant accelerated evolution (i.e. those identified with a negative acceleration score and negative *P*-value <0.05) (Additional file 5: Figure S4). Furthermore, across the different bat clades examined levels of conservation and acceleration were similar across most genomic regions.

#### (B) Mammalian-specific CNEs

Frequency distributions of relative rates across mammalian CNEs in the focal taxa in the two groups, bats and cetaceans, show that within each taxonomic group rates are broadly consistent (Additional file 6: Figure S5). To summarise, out of 5,726 genomic regions containing CNEs, Old World fruit bats had 2,320 regions with a relative rate of <0.90 and 1,549 regions with a relative rate of >1.10. The same rates were seen in 1,624 and 2,225 CNE regions, respectively in echolocating Yinpterochiroptera (out of 5,724) and 948 and 3,144 CNE regions, respectively in Yangochiroptera (out of 5,725). The median rate and standard deviation for Old World fruit bats, echolocating Yinpterochiroptera and Yangochiroptera were 0.955 ± 0.34 SD; 1.031 ± 0.31 SD; 1.131 ± 0.38 SD respectively. Each cetacean dataset consisted of 6,437 genomic regions; the non-echolocating minke whale dataset contained 4,431 CNE regions with a relative rate >0.90 and 908 CNE regions with a relative rate >1.10. This compares with the dolphin CNE dataset which contained 3,290 and 1,542 CNE regions in the same rate categories. The minke whale median rate was 0.781 ± 4.98 SD, and the dolphin median relative rate was 0.891 ± 4.61 SD.

#### Mammalian CNEs proximally located to hearing and ear development genes

**Figure 4:**
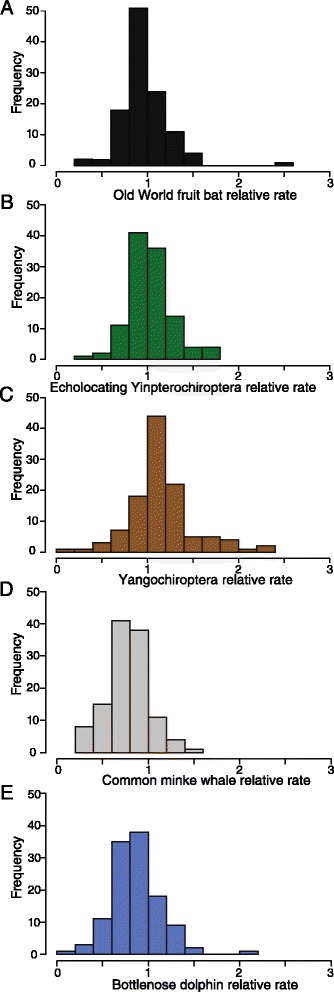
**Frequency histograms of estimated relative rates of CNEs located in the same genomic regions as putative ‘hearing/deafness’ genes – in each analysis the rate for the foreground clade of interest in given relative to the background rate, which is equal to 1.** Rates were calculated in BASEML using the Felsenstein-84 model of substitution, local clock and alpha and kappa estimated from the data **(A)** Old World fruit bats; **(B)** echolocating Yinpterochiroptera; **(C)** Yangochiroptera; **(D)** Common minke whale and **(E)** Bottlenose dolphin.

**Figure 5:**
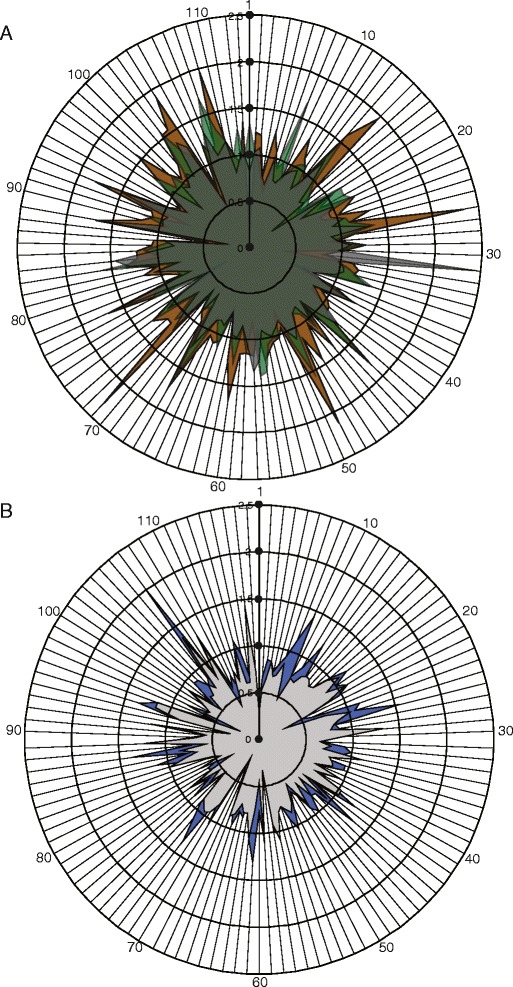
**RADAR plots of the estimated relative rates of CNEs located in the same genomic regions as putative ‘hearing/deafness’ genes for (A) bats and (B) cetaceans.** Numbers refer to each of the putatively associated genes (see Additional file 1: Table S5). Colours correspond to the following groups and species: Old world fruit bats (dark grey); echolocating Yinpterchiroptera (green); Yangochiroptera (brown); Common minke whale (blue) and Bottlenose dolphin (light grey).

In total CNE alignments from 113 and 118 genomic regions located proximally to putative hearing/deafness and auditory system development genes were compared across bats and cetaceans respectively (see Figures 4 and 5). The distribution of relative rates for this subset of CNEs broadly matched that of the whole sample (Additional file 6: Figure S5). Within bats, several CNEs from Yangochiroptera appeared to have a faster relative rate of evolution compared to Old World fruit bats and echolocating Yinpterochiroptera (see Figure 5). However, the median rate and standard deviation across this sample of CNEs from Old World fruit bats, echolocating Yinpterochiroptera and Yangochiroptera are 0.950 ± 0.26 SD; 1.008 ± 0.26 SD; 1.120 ± 0.36 SD respectively, and thus are similar. For cetaceans, the median rate and standard deviation were 0.775 ± 0.23 SD for the non-echolocating minke whale, and 0.846 ± 0.28 SD for the echolocating dolphin.

### Study of CNEs in the Hmx2/3 gene region of echolocating taxa

Given the observed variation in the substitution rate of the *Hmx2/3* gene region across echolocating bats and the bottlenose dolphin, we looked more closely at sequence variation in CNEs located in this genomic region in a wider range of bat and cetacean species for which published genomes are available with a variety of echolocation call types. Sequences for 24 CNEs from this genomic region were aligned for a maximum of 10 echolocating species and 25 non-echolocating species and branch lengths estimated (see Additional file 7: Figure S6). Across these elements a pattern emerged with bat species from the Vespertilionidae family (suborder Yangochiroptera) typically displaying the longest branch lengths. Within the Yinpterochiroptera, in the majority of the elements the non-echolocating Old World fruit bats had shorter branch lengths compared to the remaining echolocating species. In many of these elements branch lengths were also higher in the echolocating cetaceans compared to the non-echolocating baleen whale; however, this was not consistent across all elements. The number of significantly accelerated sites, as identified by phyloP, across the 24 regions in three focal clades of bats, echolocating Yinpterochiroptera, Old World fruit bats and Yangochiroptera, revealed a similar pattern to above (see Additional file 8: Figure S7A). With the echolocating clades typically having a higher number of accelerated sites compared to the Old World fruit bats. In the case of Yangochiroptera this is particularly evident in elements CRCNE00009713, CRCNE00009716 and CRCNE00009717 which have 3.35, 2.29 and 1.68% of sites identified as significantly accelerated, these elements are some of the most proximally located to *HMX2* and *HMX3* based on the human annotation (see Additional file 8: Figure S7C). In the clade of toothed whales, again element CRCNE00009713 has the highest number of significantly accelerated sites, which corresponds to 2.87% of the total element (see Additional file 8: Figure S7B).

**Figure 6:**
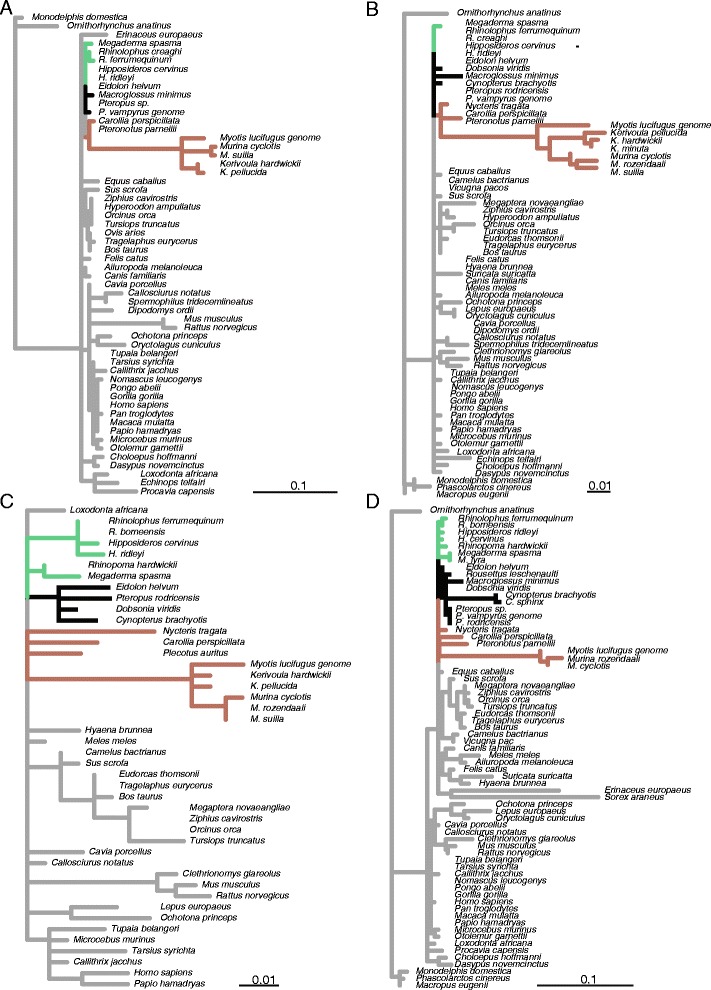
**Estimated lineage-specific nucleotide substitution rates across four CNE sequence alignments from the** ***Hmx2/3*** **gene region.** Rates were calculated in BASEML using the Felsenstein-84 model of substitution, no clock and alpha and kappa estimated from the data **(A)** CRCNE00009707; **(B)** CRCNE00009711; **(C)** CRCNE00009716; **(D)** CRCNE00009741. Non-bat branches (grey) and bat branches are coloured as follows: Old World fruit bats (black); echolocating Yinpterochiroptera (green); Yangochiroptera (brown). See Additional file 7: Figure S6 for more information regarding the approximate genomic location (in kilo-bases) of each of the four CNEs under study and the three proximate genes (*HMX3*, *HMX2* and *BUB3*) based on the human genome.

Four of the above *Hmx2/3* CNEs were further sequenced in nine bat families: Rhinolophidae, Hipposideridae, Megadermatidae, Rhinopomatidae, Pteropodidae, Phyllostomidae, Mormoopidae, Vespertilionidae and Nycteridae; and three cetacean families: Balaenopteridae, Ziphiidae and Delphinidae. Estimated branch lengths for trees based on all four CNEs showed broadly similar patterns (Figure 6). For these four CNEs, particular members of the bat family Vespertilionidae were seen to have the longest branch lengths of all mammals included, and thus were even longer than those of rodents. Interestingly, however, these high substitution rates were not seen among all bat species; particularly *Myotis lucifugus*, *Kerivoula* spp. and *Murina* spp. displayed long branches, whereas *Plecotus auritus* did not for the CRCNE00009716 element (Figure 6C). For all four CNE datasets, all Yinpterochiroptera species showed similarly low substitution rates. Genomic alignments of human, mouse, rat and fugu suggested that, of the four CNEs amplified, CRCNE00009716 is the most proximally located to *Hmx2* and *Hmx3*. This was confirmed by examination of the recently completed *Pteropus alecto* genome [34]; this suggests ~4 kb separates the 3′ end of CRCNE00009716 and the start codon of *Hmx2*. In CRCNE00009741, which is most distally located to either *Hmx2* or *Hmx3*, the Old World fruit bats *Cynopterus brachyotis* and *C. sphinx* had considerably longer branch lengths than the remaining Yinpterochiroptera species. Within this sample, again there was no consistent difference in branch lengths between the non-echolocating humpback whale, *Megaptera novaeangliae*, and the remaining echolocating toothed whales.

## Discussion

Previous studies attempting to link genetic variation and phenotypic adaptations in echolocating mammals have tended to focus on selection pressures acting on protein coding genes (e.g. [35,36]), thus often placing less emphasis on the molecular evolution of putative regulatory regions or transcription factors. However, given their role in the regulation of gene expression these two genomic components will undoubtedly play a role in determining species-specific adaptations. Here we attempted to relate auditory adaptations of echolocating taxa to changes in the molecular evolution of putative regulatory regions located proximally to mammalian genes associated with the auditory system. The development and maintenance of the mammalian auditory system is a highly complex process requiring the controlled expression of a high number of genes along both a temporal and spatial manner (for review [23]). Whereas genes responsible for the basic mammalian auditory plan have been well characterised (for example, see [37]), those responsible for species-specific adaptations are less known. CNEs conserved across all vertebrates are likely to be responsible for the early stages of embryonic development; we therefore also screened echolocating taxa for CNEs conserved only across mammals.

### Phylogenetic analysis of CNEs

Despite their high levels of conservation, ML phylogenies constructed from concatenated CNEs recovered the major sub-divisions within mammals, and, in many cases, the correct familial placements. Thus lineage-specific substitutions have accumulated in sufficient numbers to provide phylogenetic signal. In all cases bats were recovered as monophyletic, and support was found for the subordinal division of Yinpterochiroptera and Yangochiroptera. Although the horse (*Equus caballus*) was recovered as the sister taxon of bats by a concatenated analysis of vertebrate specific CNEs, this grouping corresponding to Pegasoferae [38] received low support. The Pegasoferae topology remains controversial and conflicts with the species tree topology typically recovered by phylogenetic analysis of coding sequences (e.g. [39]). Furthermore this grouping was not supported by analyses of mammalian specific CNEs.

### CNEs proximally located to auditory system genes

Lineage specific substitution rates calculated for focal genomic regions revealed several trends within bats and between bats and other mammals. We found evidence for significantly greater substitution rates in all six bat species compared to the horse in vertebrate specific CNEs from two genomic regions (*SHH* and *TSHZ1*). The *SHH* gene has a known association with inner ear development (e.g. [40]), but is also associated with the development of limbs and lungs (e.g. [41,42]) that are noteworthy for showing modifications in bats. In the case of *TSHZ1*, the bottlenose dolphin was seen to share an increased substitution rate, which could relate to this gene’s biological association with middle ear morphogenesis [43]. Anatomically, mammalian middle ear ossicles are frequently cited as one of the important auditory system components for high-frequency hearing [44] and it is thus noteworthy that cetaceans possess specialised middle ears (e.g. [45]).

Despite these patterns, overall we found limited support for the prediction that vertebrate specific CNEs located in the same genomic region as genes involved in auditory system development show increased rates of substitution in echolocating taxa. In particular, a PCA summarizing substitution rates did not clearly separate echolocating taxa from other taxa. However, within bats, a distinction between the bat suborders was apparent, mainly driven by rate changes in genomic regions such as *SHH*, *NR2F2* and *MAB21LI*. In addition, along PC1 and PC3, *Pteronotus parnellii* was found to be more distantly located compared to the other bat species. This is interesting given that *P. parnellii* is unique among Yangochiroptera bats as having evolved high-duty constant-frequency echolocation [46]. Levels of conservation and accelerated evolution of genomic regions were approximately equal when clades of echolocating Yinpterochiroptera, non-echolocating Yinpterochiroptera and Yangochiroptera are considered. Overall, therefore, while differences in some ear development CNEs substitution rates were seen between Old World fruit bats and laryngeal echolocating bats, many differences were also found between echolocating bat species from the same sub-clades, and even some between the two non-echolocating bat species. Such variation precludes any firm conclusions but could reflect the involvement of different genes in bats that have evolved divergent modes of echolocation.

Among the bats, substitution rates were found to be lowest along both ancestral branches of all extant bats and all Yinpterochiroptera. On the other hand among tips, rate comparisons across species revealed several differences; for example, the two Yangochiroptera tended to have higher substitution rates compared to Yinpterochiroptera. Several previous studies have related the molecular evolution of protein-coding ‘hearing’ genes to the acquisition of echolocation in bats and cetaceans (e.g. [47-49]). Of these, documented cases of sequence convergence between divergent echolocating taxa have been suggested to support multiple origins of echolocation within bats (e.g. [47]). Our own findings that putative ear development CNEs shows no increased substitution rates in the common bat ancestral branch yet do show divergent rates within laryngeal echolocating bat branches would also appear to be consistent with the later acquisition of echolocation in extant bat lineages.

Numerous genes have been implicated in mammalian hearing, and also through the series of recent studies, mentioned above, several candidate ‘echolocation’ genes have been proposed (for examples see [47,48]). Recently transcriptomes have been used to quantify expression differences in the cochleae of echolocating and non-echolocating bat species [50]. Interestingly *TMC1* was identified as the gene showing the highest significant up-regulation in the echolocating *Myotis ricketti* compared to the non-echolocating Old world fruit bat, and this gene was also previously shown to have several parallel amino acids between echolocating taxa [47]. Several mammalian specific CNEs putatively located either within the introns or within 100 kb of this gene were included in our study, and relative rates tests conducted with PAML found that both echolocating sub-clades of bats had relative rates >1.00 compared to the Old World fruit bat in which the relative rate was <1.00 (see Figure 5). However, a similar pattern was not seen in the mammalian-specific CNEs putatively located close to *SIX1* and *MYO3A*, which were also previously shown to be up-regulated in echolocating bats [50], but which showed approximately equal relative rates in all three bat clades, or lower in echolocating clades.

Focusing on echolocating taxa specifically, the four laryngeal echolocating bat species and the bottlenose dolphin initially studied had significantly greater substitution rates, compared to the horse, in CNEs from the *HMX2* and *HMX3* genomic regions. This was confirmed by the addition of additional echolocating taxa. Wider family level bat comparisons revealed that four conserved elements located in the *HMX2/3* genomic region typically had the greatest substitution rates in the Vespertilionidae. Substitution rates were particularly high in species with the highest frequency echolocation calls that also have a high repetition rate (e.g. *Kerivoula* spp. and *Murina* spp. [51]); whereas, no increase was seen in *P. auritus* known as a ‘whispering’ bat due to its low-intensity calls [52]. The substitutions evident in the Vespertilionidae CNEs were typically clustered at one end – raising the possibility that CNE may be truncated in these species.

The CNEs examined in this region span across ~500 kilo-bases in the human genome, within this region based on Ensembl annotation there are three genes – *BUB3, HMX2* and *HMX3*. The Bub3 protein is encoded by *BUB3* which is an essential component of the mitotic spindle assembly complex during early development, disruptions to this gene prove lethal [53]. The two homeobox genes, *Hmx2* and *Hmx3*, are associated with nervous system development and inner ear morphogenesis, particularly the vestibular portion ([54] and for review see [37,55]). Expression of *Hmx3* begins eight hours earlier than *Hmx2* in the otic epithelium of developing mammalian ears, otherwise both are co-expressed [56]. Mutational studies of mice suggest each gene plays distinct roles in terms of morphogenesis and cell specification during development. Null *Hmx2* mutants develop abnormal vestibular apparatus; with inner ears entirely lacking semicircular canals, with fused utricles and saccules and a significant loss of vestibular sensory epithelium [54]. Furthermore, null *Hmx2* mice display abnormal behaviours such as circling and hyperactivity. However, effects of *Hmx3* null mice mutants appear to be strain specific. In some strains the gross morphology of the inner ear is not affected (though a significant loss of sensory cells in the vestibular portion and behavioural abnormalities were reported) [56]. Whereas, in a different strain, null *Hmx3* mutation resulted in hyperactivity and circling behaviour as well as failure of semicircular canal development [24]. Double null mutant (*Hmx2/3*) mice have severe anatomical and neurosensory defects and entirely lack vestibular systems [55].

In *Hmx2*, *Hmx3* and *Hmx2/3* null mice mutants cochlea gross structure appears unaffected, hearing capabilities in *Hmx3* mutants appear normal but have not been directly tested in double mutants [24,55]. However, a recent study proposed that hemizygous loss of the *HMX2/3* genome region may have led to congenital sensorineural hearing loss in two un-related humans [57]. There have been no reported defects in the central nervous system in single *Hmx2* or *Hmx3* mutants, suggesting a redundant role [55]. If the detected differences in substitution rate in the bat CNEs from the *HMX2/3* genomic region alter the expression of these genes during auditory system development then it is possible the gross morphology of the inner ear may be affected, though this remains untested. This is particularly interesting given the highly variable nature of bat inner ears, which include modifications to their semicircular canals (e.g. [20,58]).

On average the Yangochiroptera – the suborder containing Vespertilionidae – appeared to have higher substitution rates compared to the Yinpterochiroptera across many CNEs. Within Yangochiroptera, Vespertilionidae have previously been shown to have a significantly faster substitution rate in a sample of mitochondrial genes and the nuclear gene *RAG2* compared to Phyllostomidae [59]. The previous evidence for differences in substitution rates coupled with their rapid diversification suggests that unique selective constraints may be acting on the genomes of Vespertilionidae.

Previously documented causes for differences in lineage-specific nucleotide substitution rates are well recognised (as reviewed in [60,61]) and include effective population size (N_e_) and generation time, as well as the extent to which changes are adaptive [62,63]. Thus these parameters may have contributed to the results of the relative rates tests performed in this study. Accurate generation times for many bat species are unknown, published values range from 2–8.34 years [64,65], thus longer than those of most rodents and more comparable to larger bodied laurasiatherians (e.g. horse – ~8 years [66]; cow – ~5 years [67]; dolphin – ~20 years [68]). The quality of comparative genome data is also an important factor; only two (horse and dog) out of the Laurasiatheria species have high coverage (excluding the two bats and bottlenose dolphin), both of which are arguably not optimal due to domestication. Unfortunately genomes for species with body sizes and population sizes more similar to those of bats, such as the common shrew or hedgehog, were only available at low-coverage. As reference points, the non-focal taxa studied, such as carnivores and artiodactyls, were also shown to have variable substitution rates in several genomic regions. Therefore, it seems that lineage-specific rates of substitution in CNEs in vertebrates occur relatively frequently. Indeed fishes have also been previously shown to display lineage-specific differences in CNE rates of substitution [69,70].

The CNEs that we identify as showing elevated substitution rates in echolocating mammals represent valuable candidates for future study. Ultimately, attempts to link patterns of our focal CNE substitution rates to genes underpinning ear development will require functional assays to better characterize their roles in gene regulation.

## Conclusions

In this first attempt to relate levels of selective constraint acting on CNEs, located in the same genomic regions as genes associated with the auditory system, of echolocating mammals we found variation in substitution rate in each of the two bat suborders. Although, the functional significance of variation in substitution rates and CNE sequences found in the bat sequences currently remains unknown, this could suggest that different selective constraints have acted on the developmental pathways of the auditory systems in each of the two bat suborders. The 3,110 vertebrate and ~82,000 mammalian CNEs examined here represent a fraction of the total number of CNEs contained within a typical mammalian genome [32,71]. Therefore, although this study may have detected some of the large scale evolutionary signals contained within the non-coding component of bat genomes many more fine-scale patterns may remain.

## Methods

### Identification of CNEs

#### (A) Vertebrate-specific CNEs

We downloaded 3,110 target vertebrate-specific CNEs, defined as putative regulatory regions present in both mammals and fish, for human (*Homo sapiens*), mouse (*Mus musculus*), rat (*Rattus norvegicus*), dog (*Canis familiaris*) and fugu (*Takifugu rubripes*) from the CONDOR database [16] that met the criteria of being conserved with ≥60% identity, and minimum sequence length of 100 nucleotides (final species coverage: human: 3,110, fugu: 3,086, mouse: 3,047, rat: 2,599, dog: 439). The 3,110 *Homo* sequences were used to perform cross-species blastn searches in 45 mammalian species, including six publically available bat genomes [39,72] (see Additional file 1: Table S1). From the bat suborder Yinpterochiroptera we obtained CNE data from the non-echolocating Old World fruit bats *Eidolon helvum* and *Pteropus vampyrus* (Pteropodidae)*,* the echolocating greater horseshoe bat *Rhinolophus ferrumequinum* (Rhinolophidae) and the echolocating greater false vampire bat *Megaderma lyra* (Megadermatidae). From the suborder Yangochiroptera we obtained data from the echolocating taxon Parnell's moustached bat *Pteronotus parnellii* (Mormoopidae) and the little brown bat *Myotis lucifugus* (Vespertilionidae). In each blastn search the top hit was retained, with minimum expected (e) value thresholds of 10^−6^ and a minimum of 60% sequence identity, and, in cases when the top hit sequences were not continuous along the subject sequence, only the longest portion was retained. We calculated the percentage of missing data for each taxon, and excluded sequences with >10% missing data.

#### (B) Mammalian-specific CNEs

To investigate the evolution of mammalian-specific CNEs in echolocating taxa we used the dataset originally identified by Kim and Pritchard [32] as a starting point. We downloaded human sequences for 82,335 CNEs (hg17) from the UCSC Genome Browser [http://genome.ucsc.edu/]; these sequences represent CNEs that were found to be conserved across human, chimpanzee, dog, mouse and rat, but absent in chicken or fugu [32]. We confirmed that these represented non-coding regions of the genome by blastx using the human peptide database downloaded from Ensembl (release 73). Sequences with significant hits (e <10^−3^) were excluded from further study; this resulted in 271 CNEs being discarded. The remaining 82,064 sequences were used as queries in cross-species blastn searches of 19 mammalian genomes (Additional file 1: Table S1). Echolocating species were represented by the following bats: *R. ferrumequinum*, *M. lyra, P. parnellii, M. lucifugus, Myotis davidii* [34] and *Eptesicus fuscus* and the bottlenose dolphin *Tursiops truncatus.* For comparison we included the non-echolocating Old World fruit bats *P. vampyrus, Pteropus alecto* [34] and *E. helvum*; and the baleen whale *Balaenoptera acutorostrata* [73]. The same parameters were used as before, sequence identity ≥60% and the longest sequence kept. Alignments were constructed using default settings in MAFFT.

### Phylogenetic analyses of CNEs

#### (A) Vertebrate-specific CNEs

To investigate overall phylogenetic signal and lineage specific nucleotide substitution rates a phylogenetic tree was constructed based on a concatenated alignment of all CNEs for 26 species. A maximum likelihood (ML) phylogeny was constructed with RAxML v.7.2.8 [74] under the GTR + Γ + I substitution model with 1,000 bootstraps. Prior to analysis sites consisting of entirely missing data were removed leaving a final alignment of length 594,442 bp.

#### (B) Mammalian-specific CNEs

From the initial alignments, the 6,661 sequence alignments consisting of fewer than 15 taxa were discarded; leaving 75,368 alignments. Individual CNEs were then concatenated according to the nearest gene, in either direction, based on the annotation of a reference human genome provided by Kim and Pritchard [32], distances between CNEs and the nearest gene varied considerably, from being located in the intron to a distance greater than 100 kilo-bases. This resulted in concatenated alignments corresponding to 9,806 genomic regions (6,109 alignments – 20 taxa; 1,777 – 19 taxa; 950 – 18 taxa; 453 – 17 taxa; 304 – 16 taxa and 213 – 15 taxa). Maximum likelihood (ML) phylogenies were constructed with RAxML v.7.2.8 [74] under the GTR + Γ substitution model for the 6,109 concatenated alignments with all 20 species, with human, mouse and guinea pig set as out-groups. Trees were grouped according to the 22 chromosomes of humans, and majority consensus trees constructed in Dendroscope [75].

### Substitution rate in CNEs

#### (A)Vertebrate-specific CNEs

To further explore lineage-specific variation in substitution rates we performed Tajima’s Relative Rates Test [33], in MEGA5 [76], on the concatenated 3,110 CNE alignment. In each comparison, one of the six bat species, the bottlenose dolphin, cow, dog and panda was compared in turn to the horse. The horse was chosen as the reference sequence due to the good quality genome and the comparable generation time with bats. Published simulations suggest choice of outgroup should not effect the results of relative rates tests significantly providing it is valid and minimizes the evolutionary distance to the ingroup [77]. Within Euarchontoglires, we discounted rodents due to their documented high substitution rates [32], and instead chose the human due to the high coverage genome. To correct for the ten tests we adjusted the calculated *P*-values using both the false discovery rate (FDR) [78] and Holm’s method [79].

### Vertebrate CNEs proximally located to ear development genes

The CONDOR database holds information on CNEs initially identified with sensitive multiple alignments of orthologous genomic regions using *Fugu*, as a baseline, with mammalian genomes. Furthermore, these CNE clusters and their associated transcriptional-regulation and/or development genes have been shown to be conserved in synteny across these species [1,16]. Each of our subset of CNEs downloaded from CONDOR has therefore been assigned to one of 89 reference genomic regions, with each region having been named arbitrarily after the *trans-dev* genes from that region [16]. It is acknowledged that these genomic regions can span several mega-bases, thus CNEs may be located a considerable distance from the *trans-dev* gene in question. However, the fact that the CNEs have remained in a conserved syntenic order with the *trans-dev* gene across mammal and fish genomes suggests a possible functional association. In many genomic regions, e.g. *DACH1*, there is one annotated gene. Alternatively, several genes may be present in a region, e.g. *HOXD –* in which CNEs have been shown to control the expression of multiple genes simultaneously [80]. Although this annotation does not always imply a direct functional association between CNEs and gene, for our study we took the conserved spatial association as potential evidence of this. Cross-referencing these nominal genes with published empirical studies of gene expression and gene knock-out mutants suggested 49 genes (corresponding to 46 of the possible 89 genomic regions) were likely to have roles in auditory system development (Additional file 1: Table S4). The CONDOR database suggested the following genes are located in the same genomic region: *Nkx-5.1* (*HMX2*) with *Nkx-5.2* (*HMX3*), *IRX1* with *IRX2* and *DLX1* with *DLX2*. Henceforth CNEs located in the same genomic region as these genes will be referred to as ‘putative ear development CNEs’ for the purpose of this study (N.B. this does not imply a proven function). For each of the 89 genomic regions concatenated alignments of their respective CNEs were constructed based on the 26 mammalian genomes and each alignment is named after the reference gene. These alignments have been deposited in the Dryad Repository: http://dx.doi.org/10.5061/dryad.50kd5.

We used two approaches to quantify substitution rates of putative ear development CNEs. First, for the 46 genomic concatenated regions (containing the 49 ear development genes) Tajima’s Relative Rates Test was used to explore the substitution rates using the methods described above. Corrections for multiple comparisons were made using the FDR and Holm’s method. Second, lineage-specific nucleotide substitution rates were estimated for all gene region CNEs for the 26 species using BASEML in the PAML4.4 package [81]. For these analyses we set the tree topology to the currently accepted phylogeny [82,83] and used the Felsenstein-84 model of substitution with no clock, and with alpha and kappa parameters estimated from the data. For each taxon branch lengths were summed from root to tip for each CNE genomic region. To determine which genomic regions account for most of the variation within the sample of summed branch lengths we undertook a Principal Component Analysis (PCA) in PASTv2.16 [84] using the co-variation matrix, in order to visualise the variance of the sample. Multispecies alignments corresponding to six genomic regions were excluded based on either >10% missing data (*UNC4, PRDM16* and *SHOX*) or a missing taxon (*SOX5*, *FOXB1* and *SOX11*). To assess which bat branches were associated with changes in CNEs substitution rates, bar-plots were produced displaying estimated substitution rates along each ancestral and tip bat branch.

### Analysis of vertebrate CNEs conservation or accelerated evolution

To identify specific sites that had undergone statistically significant levels of either conservation or acceleration in focal clades we conducted a statistical test utilising phylogenetic information. We first fitted a neutral substitution model to each concatenated alignment of CNEs representing each genomic region using the software phyloFit [85]. Likelihood ratio tests (LRT) were then performed in phyloP [86], comparing the fit of the neutral model to one that allows either accelerated evolution or conservation (“CON-ACC”) at each site. By this method sites having undergone significant levels of either conservation or accelerated evolution across either all mammals or within particular predefined sub-clades were identified. These analyses were performed within RPHAST [87]. To visualise the distribution of these sites, the proportion of sites falling into either significant acceleration or conservation were calculated based on *P*-values and CON-ACC scores.

#### (B) Mammalian-specific CNEs

##### BASEML relative rates

We used BASEML in the PAML4.4 package to estimate the relative rates of CNE sequences in focal echolocating and non-echolocating taxa compared to the background rate which is set at 1.00. Separate datasets were constructed for bats and cetaceans by pruning either bat or cetacean sequences from the 82,064 initial single CNE alignments. Bat alignments were kept if they contained sequences for all nine bat species. Duplicate alignment datasets were constructed containing either the two echolocating Yinpterochiroperan (*R. ferrumequinum* and *M. lyra*); the three non-echolocating Old World fruit bats (*P. vampyrus, P. alecto* and *E. helvum*) or the four echolocating Yangochiroptera (*P. parnellii, M. lucifugus, Myotis davidii* and *Eptesicus fuscus*). Individual CNE alignments meeting these criteria were concatenated based on the nearest gene; to give a final dataset of a maximum of 5,726 genomic regions that also contained sequences for cow, horse, dog, panda, hedgehog, shrew, human, guinea pig and mouse which were set as outgroup taxa. Due to a large proportion of missing data, three alignments were excluded to give final datasets of 5,726, 5,725 and 5,724 for Old World fruit bats, Yangochiroptera and echolocating Yinpterochiroptera respectively. Cetacean alignments were kept if they contained *T. truncatus* and *B. acutorostrata* sequences. Duplicate alignment datasets were constructed containing either the toothed or baleen whale and the out-group taxa. Individual CNE alignments meeting these criteria were concatenated based on the nearest gene, to give a final dataset of 6,437 genomic regions that also contained sequences for all nine outgroup species.

### Mammalian CNEs proximally located to hearing and ear development genes

Auditory system development and hearing/deafness candidate genes were collated from published sources and online databases [88] (see Additional file 1: Table S5). We then searched our alignments for the corresponding CNEs located near to these genes based on the annotations provided by Kim and Pritchard [32] which uses the human genome as a reference point. These alignments were checked by eye for alignment errors, poorly aligned regions were discarded. The previously calculated relative rates of these regions were then visualised with histograms and radar plots.

### Study of CNEs in the Hmx2/3 gene region of echolocating taxa

For a more in-depth examination of sequence variance in putative ear development CNEs in echolocating species, the human sequences for the 24 CNEs in this genomic region (conserved with ≥60% identity and ≥100 base pairs), were used as blastn queries in four additional cetacean (*B. acutorostrata*, *Orcinus orca*, *Lipotes vexillifer* [89] and *Physeter catodon*) and three bat (*M. davidii* and *E. fuscus* and *P. alecto*) genomes using the same criteria as previously. Lineage-specific rates of substitution were then calculated with BASEML, with the same model settings as previously described, and a set species tree topology [90-92]. In addition, to identify specific sites that had undergone statistically significant levels of acceleration in our focal clades of echolocating taxa we repeated the “CON-ACC” analyses, as described previously, with phyloP [86]. We visualised the number of the significantly accelerated sites based on a negative CON-ACC score and negative *P*-values ≥ −0.05.

Additionally we sequenced four CNEs, for which it was possible to design degenerate primers, from the *Hmx2/3* gene region in a wider range of species. The flanking genomic regions of the four CNEs were downloaded for *M. lucifugus*, *E. caballus*, *Felis catus*, *C. familiaris*, *B. taurus*, *H. sapiens*, *Oryctolagus cuniculus* and *M. musculus* from GenBank. These data were used to build multispecies alignments from which we designed degenerate primers using ‘Primer3’ [93] (Additional file 1: Table S5a). DNA was extracted using Qiagen DNeasy kits and target fragments were amplified using touch-down polymerase chain reaction (PCR) with the following steps: 95°C for 5 minutes; 95°C for 30 seconds; 60–50°C for 30 seconds and 72°C for 1 minute, for 45 cycles run on a MJ Research PTC225 Peltier thermocycler. The total volume of each reaction mix was 15 μL, containing ~25 ng of gDNA, 1.5 μL 10× PCR buffer, 1.2-1.5 μL (25 mM) MgCl_2_, 0.5 μL (10 μM) dNTPs, 1 μL (10 μM) each of forward and reverse primers, 0.1 μL (FastStart Taq DNA polymerase (Roche) and 4.4-4.7 ddH_2_0. Successfully amplified products were purified using ExoSap and sequenced using BigDye v3.1 and visualized on an ABI 3700 automated DNA sequencer. Directly sequenced samples were added to the multiple alignments of published sequences for all available placental mammal species (see Additional file 1: Table S5b for species). Lineage-specific rates of substitution were then calculated for each of the four alignments with BASEML, with the same model settings as previously described, and a set species tree topology [90-92].

### Availability of supporting data

The data sets supporting the results of this article are available in the Dryad repository http://dx.doi.org/10.5061/dryad.50kd5 [94]. New CNE sequences (>200 bp) are available from GenBank (accession numbers: KM981771–KM981864 and KP017254). Additional statistical values, genetic information and primer sequences are available in Additional file 1.

## Additional files

Additional file 1:
**Additional statistical results, supporting references and sample information for the analyses included in the study.**
Additional file 2: Figure S1. Majority consensus trees calculated for sets of ML trees grouped according to their position on human chromosomes. Echolocating species were represented by the following bats: *R. ferrumequinum*, *M. lyra, P. parnellii, M. lucifugus, M. davidii* and *E. fuscus* and the echolocating dolphin (*T. truncatus*)*.* For comparison we included the non-echolocating Old World fruit bats *P. vampyrus, P. alecto* and *E. helvum*; and the baleen minke whale (*B. acutorostrata*). Bats groups are designated by the following colours: echolocating Yinpterochiroptera – green; non-echolocating Yinpterochiroptera – black; Yangochiroptera – brown. A total of 6,109 alignments contained sequence information for all 20 taxa and were used to construct the ML phylogenies. Number of trees used per chromosome: Chr1: 572; Chr2: 561; Chr3: 443; Chr4: 320; Chr5: 424; Chr6: 375; Chr7: 357; Chr8: 293; Chr9: 267; Chr10: 314; Chr11: 341; Chr12: 348; Chr13: 89; Chr14: 282; Chr15: 171; Chr16: 169; Chr17: 302; Chr18: 148; Chr19: 55; Chr20: 157; Chr21: 57; Chr22: 63). Additional file 3: Figure S2. PCA loadings along (A) PC–1 (B) PC–2 and (C) PC–3 for summed root to tip branch lengths of 83 concatenated CNE alignments across 26 mammalian species. Additional file 4: Figure S3. Branch-specific substitution rates across CNEs from 83 genomic regions. (A) Bat branches: (i) ancestral echolocating Yinpterochiroptera branch; (ii) *R. ferrumequinum*; (iii) *M. lyra*; (iv) ancestral Old World fruit bat branch; (v) *E. helvum*; (vi) *P. vampyrus*; (vii) ancestral Yangochiroptera branch; (viii) *M. lucifugus*; (ix) *P. parnellii*; (x) ancestral common bat branch; (xi) ancestral Yinpterochiroptera branch; (xii) graphical representation of the branches under study. (B) Cetartiodactyla branches: (i) ancestral Cetartiodactyla branch (ii) *B. taurus*; (iii) *T. truncatus*. Additional file 5: Figure S4. Levels of acceleration and conservation across genomic regions as estimated by phyloP scores. (A) across all mammals included in the study; (B) all bats included in the study; (C) Old World fruit bat sub-clade; (D) echolocating Yinpterochiroptera sub-clade; (E) Yangochiroptera sub-clade. Colours indicate the percentage of sites calculated to show accelerated evolution (light blue); accelerated evolution with negative *P > −*0.05 (dark blue); ‘neutral’ score = 0 (white); conservation (orange); conservation with *P* < 0.05 (red). Genomic regions containing genes associated with auditory system development are indicated with *. Additional file 6: Figure S5. Frequency histograms of estimated relative rates across mammalian specific CNEs grouped by nearest gene – in each analysis the rate for the foreground clade of interest in given relative to the background rate, which is equal to 1. Rates were calculated in BASEML using the Felsenstein-84 model of substitution, local clock and alpha and kappa estimated from the data (A) Old World fruit bats; (B) echolocating Yinpterochiroptera; (C) Yangochiroptera; (D) Common minke whale and (E) Bottlenose dolphin. Additional file 7: Figure S6. Estimated lineage-specific nucleotide substitution rates across 24 CNE sequence alignments from the *Hmx2/3* gene region. Rates were calculated in BASEML using the Felsenstein-84 model of substitution, no clock and alpha and kappa estimated from the data. (A) The approximate genomic location (in kilo-bases) of the 24 CNEs (white blocks) under study and the three proximate genes (yellow blocks; *HMX3*, *HMX2* and *BUB3* – left to right), also present in this region is RP11-162A23.5 (grey block) which is a pseudogene, based on the *H. sapiens* genome (Ensembl release 75). (B) Estimated branch lengths with fixed species topology calculated from alignments of CNEs from the Hmx2/3 genomic region, the order of trees (left to right, top to bottom) match the numbered CNEs from above (9703 – 9757). Non-focal branches (dark grey), bat branches are coloured as follows: Old World fruit bats (black); echolocating Yinpterochiroptera (green); Yangochiroptera (brown) and cetacean branches are coloured as follows: non-echolocating minke whale (light grey) and echolocating toothed whales (blue). Additional file 8: Figure S7. Number of significantly accelerated sites for 24 CNE sequence alignments from the *Hmx2/3* gene region for (A) bats and (B) cetaceans as calculated by phyloP. (A) Coloured bars represent echolocating Yinpterochiroptera (green); Old World fruit bats (black) and Yangochiroptera (brown). (B) Coloured bars represent all cetaceans (white); echolocating toothed whales (blue) and minke whale (light grey). (C) Approximate genomic location (in kilo-bases) of the 24 CNEs (white blocks) under study and the three proximate genes (yellow blocks; *HMX3*, *HMX2* and *BUB3* – left to right) – see legend of Additional file 7: Figure S6 for full details.

## References

[1] Woolfe A, Goodson M, Goode DK, Snell P, McEwen GK, Vavouri T, Smith SF, North P, Callaway H, Kelly K, Walter K, Abnizova I, Gilks W, Edwards YJK, Cooke JE, Elgar G (2005). Highly conserved non-coding sequences are associated with vertebrate development. PLoS Biol.

[2] Maas SA, Fallon JF (2005). Single base pair change in the long-range Sonic hedgehog limb-specific enhancer is a genetic basis for preaxial polydactyly. Dev Dyn.

[3] Benko S, Fantes JA, Amiel J, Kleinjan DJ, Thomas S, Ramsay J, Jamshidi N, Essafi A, Heaney S, Gordon CT, McBride D, Golzio C, Fisher M, Perry P, Abadie V, Ayuso C, Holder-Espinasse M, Kilpatrick N, Lees MM, Picard A, Temple IK, Thomas P, Vazquez MP, Vekemans M, Crollius HR, Hastie ND, Munnich A, Etchevers HC, Pelet A, Farlie PG (2009). Highly conserved non-coding elements on either side of *SOX9* associated with Pierre Robin sequence. Nat Genet.

[4] Cretekos CJ, Wang Y, Green ED, Martin JF, Rasweiler JJ, Behringer RR, Progra NCS (2008). Regulatory divergence modifies limb length between mammals. Genes Dev.

[5] Sumiyama K, Miyake T, Grimwood J, Stuart A, Dickson M, Schmutz J, Ruddle FH, Myers RM, Amemiya CT (2012). Theria-specific homeodomain and *cis*-regulatory element evolution of the *Dlx3-4* bigene cluster in twelve different mammalian species. J Exp Zool B Mol Dev Evol.

[6] Chan YF, Marks ME, Jones FC, Villarreal G, Shapiro MD, Brady SD, Southwick AM, Absher DM, Grimwood J, Schmutz J, Myers RM, Petrov D, Jónsson B, Schluter D, Bell MA, Kingsley DM (2010). Adaptive evolution of pelvic reduction in sticklebacks by recurrent deletion of a *Pitx1* enhancer. Science.

[7] Nobrega MA, Zhu YW, Plajzer-Frick I, Afzal V, Rubin EM (2004). Megabase deletions of gene deserts result in viable mice. Nature.

[8] Hiller M, Schaar BT, Bejerano G (2012). Hundreds of conserved non-coding genomic regions are independently lost in mammals. Nucleic Acids Res.

[9] Koch CT, Bruggmann R, Tetens J, Drogemuller C (2013). **A non-coding genomic duplication at the*****HMX1*****locus is associated with crop ears in highland cattle**. PLoS ONE.

[10] Quina LA, Kuramoto T, Luquetti DV, Cox TC, Serikawa T, Turner EE (2012). Deletion of a conserved regulatory element required for Hmx1 expression in craniofacial mesenchyme in the dumbo rat: a newly identified cause of congenital ear malformation. Dis Model Mech.

[11] Siepel A, Bejerano G, Pedersen JS, Hinrichs AS, Hou MM, Rosenbloom K, Clawson H, Spieth J, Hillier LW, Richards S, Weinstock GM, Wilson RK, Gibbs RA, Kent WJ, Miller W, Haussler D (2005). Evolutionarily conserved elements in vertebrate, insect, worm, and yeast genomes. Genome Res.

[12] Mikkelsen TS, Wakefield MJ, Aken B, Amemiya CT, Chang JL, Duke S, Garber M, Gentles AJ, Goodstadt L, Heger A, Jurka J, Kamal M, Mauceli E, Searle SMJ, Sharpe T, Baker ML, Batzer MA, Benos PV, Belov K, Clamp M, Cook A, Cuff J, Das R, Davidow L, Deakin JE, Fazzari MJ, Glass JL, Grabherr M, Greally JM, Gu WJ (2007). Genome of the marsupial *Monodelphis domestica* reveals innovation in non-coding sequences. Nature.

[13] Hillier LW, Miller W, Birney E, Warren W, Hardison RC, Ponting CP, Bork P, Burt DW, Groenen MAM, Delany ME, Dodgson JB, Chinwalla AT, Cliften PF, Clifton SW, Delehaunty KD, Fronick C, Fulton RS, Graves TA, Kremitzki C, Layman D, Magrini V, McPherson JD, Miner TL, Minx P, Nash WE, Nhan MN, Nelson JO, Oddy LG, Pohl CS, Randall-Maher J (2004). Sequence and comparative analysis of the chicken genome provide unique perspectives on vertebrate evolution. Nature.

[14] Nelson AC, Wardle FC (2013). Conserved non-coding elements and *cis* regulation: actions speak louder than words. Development.

[15] Hemberg M, Gray JM, Cloonan N, Kuersten S, Grimmond S, Greenberg ME, Kreiman G (2012). Integrated genome analysis suggests that most conserved non-coding sequences are regulatory factor binding sites. Nucleic Acids Res.

[16] Woolfe A, Goode DK, Cooke J, Callaway H, Smith S, Snell P, McEwen GK, Elgar G (2007). CONDOR: a database resource of developmentally associated conserved non-coding elements. BMC Dev Biol.

[17] Engstrom PG, Fredman D, Lenhard B (2008). Ancora: a web resource for exploring highly conserved noncoding elements and their association with developmental regulatory genes. Genome Biol.

[18] Pauls S, Smith SF, Elgar G (2012). Lens development depends on a pair of highly conserved *Sox21* regulatory elements. Dev Biol.

[19] Vater M, Kossl M (2011). Comparative aspects of cochlear functional organization in mammals. Hear Res.

[20] Davies KTJ, Maryanto I, Rossiter SJ (2013). Evolutionary origins of ultrasonic hearing and laryngeal echolocation in bats inferred from morphological analyses of the inner ear. Front Zool.

[21] Cantos R, Cole LK, Acampora D, Simeone A, Wu DK (2000). Patterning of the mammalian cochlea. Proc Natl Acad Sci U S A.

[22] Luo ZX, Ruf I, Schultz JA, Martin T (2011). Fossil evidence on evolution of inner ear cochlea in Jurassic mammals. Proc R Soc B.

[23] Fekete DM (1999). Development of the vertebrate ear: insights from knockouts and mutants. Trends Neurosci.

[24] Hadrys T, Braun T, Rinkwitz-Brandt S, Arnold HH, Bober E (1998). *Nkx5-1* controls semicircular canal formation in the mouse inner ear. Development.

[25] Barrionuevo F, Naumann A, Bagheri-Fam S, Speth V, Taketo MM, Scherer G, Neubueser A (2008). *Sox9* is required for invagination of the otic placode in mice. Dev Biol.

[26] Cox GA, Mahaffey CL, Nystuen A, Letts VA, Frankel WN (2000). The mouse fidgetin gene defines a new role for AAA family proteins in mammalian development. Nat Genet.

[27] Burton Q, Cole LK, Mulheisen M, Chang W, Wu DK (2004). The role of *Pax2* in mouse inner ear development. Dev Biol.

[28] Kiernan AE, Pelling AL, Leung KKH, Tang ASP, Bell DM, Tease C, Lovell-Badge R, Steel KP, Cheah KSE (2005). *Sox2* is required for sensory organ development in the mammalian inner ear. Nature.

[29] Puschel AW, Westerfield M, Dressler GR (1992). Comparative-analysis of Pax-2 protein distributions during neurulation in mice and zebrafish. Mech Dev.

[30] Phillips CD, Butler B, Fondon JWI, Mantilla-Meluk H, Baker RJ (2013). Contrasting evolutionary dynamics of the developmental regulator PAX9, among bats, with evidence for a novel post-transcriptional regulatory mechanism. PLoS ONE.

[31] Teeling EC, Scally M, Kao DJ, Romagnoli ML, Springer MS, Stanhope MJ (2000). Molecular evidence regarding the origin of echolocation and flight in bats. Nature.

[32] Kim SY, Pritchard JK (2007). Adaptive evolution of conserved noncoding elements in mammals. PLoS Genet.

[33] Tajima F (1993). Simple methods for testing the molecular evolutionary clock hypothesis. Genetics.

[34] Zhang G, Cowled C, Shi Z, Huang Z, Bishop-Lilly KA, Xiaodong F, Wynne JW, Xiong Z, Baker ML, Zhao W, Tachedjian M, Zhu Y, Zhou P, Jiang X, Ng J, Yang L, Wu L, Xiao J, Feng Y, Chen Y, Sun X, Zhang Y, Marsh GA, Crameri G, Broder CC, Frey KG, Wang L-F, Wang J (2013). Comparative analysis of bat genomes provides insight into the evolution of flight and immunity. Science.

[35] Parker J, Tsagkogeorga G, Cotton JA, Liu Y, Provero P, Stupka E, Rossiter SJ (2013). Genome-wide signatures of convergent evolution in echolocating mammals. Nature.

[36] Shen YY, Liang L, Li GS, Murphy RW, Zhang YP (2012). Parallel evolution of auditory genes for echolocation in bats and toothed whales. PLoS Genet.

[37] Chatterjee S, Krausl P, Lufkin T (2010). A symphony of inner ear developmental control genes. BMC Genet.

[38] Nishihara H, Hasegawa M, Okada N (2006). Pegasoferae, an unexpected mammalian clade revealed by tracking ancient retroposon insertions. Proc Natl Acad Sci U S A.

[39] Tsagkogeorga G, Parker J, Stupka E, Cotton JA, Rossiter SJ (2013). Phylogenomic analyses elucidate the evolutionary relationships of bats. Curr Biol.

[40] Liu W, Li G, Chien JS, Raft S, Zhang H, Chiang C, Frenz DA (2002). Sonic Hedgehog regulates otic capsule chondrogenesis and inner ear development in the mouse embryo. Dev Biol.

[41] Lettice LA, Heaney SJH, Purdie LA, Li L, de Beer P, Oostra BA, Goode D, Elgar G, Hill RE, de Graaff E (2003). A long-range *Shh* enhancer regulates expression in the developing limb and fin and is associated with preaxial polydactyly. Hum Mol Genet.

[42] Bellusci S, Furuta Y, Rush MG, Henderson R, Winnier G, Hogan BL (1997). Involvement of Sonic hedgehog (*Shh*) in mouse embryonic lung growth and morphogenesis. Development.

[43] Coré N, Caubita X, Metchata A, Bonedb A, Djabalib M, Fasanoa L (2007). *Tshz1* is required for axial skeleton, soft palate and middle ear development in mice. Dev Biol.

[44] Manley GA (2010). An evolutionary perspective on middle ears. Hear Res.

[45] Kinkel MD, Thewissen JGM, Oelschläger HA (2001). Rotation of middle ear ossicles during cetacean development. J Morphol.

[46] Vater M, Kossl M, Foeller E, Coro F, Mora E, Russell IJ (2003). Development of echolocation calls in the mustached bat, *Pteronotus parnellii*. J Neurophysiol.

[47] Davies KTJ, Cotton JA, Kirwan JD, Teeling EC, Rossiter SJ (2012). Parallel signatures of sequence evolution among hearing genes in echolocating mammals: an emerging model of genetic convergence. Heredity.

[48] Li G, Wang JH, Rossiter SJ, Jones G, Cotton JA, Zhang SY (2008). The hearing gene *Prestin* reunites echolocating bats. Proc Natl Acad Sci U S A.

[49] Liu Y, Han N, Franchini LF, Xu H, Pisciottano F, Elgoyhen AB, Rajan KE, Zhang S (2012). The voltage-gated potassium channel subfamily KQT Member 4 (KCNQ4) displays parallel evolution in echolocating bats. Mol Biol Evol.

[50] Dong D, Lei M, Liu Y, Zhang S (2013). Comparative inner ear transcriptome analysis between the Rickett’s big-footed bats (*Myotis ricketti*) and the greater short-nosed fruit bats (*Cynopterus sphinx*). BMC Genomics.

[51] Schmieder DA, Kingston T, Hashim R, Siemers BM (2010). Breaking the trade-off: rainforest bats maximize bandwidth and repetition rate of echolocation calls as they approach prey. Biol Lett.

[52] Waters DA, Jones G (1995). Echolocation call structure and intensity in five species of insectivorous bats. J Exp Biol.

[53] Kalitsis P, Earle E, Fowler KJ, Choo KHA (2000). *Bub3* gene disruption in mice reveals essential mitotic spindle checkpoint function during early embryogenesis. Genes Dev.

[54] Wang WD, Chan EK, Baron S, Van de Water T, Lufkin T (2001). *Hmx2* homeobox gene control of murine vestibular morphogenesis. Development.

[55] Wang WD, Lufkin T (2005). *Hmx* homeobox gene function in inner ear and nervous system cell-type specification and development. Exp Cell Res.

[56] Wang W, Grimmer JF, Van De Water TR, Lufkin T (2004). *Hmx2* and *Hmx3* homeobox genes direct development of the murine inner ear and hypothalamus and can be functionally replaced by *Drosophila Hmx*. Dev Cell.

[57] Miller ND, Nance MA, Wohler ES, Hoover-Fong JE, Lisi E, Thomas GH, Pevsner J (2009). Molecular (SNP) analyses of overlapping hemizygous deletions of 10q25.3 to 10qter in four patients: evidence for *HMX2* and *HMX3* as candidate genes in hearing and vestibular function. Am J Med Genet Part A.

[58] Davies KTJ, Bates PJJ, Maryanto I, Cotton JA, Rossiter SJ (2013). The evolution of bat vestibular systems in the face of potential antagonistic selection pressures for flight and echolocation. PLoS ONE.

[59] Lack JB, Van Den Bussche R (2010). Identifying the confounding factors in resolving phylogenetic relationships in Vespertilionidae. J Mammal.

[60] Galtier N, Duret L (2007). Adaptation or biased gene conversion? Extending the null hypothesis of molecular evolution. Trends Genet.

[61] Bromham L (2011). The genome as a life-history character: why rate of molecular evolution varies between mammal species. Phil Trans R Soc B.

[62] Janes DE, Chapus C, Gondo Y, Clayton DF, Sinha S, Blatti CA, Organ CL, Fujita MK, Balakrishnan CN, Edwards SV (2011). Reptiles and mammals have differentially retained long conserved noncoding sequences from the amniote ancestor. Genome Biol Evol.

[63] Charlesworth B (2009). Effective population size and patterns of molecular evolution and variation. Nat Rev Genet.

[64] Burland TM, Barrattt EM, Beaumont MA, Racey PA (1999). Population genetic structure and gene flow in a gleaning bat, *Plecotus auritus*. Proc R Soc B.

[65] Storz JF, Bhat HR, Kunz TH (2007). Genetic consequences of polygyny and social structure in an Indian fruit bat, *Cynopterus sphinx.* II. Variance in male mating success and effective population size. Evolution.

[66] Thirstrup JP, Bach LA, Loeschcke V, Pertoldi C (2009). Population viability analysis on domestic horse breeds (*Equus caballus*). J Anim Sci.

[67] Murray C, Huerta-Sanchez E, Casey F, Bradley DG (2010). Cattle demographic history modelled from autosomal sequence variation. Phil Trans R Soc B.

[68] ***Tursiops truncatus ssp. ponticus*****. In: IUCN 2012. IUCN Red List of Threatened Species. Version 2012.2.** [www.iucnredlist.org]. Accessed on 12 April 2013.

[69] Lang M, Hadzhiev Y, Siegel N, Amemiya CT, Parada C, Strähle U, Becker M-B, Müller F, Meyer A (2010). Conservation of *shh cis*-regulatory architecture of the coelacanth is consistent with its ancestral phylogenetic position. EvoDevo.

[70] Lee AP, Kerk SY, Tan YY, Brenner S, Venkatesh B (2011). Ancient vertebrate conserved noncoding elements have been evolving rapidly in teleost fishes. Mol Biol Evol.

[71] Prabhakar S, Noonan JP, Paeaebo S, Rubin EM (2006). Accelerated evolution of conserved noncoding sequences in humans. Science.

[72] Flicek P, Ahmed I, Amode MR, Barrell D, Beal K, Brent S, Carvalho-Silva D, Clapham P, Coates G, Fairley S, Fitzgerald S, Gil L, Garcia-Girón C, Gordon L, Hourlier T, Hunt S, Juettemann T, Kähäri A, Keenan S, Komorowska M, Kulesha E, Longden I, Maurel T, McLaren W, Muffato M, Nag R, Overduin B, Pignatelli M, Pritchard B, Pritchard E (2013). Ensembl 2013. Nucleic Acids Res.

[73] Yim H-S, Cho YS, Guang X, Kang SG, Jeong J-Y, Cha S-S, Oh H-M, Lee J-H, Yang EC, Kwon KK, Kim YJ, Kim TW, Kim W, Jeon JH, Kim S-J, Choi DH, Jho S, Kim H-M, Ko J, Kim H, Shin Y-A, Jung H-J, Zheng Y, Wang Z, Chen Y, Chen M, Jiang A, Li E, Zhang S, Hou H (2014). Minke whale genome and aquatic adaptation in cetaceans. Nat Genet.

[74] Stamatakis A (2006). RAxML-VI-HPC: maximum likelihood-based phylogenetic analyses with thousands of taxa and mixed models. Bioinformatics.

[75] Huson DH, Richter DC, Rausch C, Dezulian T, Franz M, Rupp R (2007). Dendroscope: an interactive viewer for large phylogenetic trees. BMC Bioinformatics.

[76] Tamura K, Peterson D, Peterson N, Stecher G, Nei M, Kumar S (2011). MEGA5: Molecular evolutionary genetics analysis using maximum likelihood, evolutionary distance, and maximum parsimony methods. Mol Biol Evol.

[77] Robinson M, Gouy M, Gautier C, Mouchiroud D (1998). Sensitivity of the relative-rate test to taxonomic sampling. Mol Biol Evol.

[78] Benjamini Y, Hochberg Y (1995). Controlling the false discovery rate – a practical and powerful approach to multiple testing. J Roy Stat Soc B Met.

[79] Holm S (1979). A simple sequentially rejective multiple test procedure. Scand J Stat.

[80] Spitz F, Gonzalez F, Duboule D (2003). A global control region defines a chromosomal regulatory landscape containing the *HoxD* cluster. Cell.

[81] Yang ZH (2007). PAML 4: phylogenetic analysis by maximum likelihood. Mol Biol Evol.

[82] Meredith RW, Janecka JE, Gatesy J, Ryder OA, Fisher CA, Teeling EC, Goodbla A, Eizirik E, Simao TLL, Stadler T, Rabosky DL, Honeycutt RL, Flynn JJ, Ingram CM, Steiner C, Williams TL, Robinson TJ, Burk-Herrick A, Westerman M, Ayoub NA, Springer MS, Murphy WJ (2011). Impacts of the cretaceous terrestrial revolution and KPg extinction on mammal diversification. Science.

[83] Zhou X, Xu S, Xu J, Chen B, Zhou K, Yang G (2012). Phylogenomic analysis resolves the interordinal relationships and rapid diversification of the Laurasiatherian mammals. Syst Biol.

[84] Hammer Ø, Harper DAT, Ryan PD (2001). PAST: paleontological statistics software package for education and data analysis. Palaeontol Electron.

[85] Siepel A, Haussler D (2004). Phylogenetic estimation of context-dependent substitution rates by maximum likelihood. Mol Biol Evol.

[86] Pollard KS, Hubisz MJ, Rosenboom K, Siepel A (2010). Detection of non-neutral substitution rates on mammalian phylogenies. Genome Res.

[87] Hubisz MJ, Pollard KS, Siepel A (2011). PHASTand RPHAST: phylogenetic analysis with space/time models. Brief Bioinform.

[88] **The Hereditary Hearing loss Homepage.** [http://hereditaryhearingloss.org]

[89] Zhou X, Sun F, Xu S, Fan G, Zhu K, Liu X, Chen Y, Shi C, Yang Y, Huang Z, Chen J, Hou H, Guo X, Chen W, Chen Y, Wang X, Lv T, Yang D, Zhou J, Huang B, Wang Z, Zhao W, Tian R, Xiong Z, Xu J, Liang X, Chen B, Liu W, Wang J, Pan S (2013). Baiji genomes reveal low genetic variability and new insights into secondary aquatic adaptations. Nat Commun.

[90] Csorba G, Ujhelyi P, Thomas N (2003). Horseshoe bats of the world (Chiroptera: Rhinolophidae).

[91] Giannini NP, Simmons NB (2003). A phylogeny of megachiropteran bats (Mammalia: Chiroptera: Pteropodidae) based on direct optimization analysis of one nuclear and four mitochondrial genes. Cladistics.

[92] Khan FAA, Solari S, Swier VJ, Larsen PA, Abdullah MT, Baker RJ (2010). Systematics of Malaysian woolly bats (Vespertilionidae: *Kerivoula*) inferred from mitochondrial, nuclear, karyotypic, and morphological data. J Mammal.

[93] Rozen S, Skaletsky H (2000). Primer3 on the WWW for general users and for biologist programmers. Methods Mol Biol.

[94] Davies KTJ, Tsagkogeorga G, Rossiter SJ: **Data from: Divergent evolutionary rates in vertebrate and mammalian specific Conserved Non-coding Elements (CNEs) in echolocating mammals.** *In Dryad Data Repository* 2014, http://dx.doi.org/10.5061/dryad.50kd5. 10.1186/s12862-014-0261-5 PMC4302572 25523630

