# Supplementary material for: Divergent evolutionary rates in vertebrate and mammalian specific conserved non-coding elements (CNEs) in echolocating mammals
Source: BMC Evol Biol. 2014 Dec 19;14:261. doi: 10.1186/s12862-014-0261-5 (PMC4302572; doi:10.1186/s12862-014-0261-5)
Supplement: Additional file 1: — Additional statistical results, supporting references and sample information for the analyses included in the study. [file 12862_2014_261_MOESM1_ESM.pdf]

Additional file 1: Divergent evolutionary rates in vertebrate and mammalian specific Conserved Non-coding Elements (CNEs) in echolocating mammals

Table S1.
(a) Species information and sequence coverage of the 3,110 vertebrate specific CNEs included in this study. Species denoted by * were found to contain >10% missing data of vertebrate CNEs and were excluded from initial vertebrate-specific CNE analyses.
Data sources and associated references: Ensembl [1]; CONDOR database [2]; GenBank bat genome assembly numbers [3].

Superorder	Order	Family	Species	Common name	% missing data	Data source:
	Monotremata	Ornithorhynchidae	*Ornithorhynchus anatinus*	Platypus	6	Ensembl [1]
Marsupialia	Didelphimorphia	Didelphidae	*Monodelphis domestica*	Grey short-tailed opossum	4	Ensembl [1]
		Macropodidae	*Macropus eugenii**	Wallaby	18	Ensembl [1]
	Dasyuromorphia	Dasyuridae	*Sarcophilus harrisii*	Tasmanian devil	3	Ensembl [1]
Xenarthra	Cingulata	Dasypodidae	*Dasypus novemcinctus**	Nine-banded armadillo	19	Ensembl [1]
Afrotheria	Pilosa	Megalonychidae	*Choloepus hoffmanni**	Two-toed sloth	19	Ensembl [1]
	Proboscidea	Elephantidae	*Loxodonta africana*	African elephant	1	Ensembl [1]
	Afrosoricida	Tenrecidae	*Echinops telfairi**	Madagascan hedgehog tenrec	16	Ensembl [1]
	Hyracoidea	Procaviidae	*Procavia capensis**	Rock Hyrax	14	Ensembl [1]
Laurasiatheria	Erinaceomorpha	Erinaceidae	*Erinaceus europaeus**	European hedgehog	18	Ensembl [1]
	Soricomorpha	Soricidae	*Sorex araneus**	European shrew	20	Ensembl [1]
	Chiroptera	Pteropodidae	*Pteropus vampyrus*	Large flying fox	7	Ensembl [1]
			Eidolon helvum	Straw coloured fruit bat	3	ASM46528v1[3]
		Rhinolophidae	*Rhinolophus ferrumequinum*	Greater horseshoe bat	1	ASM46549v1[3]
		Megadermatidae	*Megaderma lyra*	Greater false vampire bat	10	ASM46534v1[3]
		Mormoopidae	*Pteronotus parnellii*	Parnell's moustached bat	5	ASM46540v1[3]
		Vespertilionidae	*Myotis lucifugus*	Little brown bat	5	Ensembl [1]
	Cetartiodactyla	Camelidae	*Vicugna pacos**	Alpaca	16	Ensembl [1]
		Bovidae	*Bos taurus*	Cow	2	Ensembl [1]
			*Ovis aries**	Sheep	14	Ensembl [1]

		Suidae	*Sus scrofa**	Pig	15	Ensembl [1]
	Cetacea	Delphinidae	*Tursiops truncatus*	Bottlenose dolphin	4	Ensembl [1]
	Carnivora	Ursidae	*Ailuropoda melanoleuca*	Panda	<1	Ensembl [1]
		Felidae	*Felis catus**	Cat	25	Ensembl [1]
		Canidae	*Canis familiaris*	Dog	1	Ensembl [1]
	Perissodactyla	Equidae	*Equus caballus*	Horse	1	Ensembl [1]
Euarchontoglires	Scandentia	Tupaiidae	*Tupaia belangeri**	Northern treeshrew	13	Ensembl [1]
	Primata	Hominidae	*Gorilla gorilla*	Gorilla	3	Ensembl [1]
			Homo sapiens	Human	0	CONDOR [2]
			Pan troglodytes	Chimpanzee	<1	Ensembl [1]
			Pongo abelii	Sumatran orangutan	1	Ensembl [1]
		Hylobatidae	*Nomascus leucogenys*	Northern white-cheeked gibbon	<1	Ensembl [1]
		Cercopithecidae	*Macaca mulatta*	Rhesus macaque	<1	Ensembl [1]
			Papio hamadryas	Hamadryas baboon	<1	Ensembl [1]
		Cebidae	*Callithrix jacchus*	Marmoset	<1	Ensembl [1]
		Galagidae	*Otolemur garnettii**	Greater galago	13	Ensembl [1]
		Cheirogaleidae	*Microcebus murinus**	Mouse lemur	11	Ensembl [1]
		Tarsiidae	*Tarsius syrichta**	Tarsier	11	Ensembl [1]
	Lagomorpha	Leporidae	*Oryctolagus cuniculus*	Rabbit	3	Ensembl [1]
		Ochotonidae	*Ochotona princeps**	Pika	12	Ensembl [1]
	Rodentia	Muridae	*Mus musculus*	Mouse	2	CONDOR [2]
			*Rattus norvegicus**	Rat	16	CONDOR [2]
		Heteromyidae	*Dipodomys ordii**	Kangaroo rat	12	Ensembl [1]
		Sciuridae	*Ictidomys tridecemlineatus**	Thirteen-lined ground squirrel	19	Ensembl [1]
		Caviidae	*Cavia porcellus*	Guinea pig	2	Ensembl [1]

(b) Species information for taxa included in wider sampling of CNEs from the Hmx2/3 genomic region.
Data sources and associated references: Ensembl [1]; CONDOR database [2]; GenBank genome assembly numbers [3-6].
* Provided by Wesley Warren and The Genome Institute, Washington University School of Medicine.

Superorder	Order	Family	Species	Common name	Data source:
	Monotremata	Ornithorhynchidae	*Ornithorhynchus anatinus*	Platypus	Ensembl [1]
Marsupialia	Didelphimorphia	Didelphidae	*Monodelphis domestica*	Grey short-tailed opossum	Ensembl [1]
	Dasyuromorphia	Dasyuridae	*Sarcophilus harrisii*	Tasmanian devil	Ensembl [1]
Afrotheria	Proboscidea	Elephantidae	*Loxodonta africana*	African elephant	Ensembl [1]
Laurasiatheria	Erinaceomorpha	Erinaceidae	*Erinaceus europaeus*	European hedgehog	Ensembl [1]
	Soricomorpha	Soricidae	*Sorex araneus*	European shrew	Ensembl [1]
	Chiroptera	Pteropodidae	*Pteropus vampyrus*	Large flying fox	Ensembl [1]
			Pteropus alecto	Black flying fox	ASM32557v1 [4]
			Eidolon helvum	Straw coloured fruit bat	ASM46528v1 [3]
		Rhinolophidae	*Rhinolophus ferrumequinum*	Greater horseshoe bat	ASM46549v1 [3]
		Megadermatidae	*Megaderma lyra*	Greater false vampire bat	ASM46534v1 [3]
		Mormoopidae	*Pteronotus parnellii*	Parnell's moustached bat	ASM46540v1 [3]
		Vespertilionidae	*Myotis lucifugus*	Little brown bat	Ensembl [1]
			Myotis davidii	David's Myotis	ASM32557v1 [4]
			Eptesicus fuscus	Big brown bat	EptFus1.0
	Cetartiodactyla	Bovidae	*Bos taurus*	Cow	Ensembl [1]
	Cetacea	Balaenopteridae	*Balaenoptera acutorostrata*	Minke whale	BalAcu1.0 [5]
		Delphinidae	*Tursiops truncatus*	Bottlenose dolphin	Ensembl [1]
			Orcinus orca	Killer whale	Oorc_1.1
		Physeteridae	*Physeter catodon*	Sperm whale	Physeter_macrocephalus-2.0.2*
		Lipotidae	*Lipotes vexillifer*	River dolphin	Lipotes_vexillifer_v1 [6]
	Carnivora	Ursidae	*Ailuropoda melanoleuca*	Panda	Ensembl [1]
		Canidae	*Canis familiaris*	Dog	Ensembl [1]
	Perissodactyla	Equidae	*Equus caballus*	Horse	Ensembl [1]
Euarchontoglires	Primata	Hominidae	*Gorilla gorilla*	Gorilla	Ensembl [1]

			Homo sapiens	Human	CONDOR [2]
			Pan troglodytes	Chimpanzee	Ensembl [1]
			Pongo abelii	Sumatran orangutan	Ensembl [1]
		Hylobatidae	*Nomascus leucogenys*	Northern white-cheeked gibbon	Ensembl [1]
		Cercopithecidae	*Macaca mulatta*	Rhesus macaque	Ensembl [1]
			Papio hamadryas	Hamadryas baboon	Ensembl [1]
		Cebidae	*Callithrix jacchus*	Marmoset	Ensembl [1]
	Lagomorpha	Leporidae	*Oryctolagus cuniculus*	Rabbit	Ensembl [1]
	Rodentia	Muridae	*Mus musculus*	Mouse	CONDOR [2]
		Caviidae	*Cavia porcellus*	Guinea pig	Ensembl [1]

(c) Species representation and sources used for the mammalian specific CNE study.

Data sources and associated references: Ensembl [1]; UCSC genome browser: [http://genome.ucsc.edu/] [7]: GenBank genome assembly numbers [3-5].

Superorder	Order	Family	Species	Common name	Number of CNEs	Data:
Laurasiatheria	Erinaceomorpha	Erinaceidae	*Erinaceus europaeus*	European hedgehog	52,201	Ensembl [1]
	Soricomorpha	Soricidae	*Sorex araneus*	European shrew	49,554	Ensembl [1]
	Chiroptera	Pteropodidae	*Pteropus vampyrus*	Large flying fox	74,119	Ensembl [1]
			Pteropus alecto	Black flying fox	78,665	ASM32557v1 [4]
			Eidolon helvum	Straw coloured fruit bat	77,923	ASM46528v1 [3]
		Rhinolophidae	*Rhinolophus ferrumequinum*	Greater horseshoe bat	79,525	ASM46549v1 [3]
		Megadermatidae	*Megaderma lyra*	Greater false vampire bat	71,534	ASM46534v1 [3]
		Mormoopidae	*Pteronotus parnellii*	Parnell's moustached bat	74,876	ASM46540v1 [3]
		Vespertilionidae	*Myotis lucifugus*	Little brown bat	72,321	Ensembl [1]
			Myotis davidii	David's Myotis	72,628	ASM32557v1 [4]
			Eptesicus fuscus	Big brown bat	73,265	EptFus1.0
	Cetartiodactyla	Bovidae	*Bos taurus*	Cow	78,564	Ensembl [1]
	Cetacea	Delphinidae	*Tursiops truncatus*	Bottlenose dolphin	70,070	Ensembl [1]
		Balaenopteridae	*Balaenoptera acutorostrata*	Minke whale	80,440	BalAcu1.0 [5]
	Carnivora	Ursidae	*Ailuropoda melanoleuca*	Panda	81,356	Ensembl [1]
		Canidae	*Canis familiaris*	Dog	81,658	Ensembl [1]
	Perissodactyla	Equidae	*Equus caballus*	Horse	80,531	Ensembl [1]
Euarchontoglires	Primata	Hominidae	*Homo sapiens*	Human	82,335	hg17 [7]
	Rodentia	Muridae	*Mus musculus*	Mouse	79,227	Ensembl [1]
		Caviidae	*Cavia porcellus*	Guinea pig	72,820	Ensembl [1]

Table S2.

Results of Tajima's relative rates test across all concatenated CNEs. Substitution rates in each focal taxon were compared to the substitution rate of the horse (*E. caballus*), with reference to the human sequence. Statistics given include the uncorrected raw *P*-values, and these corrected for the multiple comparisons made across taxa by the FDR and Holm correction. Abbreviations: FDR – false discovery rate, d.f. – degrees of freedom.

	Sites in sequences:		Unique differences:				*P*-value
Focal species:	Identical	Divergent	Focal species	*E. caballus*	*H. sapiens*	X^2 (1 d.f.)	Raw (FDR, Holm)
A. melanoleuca	537,945	355	5191	4933	5875	6.57	0.010 (0.011, 0.021)
C. familiaris	530,198	335	4594	4837	5783	6.26	0.012 (0.012, 0.021)
B. taurus	524,419	364	6266	4703	5595	222.72	$<1x10^{-5}$ ($1.3x10^{-5}$, $1x10^{-4}$)
T. truncatus	502,412	370	7016	4579	5474	512.20	$<1x10^{-5}$ ($1.3x10^{-5}$, $1x10^{-4}$)
E. helvum	514,758	338	6825	4520	5251	468.31	$<1x10^{-5}$ ($1.3x10^{-5}$, $1x10^{-4}$)
P. vampyrus	485,450	304	6748	4311	4981	537.03	$<1x10^{-5}$ ($1.3x10^{-5}$, $1x10^{-4}$)
R. ferrumequinum	530,780	397	6568	4763	5742	287.53	$<1x10^{-5}$ ($1.3x10^{-5}$, $1x10^{-4}$)
M. lyra	470,970	378	8095	4030	4752	1362.82	$<1x10^{-5}$ ($1.3x10^{-5}$, $1x10^{-4}$)
M. lucifugus	502,675	437	9519	4325	5219	1948.69	$<1x10^{-5}$ ($1.3x10^{-5}$, $1x10^{-4}$)
P. parnellii	498,446	382	7537	4411	5081	817.87	$<1x10^{-5}$ ($1.3x10^{-5}$, $1x10^{-4}$)

Table S3.

Results of Tajima's Relative Rates Test used to test lineage specific substitution rates in CNEs from echolocating and non-echolocating Laurasiatheria species. Substitution rates in each focal taxon were compared to the substitution rate of the horse, with reference to the human sequence. In total 46 concatenated CNE alignments were tested, with each alignment corresponding to the corresponding CNEs from genomic regions containing at least one of the 49 identified ear development genes. Statistics given include the uncorrected raw *P*-values, and these corrected for the multiple comparisons made across loci by the FDR and Holm correction. Abbreviations: = – identical; ≠ – different; FDR – false discovery rate; FT – focal taxon; d.f. – degrees of freedom.

Genomic region:	#Sites:		#Unique differences:			X^2	*P*-values
Focal taxon (FT)	=	≠	FT	Horse	Human	(1 d.f.)	Uncorr. (FDR, Holm)
ATBF1							
Panda	3858	4	25	29	50	0.30	0.586 (0.773, 1.000)
Dog	3865	3	28	29	53	0.02	0.895 (0.895, 1.000)
Cow	3806	0	43	30	60	2.32	0.128 (0.320, 0.896)
Dolphin	3628	2	53	31	50	5.76	0.016 (0.120, 0.160)
E. helvum	3914	2	38	34	52	0.22	0.637 (0.773, 1.000)
P. vampyrus	3195	1	38	25	45	2.68	0.101 (0.320, 0.808)
R. ferrumequinum	3869	2	39	30	54	1.17	0.279 (0.528, 1.000)
M. lyra	3661	2	31	28	52	0.15	0.696 (0.773, 1.000)
M. lucifugus	3932	2	49	29	58	5.13	0.024 (0.120, 0.216)
P. parnellii	3968	2	36	28	63	1.00	0.317 (0.528, 1.000)
BARHL2							
Panda	9770	1	72	78	107	0.24	0.624 (0.624, 1.000)
Dog	9780	2	64	82	110	2.22	0.136 (0.227, 0.680)
Cow	8946	4	93	72	85	2.67	0.102 (0.204, 0.612)
Dolphin	8979	6	111	74	92	7.40	0.007 (0.018, 0.050)
E. helvum	9701	3	69	86	99	1.86	0.172 (0.246, 0.688)
P. vampyrus	9726	3	77	86	96	0.50	0.481 (0.601, 1.000)
R. ferrumequinum	9623	4	91	84	103	0.28	0.597 (0.624, 1.000)
M. lyra	9244	3	137	82	92	13.81	$2x10^{-4}$ (0.001, 0.002)
M. lucifugus	9399	7	141	77	99	18.79	$1x10^{-5}$ (0.000, 0.000)
P. parnellii	9407	7	121	82	92	7.49	0.006 (0.018, 0.050)
BCL11A							
Panda	8472	1	35	23	43	2.48	0.115 (0.128, 0.330)
Dog	8421	3	23	23	41	0.00	1.000 (1.000, 1.000)
Cow	8335	0	36	22	44	3.38	0.066 (0.095, 0.330)
Dolphin	7920	3	66	24	38	19.60	$1x10^{-5}$ (0.000, 0.000)
E. helvum	8177	1	44	23	42	6.58	0.010 (0.020, 0.060)
P. vampyrus	7791	0	60	23	39	16.49	$5x10^{-5}$ (0.000, 0.000)
R. ferrumequinum	8338	1	39	25	41	3.06	0.080 (0.100, 0.330)
M. lyra	7865	1	51	24	41	9.72	0.002 (0.005, 0.013)
M. lucifugus	8354	0	80	25	42	28.81	$<1x10^{-5}$ (0.000, 0.000)
P. parnellii	8306	1	41	26	40	3.36	0.067 (0.095, 0.330)
BCL11B							
Panda	1571	1	4	4	15	0.00	1.000 (1.000, 1.000)
Dog	1563	1	5	4	14	0.11	0.739 (0.821, 1.000)
Cow	1439	1	12	4	12	4.00	0.046 (0.230, 0.460)
Dolphin	1557	1	13	6	12	2.58	0.108 (0.270, 0.756)

E. helvum	1574	1	4	5	14	0.11	0.739 (0.821, 1.000)
P. vampyrus	1477	1	3	5	7	0.50	0.480 (0.686, 1.000)
R. ferrumequinum	1563	1	12	4	15	4.00	0.046 (0.230, 0.460)
M. lyra	1222	1	5	3	9	0.50	0.480 (0.686, 1.000)
M. lucifugus	1555	2	14	6	12	3.20	0.074 (0.247, 0.592)
P. parnellii	1539	1	9	5	14	1.14	0.285 (0.570, 1.000)
BHLHB5							
Panda	4766	2	47	34	56	2.09	0.149 (0.166, 0.366)
Dog	4760	1	28	32	59	0.27	0.606 (0.606, 0.606)
Cow	4715	2	86	34	55	22.53	$<1x10^{-5}$ (0.000, 0.000)
Dolphin	4433	2	71	31	44	15.69	$7x10^{-5}$ (0.000, 0.001)
E. helvum	4794	5	48	34	56	2.39	0.122 (0.153, 0.366)
P. vampyrus	4452	4	42	27	51	3.26	0.071 (0.101, 0.284)
R. ferrumequinum	4772	4	57	33	55	6.40	0.011 (0.018, 0.055)
M. lyra	4758	7	73	33	55	15.09	$1x10^{-4}$ (0.000, 0.001)
M. lucifugus	4749	3	66	36	53	8.82	0.003 (0.006, 0.017)
P. parnellii	4667	6	62	31	54	10.33	0.001 (0.003, 0.009)
DACH1							
Panda	9923	2	26	32	62	0.62	0.431 (0.479, 0.862)
Dog	9918	1	29	30	65	0.02	0.896 (0.896, 0.896)
Cow	9848	2	59	30	61	9.45	0.002 (0.005, 0.015)
Dolphin	9470	4	89	35	55	23.52	$<1x10^{-5}$ (0.000, 0.000)
E. helvum	9642	3	49	35	51	2.33	0.127 (0.159, 0.381)
P. vampyrus	9527	2	54	37	50	3.18	0.075 (0.107, 0.299)
R. ferrumequinum	9775	2	63	37	57	6.76	0.009 (0.019, 0.056)
M. lyra	9338	3	79	34	50	17.92	$2x10^{-5}$ (0.000, 0.000)
M. lucifugus	9772	2	64	40	54	5.54	0.019 (0.031, 0.093)
P. parnellii	9385	2	66	34	52	10.24	0.001 (0.005, 0.011)
DLX1							
Panda	4746	3	38	36	46	0.05	0.816 (0.816, 1.000)
Dog	4726	5	35	32	46	0.13	0.714 (0.798, 1.000)
Cow	3812	4	30	26	33	0.29	0.593 (0.798, 1.000)
Dolphin	4542	7	36	33	42	0.13	0.718 (0.798, 1.000)
E. helvum	4754	6	42	25	54	4.31	0.038 (0.076, 0.228)
P. vampyrus	4647	4	53	25	55	10.05	0.002 (0.010, 0.018)
R. ferrumequinum	4695	3	54	33	50	5.07	0.024 (0.060, 0.168)
M. lyra	4434	2	49	26	48	7.05	0.008 (0.027, 0.064)
M. lucifugus	4652	5	83	32	43	22.62	$<1x10^{-5}$ (0.000, 0.000)
P. parnellii	4615	3	37	23	41	3.27	0.071 (0.118, 0.355)
EBF1							
Panda	1520	1	4	10	10	2.57	0.109 (0.363, 0.872)
Dog	1496	1	6	10	9	1.00	0.317 (0.634, 1.000)
Cow	1511	0	11	10	11	0.05	0.827 (0.919, 1.000)
Dolphin	1339	0	13	9	11	0.73	0.394 (0.657, 1.000)
E. helvum	1509	0	3	10	10	3.77	0.052 (0.260, 0.520)
P. vampyrus	1513	0	3	10	10	3.77	0.052 (0.260, 0.520)
R. ferrumequinum	1506	0	6	10	10	1.00	0.317 (0.634, 1.000)
M. lyra	1504	0	11	8	12	0.47	0.491 (0.701, 1.000)
M. lucifugus	1509	1	9	9	10	0.00	1.000 (1.000, 1.000)
P. parnellii	1477	1	9	10	9	0.05	0.819 (0.919, 1.000)

EMX2							
Panda	6685	5	62	63	71	0.01	0.929 (0.929, 1.000)
Dog	6705	1	45	63	75	3.00	0.083 (0.208, 0.581)
Cow	6618	3	50	57	71	0.46	0.499 (0.713, 1.000)
Dolphin	6187	4	66	58	62	0.52	0.473 (0.713, 1.000)
E. helvum	6629	3	59	69	69	0.78	0.377 (0.713, 1.000)
P. vampyrus	6518	2	61	63	74	0.03	0.857 (0.929, 1.000)
R. ferrumequinum	6610	5	68	64	73	0.12	0.728 (0.910, 1.000)
M. lyra	6325	6	96	60	73	8.31	0.004 (0.013, 0.032)
M. lucifugus	6489	5	143	65	61	29.25	$<1x10^{-5}$ (0.000, 0.000)
P. parnellii	6405	5	100	61	68	9.45	0.002 (0.011, 0.019)
ESRRB							
Panda	3229	0	21	26	49	0.53	0.466 (0.556, 1.000)
Dog	3225	0	18	24	52	0.86	0.355 (0.507, 1.000)
Cow	3222	3	30	25	48	0.45	0.500 (0.556, 1.000)
Dolphin	3177	6	37	23	45	3.27	0.071 (0.178, 0.497)
E. helvum	3180	3	39	27	43	2.18	0.140 (0.233, 0.700)
P. vampyrus	2424	1	27	23	34	0.32	0.572 (0.572, 1.000)
R. ferrumequinum	3197	3	38	25	47	2.68	0.101 (0.202, 0.606)
M. lyra	2877	4	51	24	43	9.72	0.002 (0.020, 0.020)
M. lucifugus	3197	3	46	25	48	6.21	0.013 (0.043, 0.104)
P. parnellii	3067	2	48	25	44	7.25	0.007 (0.035, 0.063)
EVI1							
Panda	2529	0	13	15	14	0.14	0.705 (0.783, 1.000)
Dog	2538	0	11	14	16	0.36	0.549 (0.753, 1.000)
Cow	2586	1	20	16	16	0.44	0.505 (0.753, 1.000)
Dolphin	2582	1	24	17	15	1.20	0.274 (0.753, 1.000)
E. helvum	2450	0	12	16	16	0.57	0.450 (0.753, 1.000)
P. vampyrus	2427	0	17	14	16	0.29	0.590 (0.753, 1.000)
R. ferrumequinum	2588	1	16	17	14	0.03	0.862 (0.862, 1.000)
M. lyra	2567	0	18	15	17	0.27	0.602 (0.753, 1.000)
M. lucifugus	2589	0	22	17	16	0.64	0.423 (0.753, 1.000)
P. parnellii	2489	0	23	15	17	1.68	0.194 (0.753, 1.000)
EYA1							
Panda	3682	4	51	79	73	6.03	0.014 (0.070, 0.126)
Dog	3669	3	49	80	75	7.45	0.006 (0.060, 0.060)
Cow	3559	4	91	78	76	1.00	0.317 (0.396, 0.951)
Dolphin	3417	3	64	65	71	0.01	0.930 (0.930, 0.988)
E. helvum	3513	3	113	81	71	5.28	0.022 (0.073, 0.176)
P. vampyrus	3346	5	107	81	67	3.60	0.058 (0.116, 0.348)
R. ferrumequinum	3590	1	91	82	66	0.47	0.494 (0.549, 0.988)
M. lyra	3554	4	101	78	68	2.96	0.086 (0.143, 0.430)
M. lucifugus	3594	7	89	69	69	2.53	0.112 (0.160, 0.448)
P. parnellii	3667	1	61	85	71	3.95	0.047 (0.116, 0.329)
FIGN							
Panda	8844	2	67	35	51	10.04	0.002 (0.003, 0.008)
Dog	8849	3	67	34	53	10.78	0.001 (0.002, 0.006)
Cow	8801	1	56	40	46	2.67	0.102 (0.102, 0.102)
Dolphin	8234	2	80	35	51	17.61	$3x10^{-5}$ (0.000, 0.000)
E. helvum	8847	3	56	33	55	5.94	0.015 (0.019, 0.045)

P. vampyrus	8213	4	68	32	54	12.96	$3x10^{-4}$ (0.001, 0.002)
R. ferrumequinum	8835	2	54	34	53	4.55	0.033 (0.037, 0.066)
M. lyra	8817	2	84	39	49	16.46	$5x10^{-5}$ (0.000, 0.000)
M. lucifugus	8826	2	76	35	53	15.14	$1x10^{-4}$ (0.000, 0.001)
P. parnellii	8824	3	64	33	54	9.91	0.002 (0.003, 0.008)
FOXP1							
Panda	12080	10	114	101	117	0.79	0.375 (0.375, 0.390)
Dog	12107	3	78	102	121	3.20	0.074 (0.093, 0.222)
Cow	11905	8	169	93	116	22.05	$<1x10^{-5}$ (0.000, 0.000)
Dolphin	11339	7	163	94	105	18.53	$2x10^{-5}$ (0.000, 0.000)
E. helvum	12013	4	137	104	117	4.52	0.034 (0.049, 0.136)
P. vampyrus	11339	3	117	98	109	1.68	0.195 (0.217, 0.390)
R. ferrumequinum	11828	6	205	99	114	36.96	$<1x10^{-5}$ (0.000, 0.000)
M. lyra	8382	9	387	59	59	241.22	$<1x10^{-5}$ (0.000, 0.000)
M. lucifugus	9965	14	417	69	78	249.19	$<1x10^{-5}$ (0.000, 0.000)
P. parnellii	11693	8	281	106	102	79.13	$<1x10^{-5}$ (0.000, 0.000)
GBX2							
Panda	1387	0	13	13	8	0.00	1.000 (1.000, 1.000)
Dog	1390	0	12	12	9	0.00	1.000 (1.000, 1.000)
Cow	1393	1	10	12	10	0.18	0.670 (0.838, 1.000)
Dolphin	1339	0	29	15	6	4.45	0.035 (0.117, 0.297)
E. helvum	1383	0	28	13	10	5.49	0.019 (0.117, 0.190)
P. vampyrus	1394	0	25	12	12	4.57	0.033 (0.117, 0.297)
R. ferrumequinum	1360	0	19	12	12	1.58	0.209 (0.418, 1.000)
M. lyra	802	0	4	11	4	3.27	0.071 (0.178, 0.497)
M. lucifugus	1389	0	16	12	10	0.57	0.450 (0.750, 1.000)
P. parnellii	1180	1	11	14	5	0.36	0.549 (0.784, 1.000)
GLI3							
Panda	2151	0	42	35	31	0.64	0.425 (0.607, 1.000)
Dog	2148	2	31	35	28	0.24	0.622 (0.732, 1.000)
Cow	2161	0	36	38	29	0.05	0.816 (0.816, 1.000)
Dolphin	2137	0	43	39	28	0.20	0.659 (0.732, 1.000)
E. helvum	2036	0	62	35	25	7.52	0.006 (0.023, 0.054)
P. vampyrus	1815	0	61	32	20	9.04	0.003 (0.023, 0.030)
R. ferrumequinum	2048	2	49	30	25	4.57	0.033 (0.083, 0.231)
M. lyra	2146	3	48	35	39	2.04	0.154 (0.308, 0.924)
M. lucifugus	2137	1	47	36	29	1.46	0.227 (0.378, 1.000)
P. parnellii	2103	3	59	33	26	7.35	0.007 (0.023, 0.056)
HMX2							
Panda	5019	3	56	34	56	5.38	0.020 (0.025, 0.060)
Dog	4994	1	48	34	57	2.39	0.122 (0.122, 0.122)
Cow	5027	1	59	35	57	6.13	0.013 (0.019, 0.052)
Dolphin	4835	2	84	30	55	25.58	$<1x10^{-5}$ (0.000, 0.000)
E. helvum	4929	1	53	34	58	4.15	0.042 (0.047, 0.084)
P. vampyrus	4349	2	55	31	46	6.70	0.010 (0.017, 0.050)
R. ferrumequinum	4905	2	55	29	60	8.05	0.005 (0.010, 0.030)
M. lyra	4786	5	77	31	53	19.59	$1x10^{-5}$ (0.000, 0.000)
M. lucifugus	3444	4	151	17	28	106.88	$<1x10^{-5}$ (0.000, 0.000)
P. parnellii	4840	3	86	35	52	21.50	$<1x10^{-5}$ (0.000, 0.000)
IRX1/2/4							

Panda	14224	38	370	279	366	12.76	$4x10^{-3}$ (0.001, 0.002)
Dog	14247	39	333	281	386	4.40	0.036 (0.045, 0.108)
Cow	13088	40	446	237	323	63.95	$<1x10^{-5}$ (0.000, 0.000)
Dolphin	13824	50	417	267	357	32.89	$<1x10^{-5}$ (0.000, 0.000)
E. helvum	10323	26	410	155	167	115.09	$<1x10^{-5}$ (0.000, 0.000)
P. vampyrus	10668	31	417	180	194	94.09	$<1x10^{-5}$ (0.000, 0.000)
R. ferrumequinum	13923	39	395	266	338	25.18	$<1x10^{-5}$ (0.000, 0.000)
M. lyra	10939	32	421	177	238	99.56	$<1x10^{-5}$ (0.000, 0.000)
M. lucifugus	14512	48	306	291	382	0.38	0.539 (0.599, 1.000)
P. parnellii	14438	42	294	291	376	0.02	0.901 (0.901, 1.000)
IRX3/5/6							
Panda	22780	28	357	325	327	1.50	0.220 (0.314, 0.880)
Dog	22938	24	263	345	314	11.06	$9x10^{-4}$ (0.002, 0.005)
Cow	22574	24	337	322	308	0.34	0.559 (0.559, 0.880)
Dolphin	21852	21	355	326	290	1.23	0.266 (0.322, 0.880)
E. helvum	21805	23	548	299	316	73.20	$<1x10^{-5}$ (0.000, 0.000)
P. vampyrus	19550	22	465	284	259	43.74	$<1x10^{-5}$ (0.000, 0.000)
R. ferrumequinum	22690	18	364	336	331	1.12	0.290 (0.322, 0.880)
M. lyra	19594	24	511	245	267	93.59	$<1x10^{-5}$ (0.000, 0.000)
M. lucifugus	19975	42	1083	244	279	530.46	$<1x10^{-5}$ (0.000, 0.000)
P. parnellii	21655	36	532	310	279	58.53	$<1x10^{-5}$ (0.000, 0.000)
LHX1							
Panda	2857	0	21	15	32	1.00	0.317 (0.690, 1.000)
Dog	2861	1	18	15	31	0.27	0.602 (0.857, 1.000)
Cow	2650	1	24	15	29	2.08	0.150 (0.500, 1.000)
Dolphin	2829	2	36	14	31	9.68	0.002 (0.020, 0.020)
E. helvum	2852	0	16	14	31	0.13	0.715 (0.857, 1.000)
P. vampyrus	2858	0	10	14	31	0.67	0.414 (0.690, 1.000)
R. ferrumequinum	2672	1	14	13	29	0.04	0.847 (0.857, 1.000)
M. lyra	2743	1	23	13	27	2.78	0.096 (0.480, 0.864)
M. lucifugus	2747	0	14	10	24	0.67	0.414 (0.690, 1.000)
P. parnellii	2748	0	16	15	29	0.03	0.857 (0.857, 1.000)
LMO1							
Panda	3806	0	35	31	22	0.24	0.622 (0.892, 1.000)
Dog	3819	1	20	30	22	2.00	0.157 (0.314, 1.000)
Cow	3819	0	18	30	24	3.00	0.083 (0.314, 0.830)
Dolphin	3590	1	36	23	22	2.86	0.091 (0.314, 0.830)
E. helvum	3803	1	30	31	22	0.02	0.898 (0.898, 1.000)
P. vampyrus	3790	1	28	31	22	0.15	0.696 (0.892, 1.000)
R. ferrumequinum	3801	0	33	31	23	0.06	0.803 (0.892, 1.000)
M. lyra	3018	1	36	24	17	2.40	0.121 (0.314, 0.968)
M. lucifugus	3794	1	42	30	23	2.00	0.157 (0.314, 1.000)
P. parnellii	3596	0	31	29	21	0.07	0.796 (0.892, 1.000)
LMO4							
Panda	4845	0	18	24	25	0.86	0.355 (0.592, 1.000)
Dog	4702	0	20	24	22	0.36	0.546 (0.683, 1.000)
Cow	4814	0	32	24	24	1.14	0.285 (0.570, 1.000)
Dolphin	4816	1	44	24	24	5.88	0.015 (0.150, 0.150)
E. helvum	4848	0	16	24	25	1.60	0.206 (0.570, 1.000)
P. vampyrus	4710	0	17	22	24	0.64	0.423 (0.604, 1.000)

R. ferrumequinum	4834	0	28	27	22	0.02	0.892 (0.892, 1.000)
M. lyra	4164	2	35	23	19	2.48	0.115 (0.570, 1.000)
M. lucifugus	4791	0	32	24	25	1.14	0.285 (0.570, 1.000)
P. parnellii	4830	1	27	25	23	0.08	0.782 (0.869, 1.000)
MAF							
Panda	4553	1	31	36	62	0.37	0.541 (0.601, 1.000)
Dog	4480	1	33	33	61	0.00	1.000 (1.000, 1.000)
Cow	4483	5	79	36	55	16.08	$6x10^{-5}$ (0.000, 0.001)
Dolphin	4116	3	75	27	60	22.59	$<1x10^{-5}$ (0.000, 0.000)
E. helvum	4336	2	40	33	61	0.67	0.413 (0.516, 1.000)
P. vampyrus	4056	2	43	31	56	1.95	0.163 (0.233, 0.652)
R. ferrumequinum	4401	3	51	31	60	4.88	0.027 (0.054, 0.162)
M. lyra	4095	3	56	28	51	9.33	0.002 (0.007, 0.016)
M. lucifugus	4363	2	45	30	63	3.00	0.083 (0.138, 0.415)
P. parnellii	4106	2	53	31	59	5.76	0.016 (0.040, 0.112)
MEIS1							
Panda	5394	1	29	33	52	0.26	0.611 (0.764, 1.000)
Dog	4369	1	36	31	41	0.37	0.541 (0.764, 1.000)
Cow	5047	5	38	32	37	0.51	0.473 (0.764, 1.000)
Dolphin	4624	3	62	28	40	12.84	$3x10^{-4}$ (0.003, 0.003)
E. helvum	5369	1	33	38	42	0.35	0.553 (0.764, 1.000)
P. vampyrus	4847	1	35	34	41	0.01	0.904 (0.907, 1.000)
R. ferrumequinum	5337	2	37	36	45	0.01	0.907 (0.907, 1.000)
M. lyra	5230	3	64	33	40	9.91	0.002 (0.010, 0.018)
M. lucifugus	5336	3	59	34	46	6.72	0.010 (0.033, 0.080)
P. parnellii	5292	3	57	37	44	4.26	0.039 (0.098, 0.273)
MEIS2							
Panda	16830	3	113	112	139	0.00	0.947 (0.948, 1.000)
Dog	16766	1	114	115	134	0.00	0.948 (0.948, 1.000)
Cow	16831	2	126	115	133	0.50	0.479 (0.684, 1.000)
Dolphin	16103	4	156	111	131	7.58	0.006 (0.015, 0.042)
E. helvum	16572	3	118	116	133	0.02	0.896 (0.948, 1.000)
P. vampyrus	15672	2	133	109	127	2.38	0.123 (0.246, 0.738)
R. ferrumequinum	16882	2	129	112	143	1.20	0.273 (0.455, 1.000)
M. lyra	16133	5	191	107	127	23.68	$<1x10^{-5}$ (0.000, 0.000)
M. lucifugus	16763	5	221	112	135	35.68	$<1x10^{-5}$ (0.000, 0.000)
P. parnellii	16662	1	170	107	141	14.33	$2x10^{-4}$ (0.001, 0.001)
NR2F1							
Panda	15960	15	152	139	182	0.58	0.446 (0.496, 0.892)
Dog	15869	15	130	131	186	0.00	0.951 (0.951, 0.951)
Cow	15047	14	172	120	183	9.26	0.002 (0.007, 0.016)
Dolphin	15083	10	170	131	174	5.05	0.025 (0.042, 0.125)
E. helvum	15923	11	196	143	179	8.29	0.004 (0.010, 0.028)
P. vampyrus	15985	8	178	142	190	4.05	0.044 (0.055, 0.176)
R. ferrumequinum	15985	8	178	142	190	4.05	0.044 (0.055, 0.176)
M. lyra	15728	10	211	128	185	20.32	$1x10^{-5}$ (0.000, 0.000)
M. lucifugus	15839	10	186	142	181	5.90	0.015 (0.030, 0.090)
P. parnellii	15787	13	211	136	187	16.21	$6x10^{-5}$ (0.000, 0.001)
NR2F2							
Panda	12870	20	181	129	178	8.72	0.003 (0.003, 0.006)

Dog	12710	14	176	136	170	5.13	0.024 (0.024, 0.024)
Cow	12739	16	227	118	180	34.44	$<1x10^{-5}$ (0.000, 0.000)
Dolphin	11937	13	240	127	165	34.79	$<1x10^{-5}$ (0.000, 0.000)
E. helvum	11822	21	405	127	156	145.27	$<1x10^{-5}$ (0.000, 0.000)
P. vampyrus	10752	14	385	108	145	155.64	$<1x10^{-5}$ (0.000, 0.000)
R. ferrumequinum	12070	21	357	118	164	120.25	$<1x10^{-5}$ (0.000, 0.000)
M. lyra	10728	18	442	103	141	210.86	$<1x10^{-5}$ (0.000, 0.000)
M. lucifugus	10297	25	695	102	129	441.22	$<1x10^{-5}$ (0.000, 0.000)
P. parnellii	11603	19	456	124	154	190.04	$<1x10^{-5}$ (0.000, 0.000)
PAX2							
Panda	8459	2	21	29	43	1.28	0.258 (0.323, 0.945)
Dog	7705	1	49	27	44	6.37	0.012 (0.040, 0.096)
Cow	8271	2	24	35	36	2.05	0.152 (0.304, 0.912)
Dolphin	7220	2	86	22	34	37.93	$<1x10^{-5}$ (0.000, 0.000)
E. helvum	7954	2	34	24	37	1.72	0.189 (0.315, 0.945)
P. vampyrus	7659	1	39	32	40	0.69	0.406 (0.451, 0.945)
R. ferrumequinum	8299	5	33	19	34	3.77	0.052 (0.130, 0.364)
M. lyra	6728	1	36	17	32	6.81	0.009 (0.040, 0.081)
M. lucifugus	8005	3	38	32	37	0.51	0.473 (0.473, 0.945)
P. parnellii	7244	2	26	18	37	1.45	0.228 (0.323, 0.945)
PAX3							
Panda	957	0	8	14	9	1.64	0.201 (0.759, 1.000)
Dog	956	0	6	14	9	3.20	0.074 (0.740, 0.740)
Cow	957	1	9	14	8	1.09	0.297 (0.759, 1.000)
Dolphin	822	0	8	11	10	0.47	0.491 (0.759, 1.000)
E. helvum	954	0	12	13	10	0.04	0.841 (0.841, 1.000)
P. vampyrus	766	0	10	8	6	0.22	0.637 (0.759, 1.000)
R. ferrumequinum	950	0	10	15	8	1.00	0.317 (0.759, 1.000)
M. lyra	945	0	19	15	8	0.47	0.493 (0.759, 1.000)
M. lucifugus	955	0	11	13	10	0.17	0.683 (0.759, 1.000)
P. parnellii	947	0	10	13	10	0.39	0.532 (0.759, 1.000)
PAX5							
Panda	291	0	0	1	2	1.00	0.317 (0.528, 1.000)
Dog	291	0	0	2	1	2.00	0.157 (0.528, 1.000)
Cow	291	0	0	2	1	2.00	0.157 (0.528, 1.000)
Dolphin	289	0	2	2	1	0.00	1.000 (1.000, 1.000)
E. helvum	289	0	2	1	2	0.33	0.564 (0.806, 1.000)
P. vampyrus	291	0	0	1	2	1.00	0.317 (0.528, 1.000)
R. ferrumequinum	291	0	0	1	2	1.00	0.317 (0.528, 1.000)
M. lyra	123	0	0	0	2	0.00	1.000 (1.000, 1.000)
M. lucifugus	291	0	0	1	2	1.00	0.317 (0.528, 1.000)
P. parnellii	261	0	1	1	2	0.00	1.000 (1.000, 1.000)
PAX7							
Panda	1620	1	17	17	11	0.00	1.000 (1.000, 1.000)
Dog	1618	2	25	16	11	1.98	0.160 (0.533, 1.000)
Cow	1406	2	21	10	7	3.90	0.048 (0.240, 0.432)
Dolphin	1505	2	18	14	11	0.50	0.480 (0.800, 1.000)
E. helvum	1559	1	18	14	13	0.50	0.480 (0.800, 1.000)
P. vampyrus	1416	1	15	14	11	0.03	0.853 (0.952, 1.000)
R. ferrumequinum	1621	1	16	15	13	0.03	0.857 (0.952, 1.000)

M. lyra	924	1	11	9	4	0.20	0.655 (0.936, 1.000)
M. lucifugus	1023	1	16	6	4	4.55	0.033 (0.240, 0.330)
P. parnellii	1477	2	17	12	10	0.86	0.353 (0.800, 1.000)
PAX8							
Panda	157	0	0	0	3	0.00	1.000 (1.000, 1.000)
Dog	157	0	0	0	3	0.00	1.000 (1.000, 1.000)
Cow	157	0	0	0	3	0.00	1.000 (1.000, 1.000)
Dolphin	157	0	0	0	3	0.00	1.000 (1.000, 1.000)
E. helvum	149	0	4	0	3	4.00	0.046 (0.415, 0.460)
P. vampyrus	152	0	1	0	3	1.00	0.317 (0.793, 1.000)
R. ferrumequinum	127	0	3	0	2	3.00	0.083 (0.415, 0.747)
M. lyra	151	0	2	0	3	2.00	0.157 (0.523, 1.000)
M. lucifugus	157	0	0	0	3	0.00	1.000 (1.000, 1.000)
P. parnellii	79	0	0	0	1	0.00	1.000 (1.000, 1.000)
POU3F3							
Panda	2140	5	23	21	20	0.09	0.763 (0.848, 1.000)
Dog	2151	3	14	20	23	1.06	0.303 (0.490, 1.000)
Cow	2104	4	40	16	25	10.29	0.001 (0.010, 0.010)
Dolphin	2126	4	28	19	22	1.72	0.189 (0.473, 1.000)
E. helvum	2113	2	16	22	18	0.95	0.330 (0.490, 1.000)
P. vampyrus	1590	0	9	11	8	0.20	0.654 (0.818, 1.000)
R. ferrumequinum	2125	2	17	23	20	0.90	0.343 (0.490, 1.000)
M. lyra	2012	3	22	22	14	0.00	1.000 (1.000, 1.000)
M. lucifugus	2102	2	33	19	20	3.77	0.052 (0.173, 0.416)
P. parnellii	2109	2	45	21	23	8.73	0.003 (0.015, 0.027)
SALL3							
Panda	7181	14	176	101	166	20.31	$1x10^{-5}$ (0.000, 0.000)
Dog	6874	11	126	89	154	6.37	0.012 (0.012, 0.012)
Cow	6866	19	168	92	153	22.22	$<1x10^{-5}$ (0.000, 0.000)
Dolphin	6960	15	227	92	175	57.13	$<1x10^{-5}$ (0.000, 0.000)
E. helvum	5664	10	213	70	101	72.26	$<1x10^{-5}$ (0.000, 0.000)
P. vampyrus	5261	11	191	74	106	51.66	$<1x10^{-5}$ (0.000, 0.000)
R. ferrumequinum	6783	17	220	88	145	56.57	$<1x10^{-5}$ (0.000, 0.000)
M. lyra	3213	5	141	36	45	62.29	$<1x10^{-5}$ (0.000, 0.000)
M. lucifugus	6885	16	232	94	148	58.42	$<1x10^{-5}$ (0.000, 0.000)
P. parnellii	4130	10	152	51	79	50.25	$<1x10^{-5}$ (0.000, 0.000)
SHH							
Panda	3085	7	65	58	85	0.40	0.528 (0.587, 1.000)
Dog	3074	8	60	58	84	0.03	0.854 (0.854, 1.000)
Cow	2924	6	90	60	76	6.00	0.014 (0.020, 0.056)
Dolphin	2789	4	79	54	71	4.70	0.030 (0.038, 0.090)
E. helvum	2766	12	117	37	61	41.56	$<1x10^{-5}$ (0.000, 0.000)
P. vampyrus	2788	9	103	41	62	26.69	$<1x10^{-5}$ (0.000, 0.000)
R. ferrumequinum	3003	13	96	53	76	12.41	$4x10^{-4}$ (0.001, 0.002)
M. lyra	2445	7	108	46	65	24.96	$<1x10^{-5}$ (0.000, 0.000)
M. lucifugus	2572	10	95	42	57	20.50	$1x10^{-5}$ (0.000, 0.000)
P. parnellii	1656	4	89	26	38	34.51	$<1x10^{-5}$ (0.000, 0.000)
SHOX							
Panda	747	3	30	56	28	7.86	0.005 (0.017, 0.040)
Dog	735	2	22	56	23	14.82	$1x10^{-4}$ (0.001, 0.001)

Cow	611	3	17	34	28	5.67	0.017 (0.043, 0.119)
Dolphin	759	4	22	53	30	12.81	$3x10^{-4}$ (0.002, 0.003)
E. helvum	550	4	32	36	28	0.24	0.628 (0.758, 1.000)
P. vampyrus	687	5	37	41	33	0.21	0.651 (0.758, 1.000)
R. ferrumequinum	667	1	32	50	29	3.95	0.047 (0.094, 0.282)
M. lyra	202	1	10	16	10	1.38	0.239 (0.398, 1.000)
M. lucifugus	88	1	3	4	10	0.14	0.705 (0.758, 1.000)
P. parnellii	369	1	20	22	19	0.10	0.758 (0.758, 1.000)
SHOX2							
Panda	5375	0	40	58	42	3.31	0.069 (0.115, 0.408)
Dog	5365	1	44	53	46	0.84	0.361 (0.401, 0.777)
Cow	5389	1	33	57	39	6.40	0.011 (0.047, 0.110)
Dolphin	5338	0	49	52	43	0.09	0.765 (0.765, 0.777)
E. helvum	5399	1	34	56	41	5.38	0.020 (0.050, 0.140)
P. vampyrus	5143	1	32	55	36	6.08	0.014 (0.047, 0.112)
R. ferrumequinum	5422	2	35	52	47	3.32	0.068 (0.115, 0.408)
M. lyra	5257	1	42	53	39	1.27	0.259 (0.324, 0.777)
M. lucifugus	5404	1	43	58	41	2.23	0.136 (0.194, 0.544)
P. parnellii	5339	1	32	56	39	6.55	0.011 (0.047, 0.110)
SOX2							
Panda	2588	0	6	8	10	0.29	0.593 (0.659, 1.000)
Dog	2594	0	8	6	12	0.29	0.593 (0.659, 1.000)
Cow	2588	0	10	7	11	0.53	0.467 (0.659, 1.000)
Dolphin	2570	1	14	6	11	3.20	0.074 (0.247, 0.592)
E. helvum	2405	0	11	6	9	1.47	0.225 (0.375, 1.000)
P. vampyrus	2358	1	13	7	10	1.80	0.180 (0.360, 1.000)
R. ferrumequinum	2407	1	7	6	7	0.08	0.782 (0.782, 1.000)
M. lyra	2409	0	15	8	7	2.13	0.144 (0.360, 1.000)
M. lucifugus	2581	0	22	9	9	5.45	0.020 (0.100, 0.180)
P. parnellii	2468	0	27	7	8	11.76	0.001 (0.010, 0.010)
SOX3							
Panda	1357	0	10	5	13	1.00	0.317 (0.352, 0.634)
Dog	1359	0	8	6	13	0.29	0.593 (0.593, 0.634)
Cow	1346	1	19	5	13	8.17	0.004 (0.007, 0.020)
Dolphin	1263	1	21	4	13	11.56	$7x10^{-4}$ (0.002, 0.006)
E. helvum	1268	0	18	6	10	6.00	0.014 (0.020, 0.056)
P. vampyrus	1235	0	22	5	12	10.70	0.001 (0.002, 0.008)
R. ferrumequinum	1346	1	20	4	14	10.67	0.001 (0.002, 0.008)
M. lyra	1290	2	28	6	11	14.24	$2x10^{-4}$ (0.002, 0.002)
M. lucifugus	1351	1	14	5	13	4.26	0.039 (0.049, 0.117)
P. parnellii	1285	1	22	5	11	10.70	0.001 (0.002, 0.008)
SOX6							
Panda	7364	5	54	47	71	0.49	0.486 (0.608, 1.000)
Dog	7356	6	53	47	71	0.36	0.549 (0.610, 1.000)
Cow	7350	2	54	53	68	0.01	0.923 (0.923, 1.000)
Dolphin	6587	4	70	40	72	8.18	0.004 (0.008, 0.024)
E. helvum	7218	4	60	47	70	1.58	0.209 (0.299, 0.836)
P. vampyrus	6559	4	70	41	61	7.58	0.006 (0.010, 0.030)
R. ferrumequinum	7173	6	110	44	66	28.29	$<1x10^{-5}$ (0.000, 0.000)
M. lyra	7035	4	89	41	61	17.72	$3x10^{-5}$ (0.000, 0.000)

M. lucifugus	7298	4	86	47	68	11.44	$7x10^{-4}$ (0.002, 0.006)
P. parnellii	7042	3	78	43	60	10.12	0.001 (0.003, 0.007)
SOX21							
Panda	4200	5	64	51	66	1.47	0.225 (0.375, 1.000)
Dog	4232	7	49	49	66	0.00	1.000 (1.000, 1.000)
Cow	4045	4	51	45	55	0.38	0.540 (0.675, 1.000)
Dolphin	4154	3	85	50	68	9.07	0.003 (0.015, 0.027)
E. helvum	4259	1	38	52	70	2.18	0.140 (0.280, 0.840)
P. vampyrus	3626	4	55	47	61	0.63	0.428 (0.611, 1.000)
R. ferrumequinum	4242	2	49	54	67	0.24	0.622 (0.691, 1.000)
M. lyra	3674	4	80	50	67	6.92	0.009 (0.023, 0.063)
M. lucifugus	4202	2	81	50	71	7.34	0.007 (0.023, 0.056)
P. parnellii	4123	5	108	56	60	16.49	$5x10^{-5}$ (0.001, 0.001)
TFAP2A							
Panda	8060	8	95	92	107	0.05	0.826 (0.832, 1.000)
Dog	7860	7	76	88	104	0.88	0.349 (0.541, 1.000)
Cow	7813	9	162	91	101	19.92	$1x10^{-5}$ (0.000, 0.000)
Dolphin	7661	4	162	89	112	21.23	$<1x10^{-5}$ (0.000, 0.000)
E. helvum	8111	5	98	101	111	0.05	0.832 (0.832, 1.000)
P. vampyrus	7425	5	87	96	106	0.44	0.506 (0.633, 1.000)
R. ferrumequinum	7977	6	99	87	117	0.77	0.379 (0.541, 1.000)
M. lyra	7301	9	227	80	87	70.39	$<1x10^{-5}$ (0.000, 0.000)
M. lucifugus	7987	8	120	93	106	3.42	0.064 (0.128, 0.384)
P. parnellii	7935	8	129	93	113	5.84	0.016 (0.040, 0.112)
TSHZ1							
Panda	7835	5	66	55	93	1.00	0.317 (0.317, 0.317)
Dog	7793	3	69	53	93	2.10	0.147 (0.163, 0.294)
Cow	7752	1	83	60	92	3.70	0.054 (0.068, 0.162)
Dolphin	7287	4	142	50	89	44.08	$<1x10^{-5}$ (0.000, 0.000)
E. helvum	6552	4	87	42	60	15.70	$7x10^{-5}$ (0.000, 0.000)
P. vampyrus	6519	6	128	40	65	46.10	$<1x10^{-5}$ (0.000, 0.000)
R. ferrumequinum	7834	4	89	54	96	8.57	0.003 (0.004, 0.012)
M. lyra	6152	5	96	42	69	21.13	$<1x10^{-5}$ (0.000, 0.000)
M. lucifugus	7676	6	119	56	94	22.68	$<1x10^{-5}$ (0.000, 0.000)
P. parnellii	6319	7	151	36	54	70.72	$<1x10^{-5}$ (0.000, 0.000)
ZFHX1B							
Panda	16014	2	83	96	105	0.94	0.331 (0.368, 0.662)
Dog	15918	4	109	89	112	2.02	0.155 (0.194, 0.465)
Cow	15427	0	119	92	112	3.45	0.063 (0.090, 0.310)
Dolphin	15228	2	121	88	109	5.21	0.022 (0.044, 0.132)
E. helvum	16034	2	125	86	119	7.21	0.007 (0.023, 0.056)
P. vampyrus	14687	1	121	85	110	6.29	0.012 (0.030, 0.084)
R. ferrumequinum	15959	3	98	90	113	0.34	0.560 (0.560, 0.662)
M. lyra	15897	1	130	87	120	8.52	0.004 (0.020, 0.036)
M. lucifugus	15872	3	146	91	110	12.76	$4x10^{-4}$ (0.004, 0.004)
P. parnellii	16020	2	118	91	116	3.49	0.062 (0.090, 0.310)
ZIC1							
Panda	5952	2	44	68	76	5.14	0.023 (0.058, 0.161)
Dog	5958	2	39	67	76	7.40	0.007 (0.023, 0.056)
Cow	5382	4	75	62	67	1.23	0.267 (0.381, 1.000)

Dolphin	5788	4	93	67	69	4.22	0.040 (0.080, 0.240)
E. helvum	5867	2	95	73	68	2.88	0.090 (0.150, 0.450)
P. vampyrus	5756	2	80	68	73	0.97	0.324 (0.398, 1.000)
R. ferrumequinum	5878	4	67	67	72	0.00	1.000 (1.000, 1.000)
M. lyra	4236	10	200	48	44	93.16	$<1x10^{-5}$ (0.000, 0.000)
M. lucifugus	5482	5	182	62	65	59.02	$<1x10^{-5}$ (0.000, 0.000)
P. parnellii	5820	5	77	66	73	0.85	0.358 (0.398, 1.000)
ZIC2							
Panda	5486	5	81	61	90	2.82	0.093 (0.186, 0.558)
Dog	5495	6	67	59	92	0.51	0.476 (0.595, 1.000)
Cow	5365	5	115	65	83	13.89	$2x10^{-4}$ (0.002, 0.002)
Dolphin	5256	2	87	63	80	3.84	0.050 (0.125, 0.350)
E. helvum	5563	6	59	62	96	0.07	0.785 (0.785, 1.000)
P. vampyrus	5162	3	37	51	80	2.23	0.136 (0.227, 0.680)
R. ferrumequinum	5456	7	61	56	95	0.21	0.644 (0.716, 1.000)
M. lyra	5367	1	74	64	97	0.72	0.395 (0.564, 1.000)
M. lucifugus	5514	4	94	62	97	6.56	0.010 (0.033, 0.080)
P. parnellii	5410	4	111	63	94	13.24	$3x10^{-4}$ (0.002, 0.003)

Table S4

Name and functional information for the genes identified as being associated with ear development from literature surveys. The associated CNEs of which were available from the CONDOR database. Gene names, with common synonyms, type of protein and broad region of expression are given, together with the mutant phenotype – typically in the mouse *Mus musculus* – unless otherwise stated. Abbreviations: TF – transcription factor; OHC – outer hair cells;

Gene:	Type of gene/protein	Expressed in:	Mutant phenotype / putative role in auditory system	Ref:
ATBF1	Zinc finger homeobox	Developing otocyst	Direct target of Dlx5 in developing inner ear	[8]
BARHL2	Homeobox	All sensory HCs during development	Age-related progressive OHC/IHC degeneration; maintenance of cochlear HCs	[9]
BCL11A	Zinc finger TF	Ear pinna and inner ear	Possible role in regeneration of avian hair cells	[10, 11]
BCL11B	Zinc finger TF	Ear pinna and inner ear, OHC	Required for maintenance of OHCs and normal hearing	[10, 12]
BHLHB5	Basic loop helix TF	Epithelial layer of the cochlea	Unknown	[13, 14]
DACH1	Dachshund	Overlaps *Pax2* and *Eya1* in ear	No obvious ear defects	[15]
DLX1, DLX2	Homeobox	Maxillary and mandibular component of 1st pharyingeal arch	Defective malleus and incus	[16]
EBF1	TF	Developing otocyst	Interacts with Dlx5 in the developing inner ear	[8]
EMX2	Homeobox	Otic vesicle, branchial arches and skeletogenic neural crest cells	Middle-ear ossicles malformed<br>Anomalous hair cell numbers in cochlear duct<br>Shortened cochlear duct.	[17, 18]
ESRRB	Estrogen-related receptor	Cochlea, developing inner ear	Humans – DFNB35	[19]
EVI1	TF	Middle ear basal epithelial cells, fibroblasts, and neutrophil leukocytes	Inflammation of the middle ear	[20]
EYA1	Eyes absent (eya) family	Craniofacial mesenchyme	Homozygotes lack ears; Inner ear neurogenesis	[21, 22]
FIGN	AAA protein	Expressed widely	Reduced or absent semicircular canals	[23]
FOXP1	FOX family TF	Utricle and ampullae of semicircular canals	Unknown	[11, 24]
GBX2	Homeobox	Inner-ear	Absence of endolymphatic duct	[25]

			Swelling of membranous labyrinth<br>Absence of semicircular canals	
GLI3	C2H2-type zinc finger	Medial-ventral and lateral of otocyst, and surrounding mesenchyme	Vestibular defects<br>Loss of lateral semicircular canal<br>Truncation or absence of anterior semicircular canal<br>Narrower posterior semicircular canal diameter<br>Smaller inner ears with widened endolymphatic ducts	[26]
IRX1, 2 and *3* (*IRX1/2/4* and *IRX3/5/6*)	Iroquois homeobox	Complementary expression in inner ear, external ears	Speculatively:<br>*Irx1* – semicircular canal formation<br>*Irx2* – cochlea development<br>*Irx3* – cochlea development, formation of VIII ganglion.	[27]
LHX1	LIM domain TF	Saccule	Possibly neuronal differentiation	[8, 24]
LMO1	LIM domain TF	Vestibular and cochlear HCs, vestibular ganglia	Inner ear morphogenesis	[28]
LMO4	LIM domain TF	Semicircular canals, macula, crista, and spiral ganglia	Inner ear morphogenesis	[28]
MAF	TF	Otic placodes	Unknown	[29]
MEIS1, MEIS2	TALE homeobox TF	Meis1 – lateral wall of growing cochlear duct<br>Meis2 – cells of developing acoustic-vestibular ganglion	Assigning regional identity in morphogenesis, patterning, and specification of developing inner ear	[30]
Nkx-5.1 (HMX2)<br>*Nkx-5.2 (HMX3)*	Homeobox	Early – dorso-lateral otic vesicle face<br>Later – entire dorsal otic vesicle	Hmx2 – lack semicircular ducts, loss of cristae and macula utriculus, fused utriculosaccular chamber<br>Hmx3 – gross structure not severely affected, loss of sensory cells<br>Hmx2:Hmx3 – significant anatomical and neurosensory defects in vestibular system	[31-33]
NR2F1 (COUP-TFI)	TF	Early in developing otic vesicle; and maturing HCs and pillar cells	Differentiation of hair cells and support cells in organ of Corti<br>KO – displaced HCs, shortened cochlear duct	[34, 35]
NR2F2 (COUP-TFII)	TF	Early in developing otic vesicle	Patterning and differentiation of cochlear structures	[34]

PAX2	Paired-box TF	Medial wall of otic vesicle; endolymphatic duct and sac; cochlea	Rudimentary cochlea but severely malformed<br>Needed for organ of Corti formation	[36, 37]
PAX3	Paired-box TF	Neural tube, developing brain, neural crest and derivatives	Humans – Type 1 and 3 Waardenburg syndrome	[38, 39]
PAX5	Paired-box TF	Overlaps *PAX2*	No obvious inner ear defects<br>Pax5 minigene can substituted for Pax2	[37, 40]
PAX7	Paired-box TF	Various	Pax7 descendants contribute to ear tissues	[41]
PAX8	Paired-box TF	Early in developing otic placode	No detectable morphological defect in null mice	[37]
POU3F3	POU class homeobox	Supporting cells and mesenchymal cells of cochlea	No detectable embryonic morphological defects<br>Possible roles in maintenance or functional development of postnatal cochlea	[42]
SALL3	TF	Otic vesicles	Deletion may lead to deafness in humans	[43]
SHH	Hedgehog	Strongly expressed in ventral midline structures, floor plate, and notochord, but not otocyst.	Deformed inner-ear and surrounding capsule<br>Regulation of chondrogenesis	[26, 44]
SHOX	Homeobox	1st and 2nd pharyingeal arches	Possibly associated with hearing defects seen in Turner syndrome	[45]
SHOX2	Homeobox	1st and 2nd pharyingeal arches	Unknown	[45]
SOX2	Sox family	Neural tube and otocyst	Absence (Lcc/Lcc) deaf, hair cells do not differentiate and severe inner ear malformation. All 3 ampullae missing and rudimentary development of semicircular canals showed. Cochlea undercoiled, extremely small saccule and utricle.<br>Reduced expression (Ysb/Ysb) severely hearing impaired, sensory epithelium abnormal development, disorganized and fewer hair cells. 2 ampullae missing and truncated semicircular canals. Cochlea slightly undercoiled.<br>Ysb/Lcc inner-ears displayed intermediate phenotype	[46]
SOX3	Sox family	Expressed with SOX2 in neurogenic domain of otic cup	Possibly involved in the generation of neurons (chick)	[47]

SOX6	Sox family	Otic vesicle	Co-expressed with *Fgf-3*	[48]
SOX21	Sox family	Sensory epithelium in early otocyst stage, later restricted to supporting cells	Mild hearing impairment<br>Malformed hair cells	[49]
TFAP2A	TF	Neural crest cell lineages	Mutation associated with sensorineural deafness and inner ear malformation	[50]
TSHZ1	Putative zinc fingers TF	Branchial arches	Malformations of the middle ear components, including the malleus and tympanic ring	[51]
ZFHX1B	Zinc finger TF	Regenerating cochlea	Unknown	[11]
ZIC family (1, 2, 4, 5) ZIC1, ZIC2	Zinc-finger TF	Chick developing inner-ear, hindbrain, neural crest, and periotic mesenchyme	Unknown	[52]

Table S5
Gene abbreviations and EntrezGene identification numbers for the ear development and 'hearing/deafness' genes with putative associated CNEs examined by this study: Genes are broadly defined by three categories: NHSL – non-syndromic hearing loss; SHL – syndromic hearing loss; DEV – involved in auditory system development. Abbreviations: NA – not available. Cetacean and bat numbers correspond to the genes placement in Figure 5.

* Also see: Curtain MM; Donahue LR. 2007. A possible new mutation to Mmp14 MGI Direct Data Submission

Gene name:	EntrezGene ID:	Category:	Cetacean	Bat	Source:
ADCY1	107	NSHL	1	NA	[53]
AGC1	176	DEV	2	1	[54]
ALCAM	214	DEV	3	2	[54]
ATBF1 (ZFHX3)	463	DEV	4	3	[8]
ATOH1 (HATH1)	474	DEV	5	4	[55]
BARHL2	343472	DEV	6	5	[9]
BCL11A	53335	DEV	7	6	[10]
BDNF	627	DEV	8	7	[56]
BHLHB5 (BHLHE22)	27319	DEV	9	8	[13]
BMP4	652	DEV	10	9	[57]
CDH11	1009	DEV	11	10	[54]
CDH23	64072	NSHL/SHL	12	11	[53]
CHD7	55636	SHL	13	12	[53]
CLIC5	53405	NSHL	14	13	[53]
COCH	1690	NSHL	15	14	[53]
COL11A1	1301	SHL	16	15	[53]
COL12A1	1303	DEV	17	16	[54]
COL2A1	1280	DEV	18	17	[54]
COL4A3	1285	SHL	NA	18	[53]
COL4A4	1286	SHL	19	19	[53]
COL9A1	1297	SHL	20	20	[53]
DACH1	1602	DEV	21	21	[15]
DFNB31 (WHRN)	25861	NSHL/SHL	22	22	[53]
DIAPH1 (HDIA1)	1729	NSHL	23	23	[53]
DIO2	1734	DEV	24	24	[58]
DKFZp686I0554 (RGS7BP)	401190	Unclear	25	25	[59]
DLX1	1745	DEV	26	26	[16]
DLX2	1746	DEV	27	27	[16]
DSPP	1834	NSHL	NA	28	[60]
EBF (EBF1)	1879	DEV	28	29	[8]
EDN1	1906	DEV	29	30	[56]
EDNRA	1909	DEV	30	31	[56]
EDNRB	1910	SHL	31	32	[53]
EIF3S3 (EIF3H)	8667	DEV	32	33	[54]
EMX2	2018	DEV	33	34	[17]
ENTPD1 (Cd39)	953	DEV	34	35	[61]
EPS8	2059	NSHL	35	36	[53]
EVI1 (MECOM)	2122	DEV	36	37	[20]
EYA1	2138	NSHL	37	38	[53]
EYA4	2070	NSHL	38	39	[53]

FGFR1	2260	DEV	39	40	[62, 63]
FIGN	55137	DEV	40	41	[23]
FLNB	2317	DEV	41	42	[54]
FOXP1	27086	DEV	42	43	[11]
GAS1	2619	DEV	43	44	[64]
GLI3	2737	DEV	44	45	[26]
HGF	3082	NSHL	45	46	[53]
HOXA2	3199	DEV	46	47	[56]
HOXB1	3211	DEV	47	48	[56]
IRX3	79191	DEV	48	49	[27]
KCNE4 (ISK)	23704	SHL	49	50	[53]
KCNQ4	9132	NSHL	50	51	[53]
KCTD12 (KIAA1778, C13orf2, PFET1)	115207	Unclear	51	52	[65]
KIT (CKIT)	3815	DEV	52	53	[56]
KITLG (MGF)	4254	DEV	53	54	[56]
LARS2	23395	SHL	54	55	[53]
LHX1	3975	DEV	55	56	[24]
LMO1	4004	DEV	56	57	[28]
LMO4	8543	DEV	57	58	[28]
LOC253827 (MSRB3)	253827	NSHL	58	59	[53]
LOC340784 (HMX3)	340784	DEV	59	60	[31]
LOC389207 (GRXCR1)	389207	NSHL	60	61	[53]
MAF	4094	DEV	61	62	[29]
MAFB	9935	DEV	62	63	[56]
MASS1 (SLC4A11)	83959	SHL	NA	64	[66]
MEIS1	4211	DEV	64	65	[30]
MEIS2	4212	DEV	65	66	[30]
MITF	4286	SHL	66	67	[53]
*MMP14**	4323	Unclear	67	NA	[6]
MYH14	79784	NSHL	68	NA	[53]
MYO1C	4641	NSHL	69	68	[67]
MYO3A	53904	NSHL	70	69	[53]
MYO6	4646	NSHL	71	70	[53]
NEUROG1	4762	DEV	72	71	[56]
NOX3	50508	DEV	73	72	[68]
NR2F1	7025	DEV	74	73	[34]
NR2F2	7026	DEV	75	NA	[34]
NR3C2 (MLR)	4306	DEV	76	74	[69]
NTRK2 (TRKB)	4915	DEV	77	75	[56]
OPA1	4976	SHL	78	76	[70]
OTOF	9381	NSHL	79	NA	[53]
OTOG	340990	NSHL	80	NA	[53]
OTOR	56914	NSHL	81	77	[53]
OTX1	5013	DEV	82	78	[56]
OTX2	5015	DEV	83	79	[56]
PAX2	5076	DEV	84	80	[37]
PAX3	5077	SHL	85	81	[53]
PAX5	5079	DEV	86	82	[37]
PAX8	7849	DEV	87	83	[37]
PCDH15	65217	NSHL/SHL	88	84	[53]

PCTK2 (CDK17)	5128	DEV	89	85	[54]
PDE4B	5142	DEV	90	86	[54]
POU3F3	5455	DEV	91	NA	[42]
POU4F3	5456	NSHL	92	87	[53]
PRES (SLC26A5)	375611	NSHL	93	88	[53]
PRRX1 (PRX1)	5396	DEV	94	89	[56]
RARA	5914	DEV	95	90	[56]
RARG	5916	DEV	96	91	[56]
RDX	5962	NSHL	97	92	[53]
SALL1	6299	SHL	98	93	[71]
SEMA3E	9723	SHL	99	94	[53]
SHOX2	6474	DEV	100	95	[45]
SIX1	6495	NSHL	101	NA	[53]
SIX5	147912	SHL	NA	96	[53]
SLC26A4	5172	NSHL/SHL	102	97	[53]
SOX2	6657	DEV	103	98	[46]
SOX6	55553	DEV	104	99	[48]
SPRY2	10253	DEV	105	100	[72]
TBX1	6899	DEV	106	101	[73]
TCOF1	6949	SHL	107	102	[53]
TECTA	7007	NSHL	108	103	[53]
TGFB2	7042	DEV	109	104	[56]
TJP2	9414	NSHL	110	105	[53]
TMC1	117531	NSHL	111	106	[53]
TRPA1	8989	DEV	112	107	[74]
TWIST1	7291	DEV	113	108	[75]
USH2A	7399	SHL	114	109	[53]
USH3A (CLRN1, USH3)	7401	SHL	115	110	[53]
ZFHX1B (ZEB2)	9839	DEV	116	111	[11]
ZIC1	7545	DEV	117	112	[52]
ZIC2	7546	DEV	118	113	[52]

Table S5

(a) Sequence information for the four primer pairs used to amplify specific CNEs located in the *Hmx2/3* gene region. Primers were based on alignments of CNEs downloaded from the CONDOR database, and the flanking genomic regions from *Myotis lucifugus*, *Equus caballus*, *Felis catus*, *Canis familiaris*, *Bos taurus*, *Homo sapiens*, *Oryctolagus cuniculus* and *Mus musculus* downloaded from GenBank, primers were constructed under the default settings of 'Primer3', and adjusted using ambiguity bases where necessary.

Primer:	Amplifies:	Primer sequence:
Hmx2_9707_F	CRCNE00009707	TGGCAGTTGCTAACCATTACA
Hmx2_9707_R	CRCNE00009707	GATTCYGGTCATGAGTGARG
Hmx2_9711_F	CRCNE00009711	TGACGAATCTTAAAACGGATTG
Hmx2_9711_R	CRCNE00009711	GCCTGGAAAATGGAGGAGAT
Hmx2_9741_F	CRCNE00009741	CCANGAGCAGTTGGAAASTT
Hmx2_9741_R	CRCNE00009741	TAAGTGTRCYTTCRACACRTTG
Hmx2_9716_F	CRCNE00009716	TRCAMGCATCAGATTTTCAT
Hmx2_9716_R	CRCNE00009716	CAAATGACATCTCRCAATRG

(b) Final species coverage for the four amplified CNEs located in the *Hmx2/3* gene region. Ensembl – data from genomic data downloaded from Ensembl [1]; genome – data from published genomes [3]; – – data missing; GenBank accession numbers are given for newly generated sequences >200bp; NA – sequence not submitted to GenBank, but has been deposited in the Dryad Repository: http://dx.doi.org/10.5061/dryad.50kd5

Family	Species	9707	9711	9716	9741
Ornithorhynchidae	*Ornithorhynchus anatinus*	Ensembl	Ensembl	–	Ensembl
Phascolarctidae	*Phascolarctos cinereus*	–	KM981808	–	KP017254
Didelphidae	*Monodelphis domestica*	Ensembl	Ensembl	–	Ensembl
Macropodidae	*Macropus eugenii*	–	Ensembl	–	Ensembl
Dasypodidae	*Dasypus novemcinctus*	Ensembl	Ensembl	–	Ensembl
Megalonychidae	*Choloepus hoffmanni*	Ensembl	Ensembl	–	Ensembl
Elephantidae	*Loxodonta africana*	Ensembl	Ensembl	Ensembl	Ensembl
Tenrecidae	*Echinops telfairi*	Ensembl	Ensembl	–	–
Procaviidae	*Procavia capensis*	Ensembl	–	–	Ensembl
Erinaceidae	*Erinaceus europaeus*	Ensembl	–	–	Ensembl
Soricidae	*Sorex araneus*	–	–	–	Ensembl
Pteropodidae	*Macroglossus minimus*	NA	KM981782	–	KM981848
	Cynopterus brachyotis	–	KM981785	KM981816	KM981840
	Cynopterus sphinx	–	–	–	KM981841
	Dobsonia viridis	–	KM981788	KM981815	KM981842
	Eidolon helvum	genome	genome	genome	genome
	Pteropus rodricensis	–	KM981784	KM981814	KM981863
	Pteropus sp.	NA	–	–	KM981862
	Pteropus vampyrus	Ensembl	Ensembl	–	Ensembl
	Rousettus leschenaulti	–	–	–	KM981857
Rhinolophidae	*Rhinolophus borneensis*	–	–	KM981809	KM981859

	Rhinolophus creaghi	KM981771	KM981779	–	–
	Rhinolophus ferrumequinum	genome	genome	genome	genome
Hipposideridae	*Hipposideros cervinus*	KM981772	KM981780	KM981810	KM981845
	Hipposideros ridleyi	NA	KM981781	KM981811	KM981844
Megadermatidae	*Megaderma lyra*	–	–	–	KM981850
	Megaderma spasma	NA	KM981783	KM981813	KM981849
Rhinopomatidae	*Rhinopoma hardwickii*	–	–	KM981812	KM981858
Mormoopidae	*Pteronotus parnellii*	genome	genome	–	genome
Phyllostomidae	*Carollia perspicillata*	KM981773	KM981786	KM981823	KM981838
Vespertilionidae	*Kerivoula hardwickii*	KM981776	KM981789	KM981817	–
	Kerivoula minuta	–	KM981791	–	–
	Kerivoula pellucida	NA	KM981790	KM981818	–
	Plecotus auritus	–	–	KM981819	–
	Myotis lucifugus	Ensembl	Ensembl	Ensembl	Ensembl
	Murina cyclotis	KM981774	KM981792	KM981822	KM981854
	Murina rozendaali	–	KM981793	KM981820	KM981853
	Murina suilla	KM981775	KM981794	KM981821	–
Nycteridae	*Nycteris tragata*	–	KM981787	KM981824	KM981855
Camelidae	*Camelus bactrianus*	–	KM981796	KM981825	KM981837
	Vicugna pacos	–	Ensembl	–	Ensembl
Bovidae	*Bos taurus*	Ensembl	Ensembl	Ensembl	Ensembl
	Tragelaphus eurycerus	KM981778	KM981802	KM981827	KM981860
	Eudorcas thomsonii	–	KM981801	KM981828	KM981843
	Ovis aries	Ensembl	–	–	–
Suidae	*Sus scrofa*	Ensembl	Ensembl	Ensembl	Ensembl
Balaenopteridae	*Megaptera novaeangliae*	–	KM981798	KM981834	KM981851
Ziphiidae	*Hyperoodon ampullatus*	NA	KM981800	–	–
	Ziphius cavirostris	KM981777	KM981797	KM981833	KM981864
Delphinidae	*Tursiops truncatus*	Ensembl	Ensembl	Ensembl	Ensembl
	Orcinus orca	NA	KM981799	KM981835	KM981856
Ursidae	*Ailuropoda melanoleuca*	Ensembl	Ensembl	–	Ensembl
Felidae	*Felis catus*	Ensembl	Ensembl	–	Ensembl
Herpestidae	*Suricata suricatta*	–	KM981804	–	KM981861
Hyaenidae	*Hyaena brunnea*	–	KM981803	KM981830	KM981846
Canidae	*Canis familiaris*	Ensembl	Ensembl	–	Ensembl
Mustelidae	*Meles meles*	–	KM981805	KM981831	KM981852
Equidae	*Equus caballus*	Ensembl	Ensembl	–	Ensembl
Tupaiidae	*Tupaia belangeri*	Ensembl	Ensembl	Ensembl	Ensembl
Hominidae	*Homo sapiens*	Ensembl	Ensembl	Ensembl	Ensembl
	Gorilla gorilla	Ensembl	Ensembl	–	Ensembl
	Pan troglodytes	Ensembl	Ensembl	–	Ensembl
	Pongo abelii	Ensembl	Ensembl	–	Ensembl
Hylobatidae	*Nomascus leucogenys*	Ensembl	Ensembl	–	Ensembl
Cercopithecidae	*Macaca mulatta*	Ensembl	Ensembl	–	Ensembl
	Papio hamadryas	Ensembl	Ensembl	Ensembl	Ensembl
Cebidae	*Callithrix jacchus*	Ensembl	Ensembl	Ensembl	Ensembl
Galagidae	*Otolemur garnettii*	Ensembl	Ensembl	–	Ensembl

Cheirogaleidae	*Microcebus murinus*	Ensembl	Ensembl	Ensembl	Ensembl
Tarsiidae	*Tarsius syrichta*	Ensembl	–	Ensembl	Ensembl
Leporidae	*Lepus europaeus*	–	KM981795	KM981829	KM981847
	Oryctolagus cuniculus	Ensembl	Ensembl	–	Ensembl
Ochotonidae	*Ochotona princeps*	Ensembl	Ensembl	Ensembl	Ensembl
Muridae	*Mus musculus*	Ensembl	Ensembl	Ensembl	Ensembl
	Rattus norvegicus	Ensembl	Ensembl	Ensembl	Ensembl
Heteromyidae	*Dipodomys ordii*	Ensembl	Ensembl	–	–
Sciuridae	*Callosciurus notatus*	NA	KM981806	KM981832	KM981836
	Spermophilus tridecemlineatus	Ensembl	Ensembl	–	–
Cricetidae	*Clethrionomys glareolus*	–	KM981807	KM981826	KM981839
Caviidae	*Cavia porcellus*	Ensembl	Ensembl	Ensembl	Ensembl

References:

1. Flicek P, Ahmed I, Amode MR, Barrell D, Beal K, Brent S, Carvalho-Silva D, Clapham P, Coates G, Fairley S, Fitzgerald S, Gil L, Garcia-Girón C, Gordon L, Hourlier T, Hunt S, Juettemann T, Kähäri A, Keenan S, Komorowska M, Kulesha E, Longden I, Maurel T, McLaren W, Muffato M, Nag R, Overduin B, Pignatelli M, Pritchard B, Pritchard E, et al: **Ensembl 2013.** *Nucleic Acids Res* 2013, **41:**D48–D55.
2. Woolfe A, Goode DK, Cooke J, Callaway H, Smith S, Snell P, McEwen GK, Elgar G: **CONDOR: a database resource of developmentally associated conserved non-coding elements.** *BMC Dev Biol* 2007, **7:**100.
3. Tsagkogeorga G, Parker J, Stupka E, Cotton JA, Rossiter SJ: **Phylogenomic analyses elucidate the evolutionary relationships of bats.** *Curr Biol* 2013, **23:**2262–2267.
4. Zhang G, Cowled C, Shi Z, Huang Z, Bishop-Lilly KA, Xiaodong F, Wynne JW, Xiong Z, Baker ML, Zhao W, Tachedjian M, Zhu Y, Zhou P, Jiang X, Ng J, Yang L, Wu L, Xiao J, Feng Y, Chen Y, Sun X, Zhang Y, Marsh GA, Crameri G, Broder CC, Frey KG, Wang L-F, Wang J: **Comparative analysis of bat genomes provides insight into the evolution of flight and immunity.** *Science* 2013, **339:**456–460.
5. Yim H-S, Cho YS, Guang X, Kang SG, Jeong J-Y, Cha S-S, Oh H-M, Lee J-H, Yang EC, Kwon KK, Kim YJ, Kim TW, Kim W, Jeon JH, Kim S-J, Choi DH, Jho S, Kim H-M, Ko J, Kim H, Shin Y-A, Jung H-J, Zheng Y, Wang Z, Chen Y, Chen M, Jiang A, Li E, Zhang S, Hou H, et al: **Minke whale genome and aquatic adaptation in cetaceans.** *Nat Genet* 2014, **46:**88–92.
6. Zhou X, Sun F, Xu S, Fan G, Zhu K, Liu X, Chen Y, Shi C, Yang Y, Huang Z, Chen J, Hou H, Guo X, Chen W, Chen Y, Wang X, Lv T, Yang D, Zhou J, Huang B, Wang Z, Zhao W, Tian R, Xiong Z, Xu J, Liang X, Chen B, Liu W, Wang J, Pan S, Fang X, Li M, Wei F, Xu X, Zhou K, Wang J, Yang G: **Baiji genomes reveal low genetic variability and new insights into secondary aquatic adaptations.** *Nat Commun* 2013, **4:** 2708.
7. Kent WJ, Sugnet CW, Furey TS, Roskin KM, Pringle TH, Zahler AM, Haussler D: **The human genome browser at UCSC.** *Genome Res* 2002, **12:**996–1006.
8. Sajan SA, Rubenstein JL, Warchol ME, Lovett M: **Identification of direct downstream targets of Dlx5 during early inner ear development.** *Hum Mol Genet* 2011, **20:**1262–1273.
9. Li S, Price SM, Cahill H, Ryugo DK, Shen MM, Xiang M: **Hearing loss caused by progressive degeneration of cochlear hair cells in mice deficient for the *Barhl1* homeobox gene.** *Development* 2002, **129:**3523–3532.
10. Lee SC: **Characterisation of BCL11 functions in development using genetically modified mice.** University of Cambridge, The Wellcome Trust Sanger Institute, 2008.
11. Hawkins RD, Bashiardes S, Powder KE, Sajan SA, Bhonagiri V, Alvarado DM, Speck J, Warchol ME, Lovett M: **Large scale gene expression profiles of regenerating inner ear sensory epithelia.** *PLoS ONE* 2007, **2:**e525.
12. Okumura H, Miyasaka Y, Morita Y, Nomura T, Mishima Y, Takahashi S, Kominami R: ***Bcl11b* heterozygosity leads to age-related hearing loss and degeneration of outer hair cells of the mouse cochlea.** *Exp Anim* 2011, **60:**355–361.
13. Brunelli S, Innocenzi A, Cossu G: ***Bhlhb5* is expressed in the CNS and sensory organs during mouse embryonic development.** *Gene Expr Patterns* 2003, **3:**755–759.
14. Fritzsch B, Eberl DF, Beisel KW: **The role of bHLH genes in ear development and evolution: revisiting a 10-year-old hypothesis.** *Cell Mol Life Sci* 2010, **67:**3089–3099.
15. Heanue TA, Davis RJ, Rowitch DH, Kispert A, McMahon AP, Mardon G, Tabin CJ: ***Dach1*, a vertebrate homologue of *Drosophila* dachshund, is expressed in the**

developing eye and ear of both chick and mouse and is regulated independently of *Pax* and *Eya* genes. *Mech Dev* 2002, **111:**75–87.
16. Merlo GR, Zerega B, Paleari L, Trombino S, Mantero S, Levi G: **Multiple functions of *Dlx* genes.** *Int J Dev Biol* 2000, **44:**619–626.
17. Rhodes CR, Parkinson N, Tsai H, Brooker D, Mansell S, Spurr N, Hunter AJ, Steel KP, Brown SDM: **The homeobox gene *Emx2* underlies middle ear and inner ear defects in the deaf mouse mutant pardon.** *J Neurocytol* 2003, **32:**1143–1154.
18. Holley M, Rhodes C, Kneebone A, Herde MK, Fleming M, Steel KP: **Emx2 and early hair cell development in the mouse inner ear.** *Dev Biol* 2010, **340:**547–556.
19. Collin RWJ, Kalay E, Tariq M, Peters T, van der Zwaag B, Venselaar H, Oostrik J, Lee K, Ahmed ZM, Caylan R, Li Y, Spierenburg HA, Eyupoglu E, Heister A, Riazuddin S, Bahat E, Ansar M, Arslan S, Wollnik B, Brunner HG, Cremers CWRJ, Karaguzel A, Ahmad W, Cremers FPM, Vriend G, Friedman TB, Riazuddin S, Leal SM, Kremer H: **Mutations of *ESRRB* encoding estrogen-related receptor beta cause autosomal-recessive nonsyndromic hearing impairment DFNB35.** *Am J Hum Genet* 2008, **82:**125–138.
20. Parkinson N, Hardisty-Hughes RE, Tateossian H, Tsai H-T, Brooker D, Morse S, Lalane Z, MacKenzie F, Fray M, Glenister P, Woodward A-M, Polley S, Barbaric I, Dear N, Hough TA, Hunter AJ, Cheeseman MT, Brown SDM: **Mutation at the *Evi1* locus in Junbo mice causes susceptibility to otitis media.** *PLoS Genet* 2006, **2:**1556–1564.
21. Xu PX, Adams J, Peters H, Brown MC, Heaney S, Mass R: ***Eya1*-deficient mice lack ears and kidneys and show abnormal apoptosis of organ primordia.** *Nat Genet* 1999, **23:**113–117.
22. Ahmed M, Xu J, Xu PX: **EYA1 and SIX1 drive the neuronal developmental program in cooperation with the SWI/SNF chromatin-remodeling complex and SOX2 in the mammalian inner ear.** *Development* 2012, **139:**1965–1977.
23. Cox GA, Mahaffey CL, Nystuen A, Letts VA, Frankel WN: **The mouse fidgetin gene defines a new role for AAA family proteins in mammalian development.** *Nat Genet* 2000, **26:**198–202.
24. Sajan SA, Warchol ME, Lovett M: **Towards a systems biology of mouse inner ear organogenesis: gene expression pathways, patterns and network analysis.** *Genetics* 2007, **177:**631–653.
25. Lin Z, Cantos R, Patente M, Wu DK: ***Gbx2* is required for the morphogenesis of the mouse inner ear: a downstream candidate of hindbrain signaling.** *Development* 2005, **132:**2309–2318.
26. Bok J, Dolson DK, Hill P, Ruther U, Epstein DJ, Wu DK: **Opposing gradients of Gli repressor and activators mediate Shh signaling along the dorsoventral axis of the inner ear.** *Development* 2007, **134:**1713–1722.
27. Bosse A, Zülch A, Becker M-B, Torres M, Gómez-Skarmeta J, Modolell J, Gruss P: **Identification of the vertebrate Iroquois homeobox gene family with overlapping expression during early development of the nervous system.** *Mech Dev* 1997, **69:**169–181.
28. Deng M, Pan L, Xie X, Gan L: **Differential expression of LIM domain-only (LMO) genes in the developing mouse inner ear.** *Gene Expr Patterns* 2006 **6:**857–863.
29. Lecoin L, Sii-Felice K, Pouponnot C, Eychene A, Felder-Schmittbuhl M-P: **Comparison of maf gene expression patterns during chick embryo development.** *Gene Expr Patterns* 2004 **4** 35–46.
30. Sanchez-Guardado LO, Ferran JL, Rodriguez-Gallardo L, Puelles L, Hidalgo-Sanchez M: ***Meis* gene expression patterns in the developing chicken inner ear.** *J Comp Neurol* 2011, **519:**125–147.

31. Rinkwitz-Brandt S, Justus M, Oldenettel I, Arnold HH, Bober E: **Distinct temporal expression of mouse *Nkx-5.1* and *Nkx-5.2* homeobox genes during brain and ear development.** *Mech Dev* 1995, **52:**371–381.
32. Hadrys T, Braun T, Rinkwitz-Brandt S, Arnold HH, Bober E: ***Nkx5-1* controls semicircular canal formation in the mouse inner ear.** *Development* 1998, **125:**33–39.
33. Wang WD, Lufkin T: ***Hmx* homeobox gene function in inner ear and nervous system cell-type specification and development.** *Exp Cell Res* 2005, **306:**373–379.
34. Tang LS, Alger HM, Lin F, Pereira FA: **Dynamic expression of COUP-TFI and COUP-TFII during development and functional maturation of the mouse inner ear.** *Gene Expr Patterns* 2005, **5:**587–592.
35. Tang LS, Alger HM, Pereira FA: **COUP-TFI controls Notch regulation of hair cell and support cell differentiation.** *Development* 2006, **133:**3683–3693.
36. Burton Q, Cole LK, Mulheisen M, Chang W, Wu DK: **The role of *Pax2* in mouse inner ear development.** *Dev Biol* 2004, **272:**161–175.
37. Bouchard M, de Caprona D, Busslinger M, Xu P, Fritzsch B: **Pax2 and Pax8 cooperate in mouse inner ear morphogenesis and innervation.** *BMC Dev Biol* 2010, **10:**89.
38. Baldwin C, Hoth C, Macina R, Milunsky A: **Mutations in *PAX3* that cause Waardenburg syndrome type 1: Ten new mutations and review of the literature.** *Am J Med Genet* 2005, **58:**115–122.
39. Bondurand N, Pingault V, Goerich DE, Lemort N, Sock E, Le Caignec C, Wegner M, Goossens M: **Interaction between *SOX10*, *PAX3* and *MITF*, three genes altered in Waardenburg syndrome.** *Am J Hum Genet* 2000, **67:**1907–1917.
40. Bouchard M, Pfeffer P, Busslinger M: **Functional equivalence of the transcription factors Pax2 and Pax5 in mouse development.** *Development* 2000, **127:**3703–3713.
41. Murdoch B, DelConte C, García-Castro MI: **Pax7 lineage contributions to the mammalian neural crest.** *PLoS ONE* 2012, **7:**e41089.
42. Mutai H, Nagashima R, Sugitani Y, Noda T, Fujii M, Matsunaga T: **Expression of *Pou3f3/Brn-1* and its genomic methylation in developing auditory epithelium.** *Dev Neurobiol* 2009, **69:**913–930.
43. Parrish M, Ott T, Lance-Jones C, Schuetz G, Schwaeger-Nickolenko A, Monaghan AP: **Loss of the *Sall3* gene leads to palate deficiency, abnormalities in cranial nerves, and perinatal lethality** *Mol Cell Biol* 2004, **24:**7102–7112
44. Liu W, Li G, Chien JS, S. R, Zhang H, Chiang C, Frenz DA: **Sonic Hedgehog regulates otic capsule chondrogenesis and inner ear development in the mouse embryo.** *Dev Biol* 2002, **248:**240–250.
45. Clement-Jones M, Schiller S, Rao E, Blaschke RJ, Zuniga A, Zeller R, Robson SC, Binder G, Glass I, Strachan T, Lindsay S, Rappold GA: **The short stature homeobox gene *SHOX* is involved in skeletal abnormalities in Turner syndrome.** *Hum Mol Genet* 2000, **9:**695–702.
46. Kiernan AE, Pelling AL, Leung KKH, Tang ASP, Bell DM, Tease C, Lovell-Badge R, Steel KP, Cheah KSE: ***Sox2* is required for sensory organ development in the mammalian inner ear.** *Nature* 2005, **434:**1031–1035.
47. Neves J, Kamaid A, Alsina B, Giraldez F: **Differential expression of Sox2 and Sox3 in neuronal and sensory progenitors of the developing inner ear of the chick.** *J Comp Neurol* 2007, **503:**487–500.
48. Murakami A, Ishida S, Thurlow J, Revest J-M, Dickson C: **SOX6 binds CtBP2 to repress transcription from the *Fgf-3* promoter.** *Nucleic Acids Res* 2001, **29:**3347–3355.

49. Hosoya M, Fujioka M, Matsuda S, Ohba H, Shibata S, Nakagawa F, Watabe T, Wakabayashi K-I, Saga Y, Ogawa K, Okano HJ, Okano H: **Expression and function of Sox21 during mouse cochlea development.** *Neurochem Res* 2011, **36:**1261–1269.
50. Tekin M, Sirmaci A, Yüksel-Konuk B, Fitoz S, Sennaroğlu L: **A complex TFAP2A allele is associated with branchio-oculo-facial syndrome and inner ear malformation in a deaf child.** *Am J Med Genet A* 2009, **149A:**427–430.
51. Coré N, Caubita X, Metchata A, Bonedb A, Djabalib M, Fasanoa L: ***Tshz1* is required for axial skeleton, soft palate and middle ear development in mice** *Dev Biol* 2007, **308:**407–420.
52. Warner SJ, Hutson MR, Oh S-H, Gerlach-Bank LM, Lomax MI, Barald KF: **Expression of *ZIC* genes in the devlopment of the chick inner ear and nervous system.** *Dev Dyn* 2003, **226:**702–712.
53. **The Hereditary Hearing loss Homepage** [http://hereditaryhearingloss.org]
54. Ficker M, Powles N, Warr N, Pirvola U, Maconochie M: **Analysis of genes from inner ear developmental-stage cDNA subtraction reveals molecular regionalization of the otic capsule.** *Dev Biol* 2004, **268:**7–23.
55. Chonko KT, Jahan I, Stone J, Wright MC, Fujiyama T, Hoshino M, Fritzsch B, Maricich SM: **Atoh1 directs hair cell differentiation and survival in the late embryonic mouse inner ear.** *Dev Biol* 2013, **381:**401–410.
56. Fekete DM: **Development of the vertebrate ear: insights from knockouts and mutants.** *Trends Neurosci* 1999, **22:**263–269.
57. Blauwkamp MN, Beyer LA, Kabara L, Takemura K, Buck T, King WM, Dolan DF, Barald KF, Raphael Y, Koenig RJ: **The role of bone morphogenetic protein 4 in inner ear development and function.** *Hear Res* 2007, **225:**71–79.
58. Ng L, Goodyear RJ, Woods CA, Schneider MJ, Diamond E, Richardson GP, Kelley MW, Germain DL, Galton VA, Forrest D: **Hearing loss and retarded cochlear development in mice lacking type 2 iodothyronine deiodinase.** *Proc Natl Acad Sci U S A* 2004, **101:**3474–3479.
59. Seim I, Fang X, Xiong Z, Lobanov AV, Huang Z, Ma S, Feng Y, Turanov AA, Zhu Y, Lenz TL, Gerashchenko MV, Fan D, Yim SH, Yao X, Jordan D, Xiong Y, Ma Y, Lyapunov AN, Chen G, Kulakova OI, Sun Y, Lee S-G, Bronson RT, Moskalev AA, Sunyaev SR, Zhang G, Krogh A, Wang J, Gladyshev VN: **Genome analysis reveals insights into the evolution, physiology, and longevity of the Brandt's bat.** *Nat Commun* 2013, **4:**2212.
60. Xiao S, Yu C, Chou X, Yuan W, Wang Y, Bu L, Fu G, Qian M, Yang J, Shi Y, Hu L, Han B, Wang Z, Huang W, Liu J, Chen Z, Zhao G, Kong X: **Dentinogenesis imperfecta 1 with or without progressive hearing loss is associated with distinct mutations in DSPP.** *Nat Genet* 2001, **27:**201–204.
61. Vlajkovic SM, Housley GD, Thorne PR, Gupta R, Enjyoji K, Cowan PJ, Liberman MC, Robson SC: **Preservation of cochlear function in Cd39 deficient mice.** *Hear Res* 2009, **253:**77–82.
62. Torres M, Giráldez F: **The development of the vertebrate inner ear.** *Mech Dev* 1998, **71:**5–21.
63. Calvert JA, Dedos SG, Hawker K, Fleming M, Lewis MA, Steel KP: **A missense mutation in *Fgfr1* causes ear and skull defects in hush puppy mice.** *Mamm Genome* 2011, **22:**290–305.
64. Seppala M, Depew MJ, Martinelli DC, Fan C-M, Sharpe PT, Cobourne MT: ***Gas1* is a modifier for holoprosencephaly and genetically interacts with sonic hedgehog.** *J Clin Investig* 2007, **117:**1575–1584.
65. Sand PG, Langguth B, Itzhacki J, Bauer A, Geis S, Cárdenas-Conejo ZE, Pimentel V, Kleinjung T: **Resequencing of the auxiliary GABA(B) receptor subunit gene *KCTD12* in chronic tinnitus.** *Front Syst Neurosci* 2012, **6:**41.

66. Siddiqui S, Zenteno JC, Rice A, Chacón-Camacho O, Naylor SG, Rivera-de la Parra D, Spokes DM, James N, Toomes C, Inglehearn CF, Ali M: **Congenital hereditary endothelial dystrophy caused by *SLC4A11* mutations progresses to Harboyan syndrome.** *Cornea* 2014, **33:**247–251.
67. Zadro C, Alemanno MS, Bellacchio E, Ficarella R, Donaudy F, Melchionda S, Zelante L, Rabionet R, Hilgert N, Estivill X, Van Camp G, Gasparini P, Carella M: **Are *MYO1C* and *MYO1F* associated with hearing loss?** *Biochim Biophys Acta* 2009, **1792:**27–32.
68. Flaherty JP, Fairfield HE, Spruce CA, McCarty CM, Bergstrom DE: **Molecular characterization of an allelic series of mutations in the mouse *Nox3* gene.** *Mamm Genome* 2011, **22:**156–169.
69. Jin DX, Lin Z, Lei D, Jianxin B: **The role of glucocorticoids for spiral ganglion neuron survival.** *Brain Res* 2009, **1277:**3–11.
70. Leruez S, Milea D, Defoort-Dhellemmes S, Colin E, Crochet M, Procaccio V, Ferré M, Lamblin J, Drouin V, Vincent-Delorme C, Lenaers G, Hamel C, Blanchet C, Juul G, Larsen M, Verny C, Reynier P, Amati-Bonneau P, Bonneau D: **Sensorineural hearing loss in OPA1-linked disorders.** *Brain* 2013, **136:**e236.
71. Liang Y, Shen D, Cai W: **Two coding single nucleotide polymorphisms in the *SALL1* gene in Townes-Brocks syndrome: a case report and review of the literature.** *Journal of Pediatric Surgery* 2008, **43:**391–393.
72. Shim K, Minowada G, Coling DE, Martin GR: ***Sprouty2*, a mouse deafness gene, regulates cell fate decisions in the auditory sensory epithelium by antagonizing FGF signaling.** *Dev Cell* 2005, **8:**553–564.
73. Vitelli F, Viola A, Morishima M, Pramparo T, Baldini A, Lindsay E: ***TBX1* is required for inner ear morphogenesis.** *Hum Mol Genet* 2003, **12:**2041–2048.
74. Corey DP, García-Añoveros J, Holt JR, Kwan KY, Lin S-Y, Vollrath MA, Amalfitano A, Cheung EL-M, Derfler BH, Duggan A, Géléoc GSG, Gray PA, Hoffman MP, Rehm HL, Tamasauskas D, Zhang D-S: ***TRPA1* is a candidate for the mechanosensitive transduction channel of vertebrate hair cells.** *Nature* 2004, **432:**723–730.
75. Zhang Y, Blackwell EL, McKnight MT, Knutsen GR, Vu WT, Ruest LB: **Specific inactivation of *Twist1* in the mandibular arch neural crest cells affects the development of the ramus and reveals interactions with hand2.** *Dev Dyn* 2012, **241:**924–940.
