# Supplementary figures and images for: Divergent evolutionary rates in vertebrate and mammalian specific conserved non-coding elements (CNEs) in echolocating mammals
Source: BMC Evol Biol. 2014 Dec 19;14:261. doi: 10.1186/s12862-014-0261-5 (PMC4302572; doi:10.1186/s12862-014-0261-5)

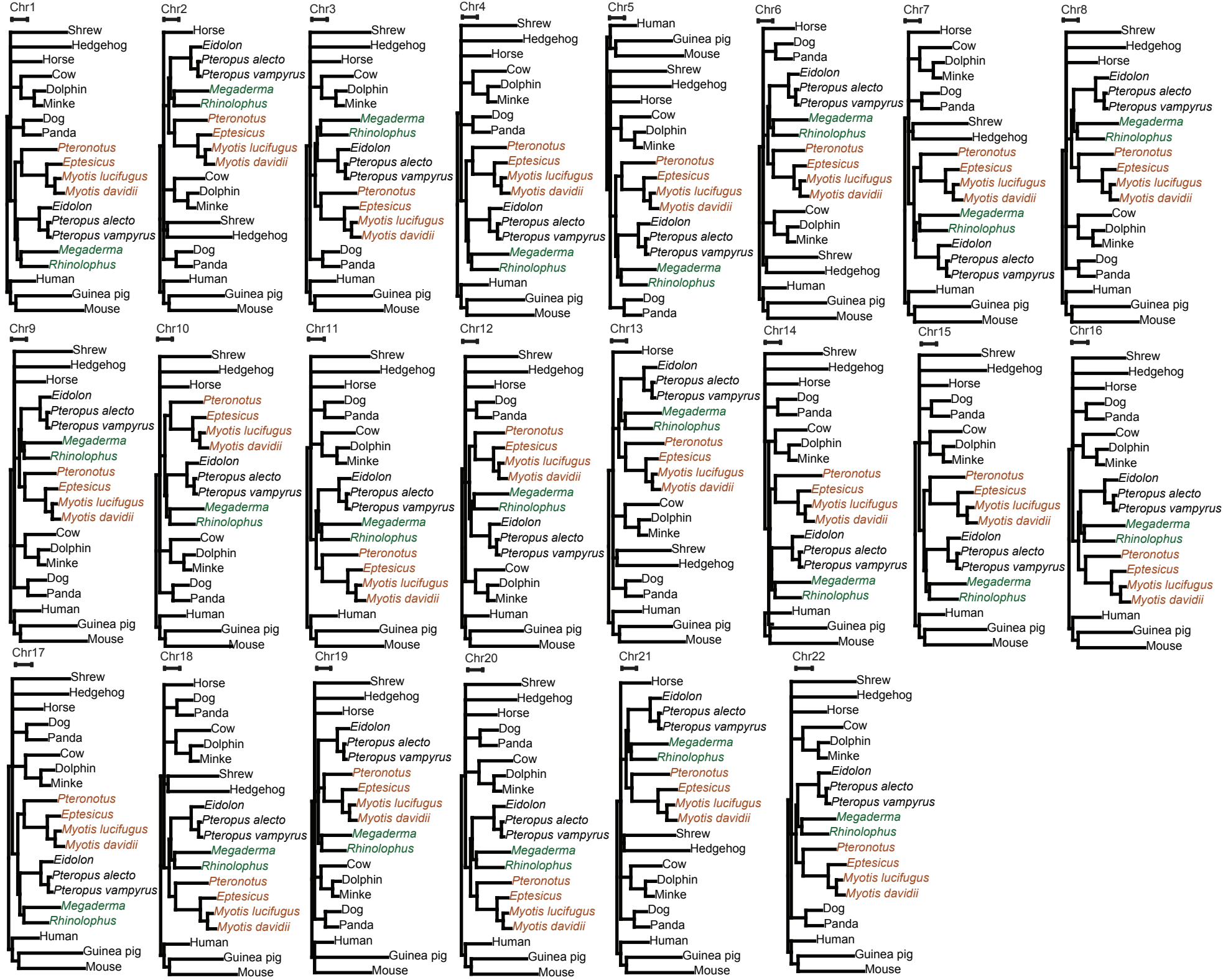

Supplement: Additional file 2: Figure S1. — Majority consensus trees calculated for sets of ML trees grouped according to their position on human chromosomes. Echolocating species were represented by the following bats: R. ferrumequinum, M. lyra, P. parnellii, M. lucifugus, M. davidii and E. fuscus and the echolocating dolphin (T. truncatus). For comparison we included the non-echolocating Old World fruit bats P. vampyrus, P. alecto and E. helvum; and the baleen minke whale (B. acutorostrata). Bats groups are designated by the following colours: echolocating Yinpterochiroptera – green; non-echolocating Yinpterochiroptera – black; Yangochiroptera – brown. A total of 6,109 alignments contained sequence information for all 20 taxa and were used to construct the ML phylogenies. Number of trees used per chromosome: Chr1: 572; Chr2: 561; Chr3: 443; Chr4: 320; Chr5: 424; Chr6: 375; Chr7: 357; Chr8: 293; Chr9: 267; Chr10: 314; Chr11: 341; Chr12: 348; Chr13: 89; Chr14: 282; Chr15: 171; Chr16: 169; Chr17: 302; Chr18: 148; Chr19: 55; Chr20: 157; Chr21: 57; Chr22: 63). [file 12862_2014_261_MOESM2_ESM.pdf]

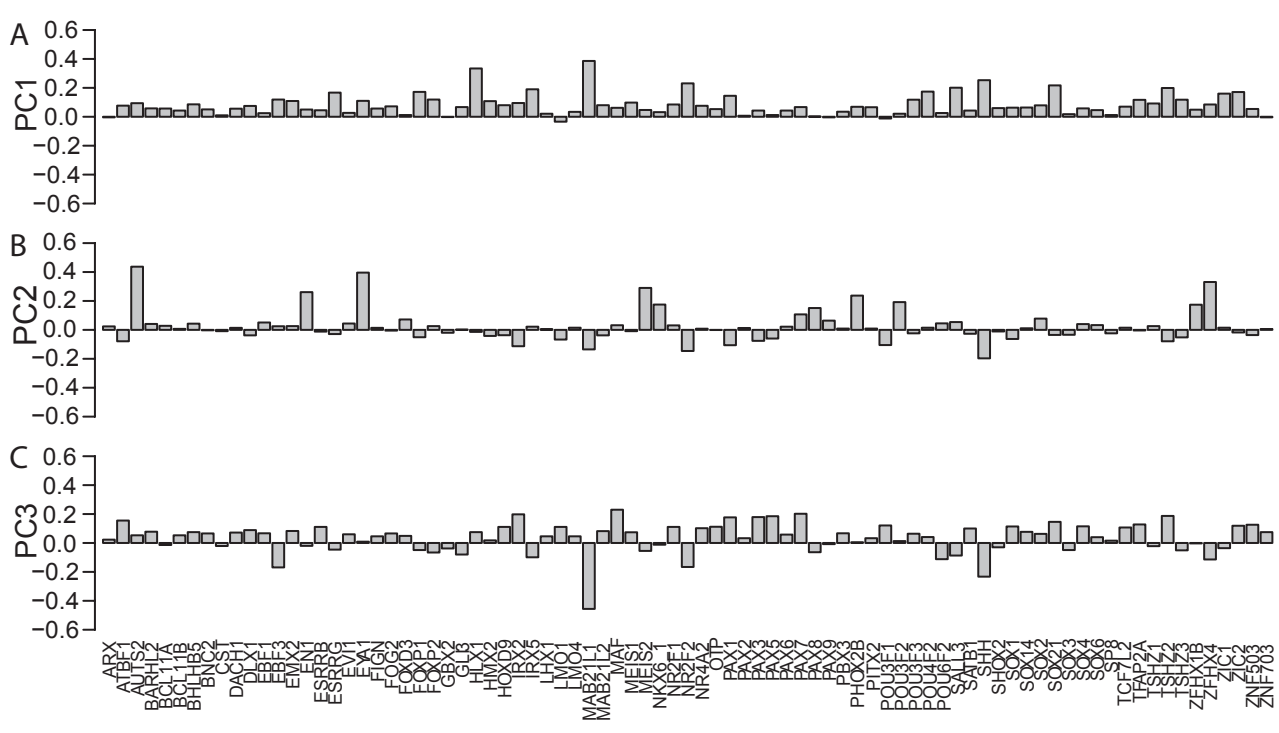

Supplement: Additional file 3: Figure S2. — PCA loadings along (A) PC–1 (B) PC–2 and (C) PC–3 for summed root to tip branch lengths of 83 concatenated CNE alignments across 26 mammalian species. [file 12862_2014_261_MOESM3_ESM.pdf]

A

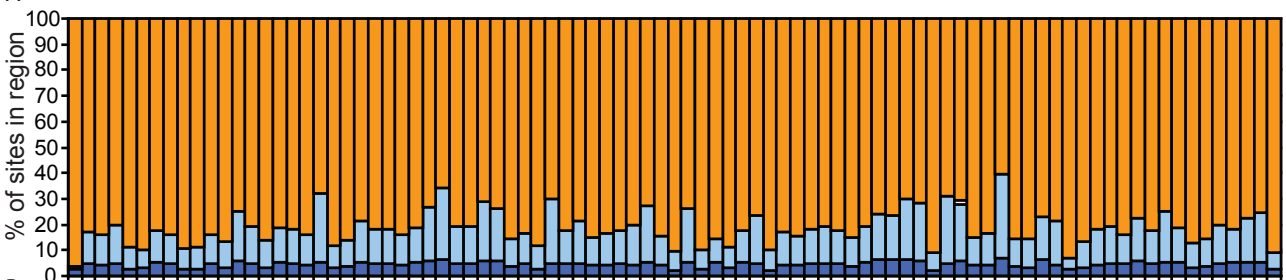

B

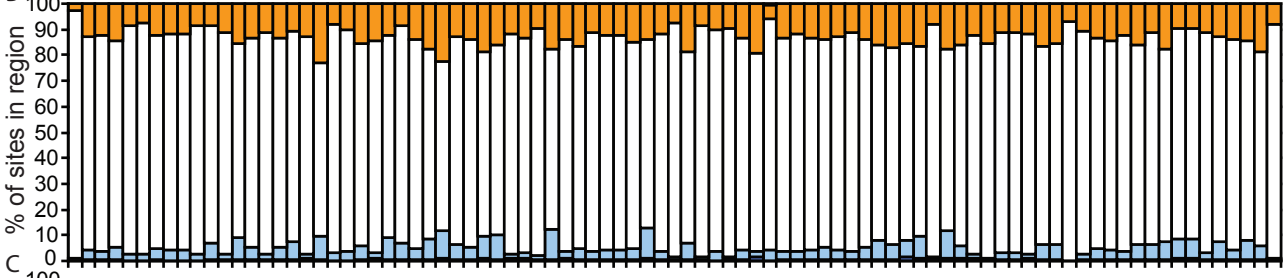

1

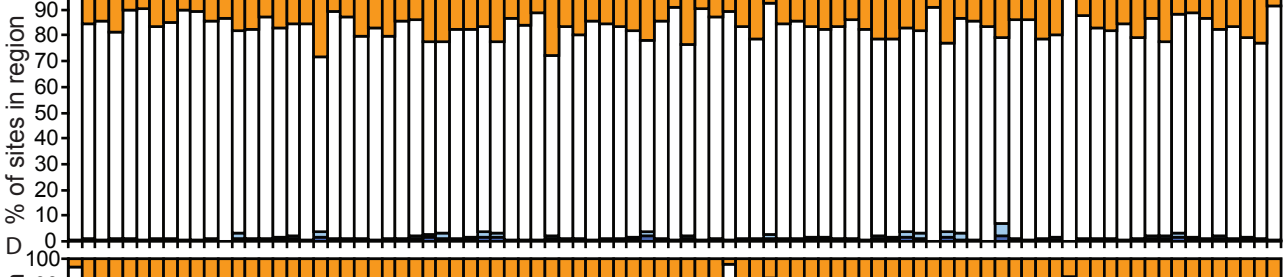

on

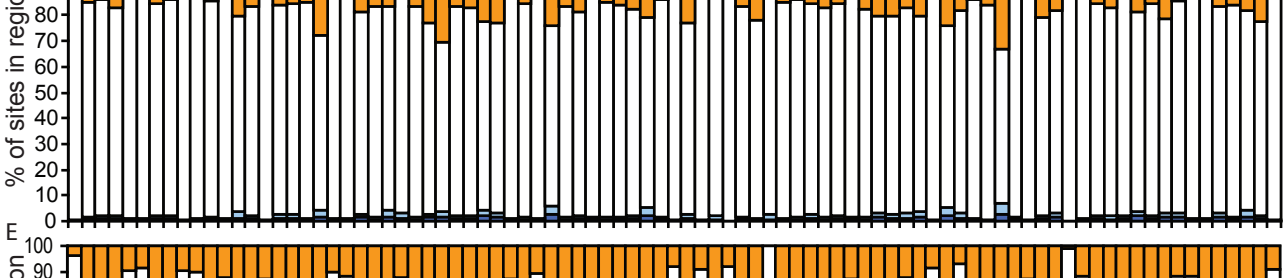

dic

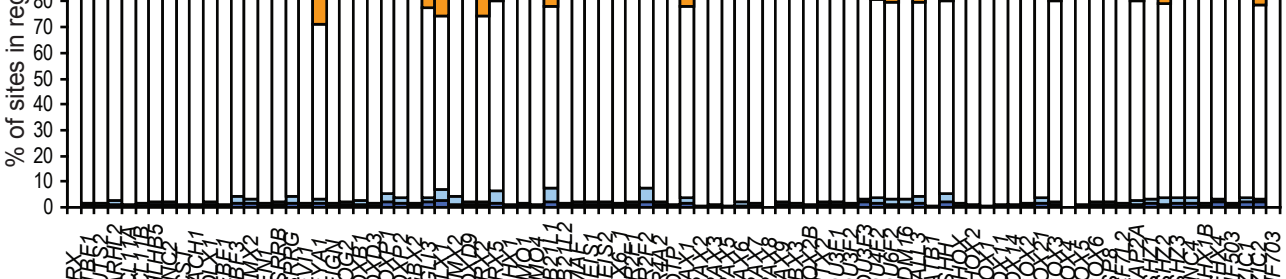

Genomic region

Supplement: Additional file 5: Figure S4. — Levels of acceleration and conservation across genomic regions as estimated by phyloP scores. (A) across all mammals included in the study; (B) all bats included in the study; (C) Old World fruit bat sub-clade; (D) echolocating Yinpterochiroptera sub-clade; (E) Yangochiroptera sub-clade. Colours indicate the percentage of sites calculated to show accelerated evolution (light blue); accelerated evolution with negative P > −0.05 (dark blue); ‘neutral’ score = 0 (white); conservation (orange); conservation with P < 0.05 (red). Genomic regions containing genes associated with auditory system development are indicated with *. [file 12862_2014_261_MOESM5_ESM.pdf]

A

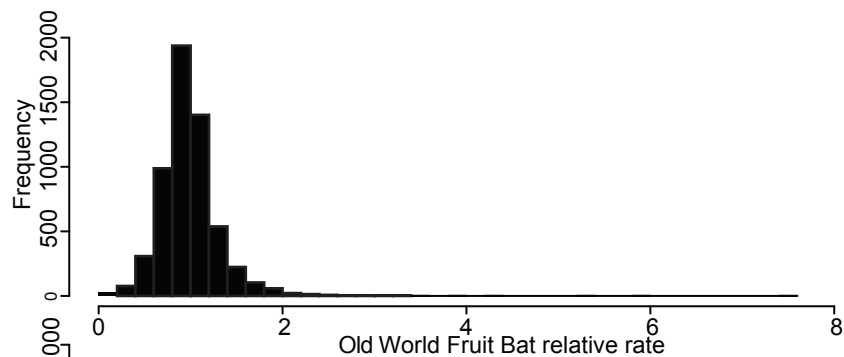

B

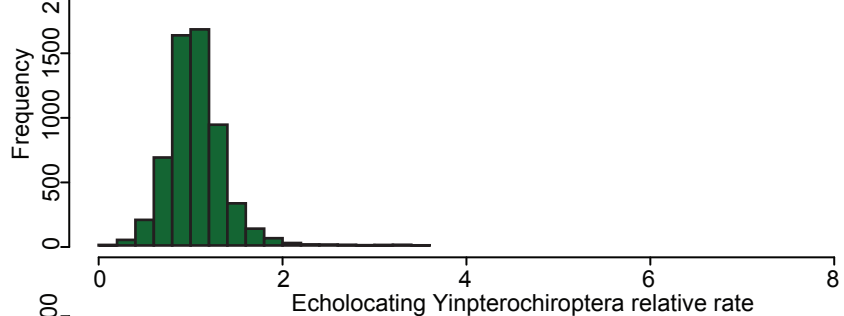

C

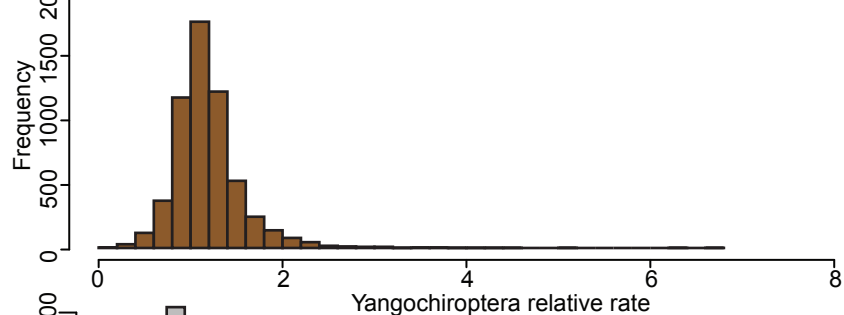

D

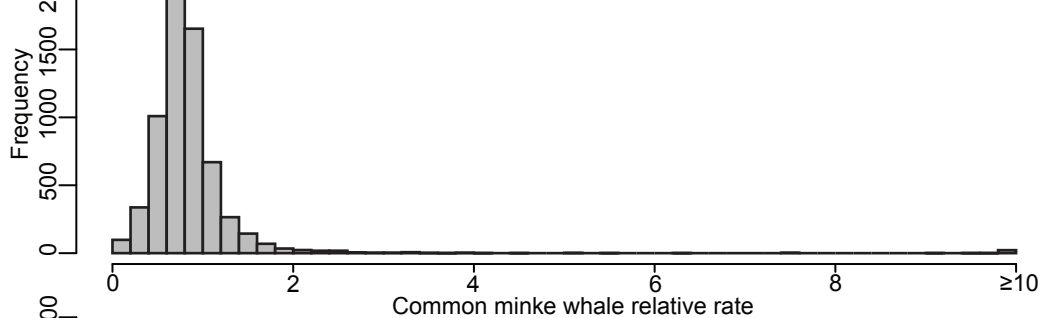

E

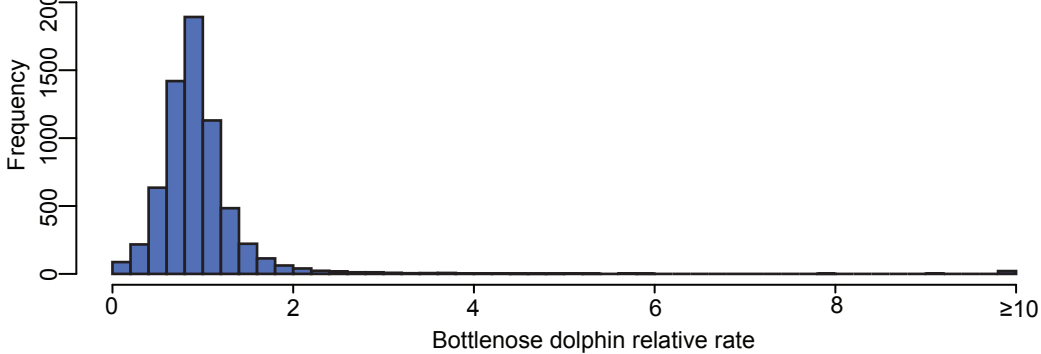

Supplement: Additional file 6: Figure S5. — Frequency histograms of estimated relative rates across mammalian specific CNEs grouped by nearest gene – in each analysis the rate for the foreground clade of interest in given relative to the background rate, which is equal to 1. Rates were calculated in BASEML using the Felsenstein-84 model of substitution, local clock and alpha and kappa estimated from the data (A) Old World fruit bats; (B) echolocating Yinpterochiroptera; (C) Yangochiroptera; (D) Common minke whale and (E) Bottlenose dolphin. [file 12862_2014_261_MOESM6_ESM.pdf]

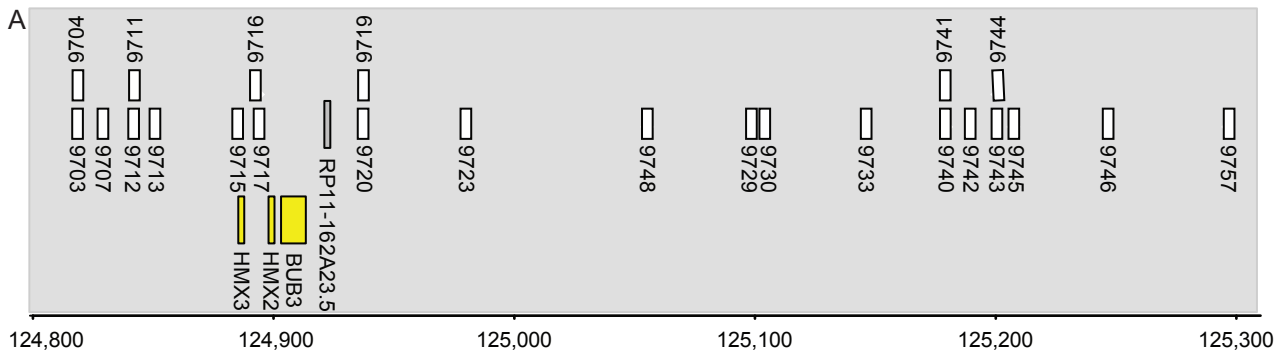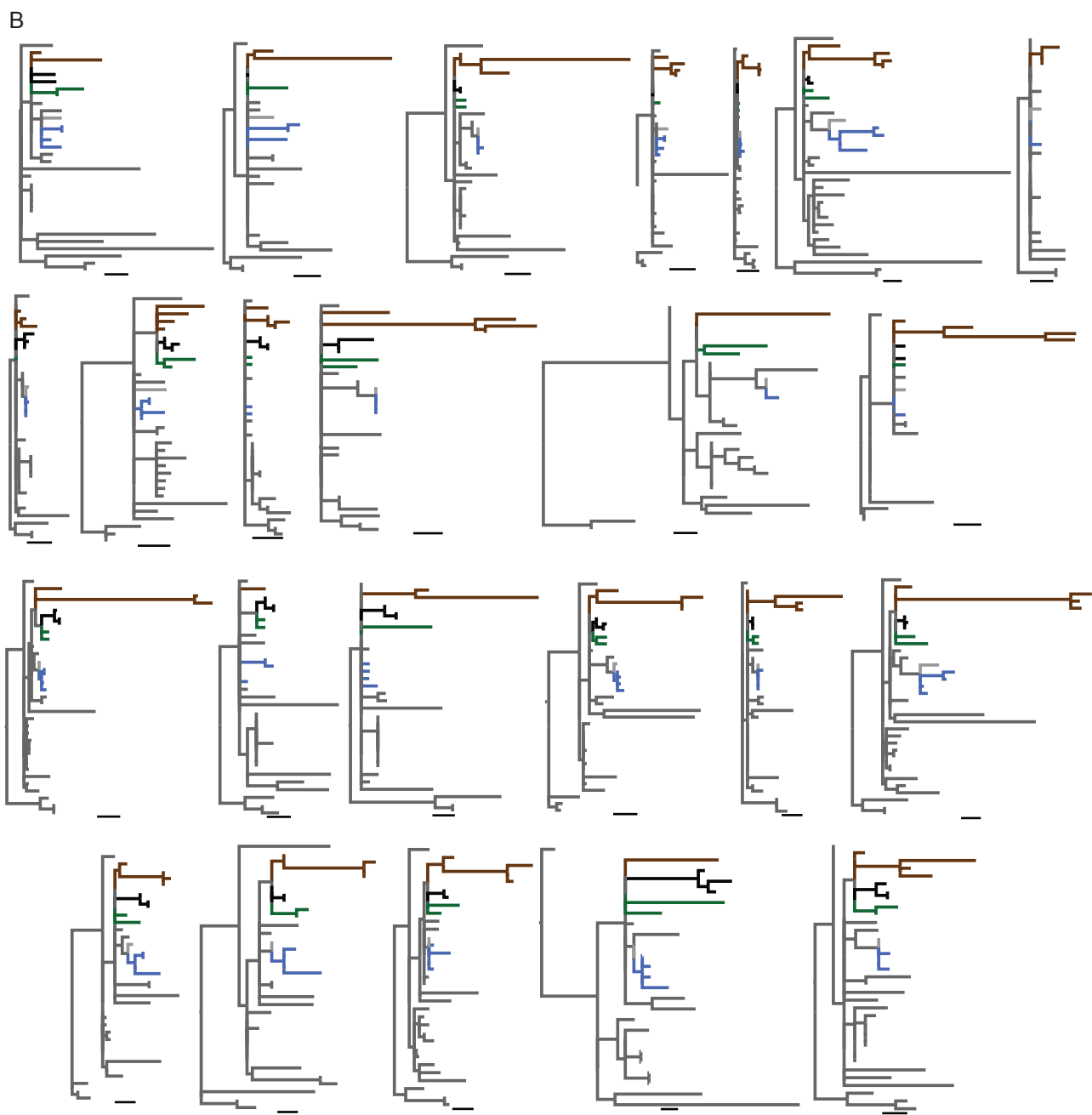

Supplement: Additional file 7: Figure S6. — Estimated lineage-specific nucleotide substitution rates across 24 CNE sequence alignments from the Hmx2/3 gene region. Rates were calculated in BASEML using the Felsenstein-84 model of substitution, no clock and alpha and kappa estimated from the data. (A) The approximate genomic location (in kilo-bases) of the 24 CNEs (white blocks) under study and the three proximate genes (yellow blocks; HMX3, HMX2 and BUB3 – left to right), also present in this region is RP11-162A23.5 (grey block) which is a pseudogene, based on the H. sapiens genome (Ensembl release 75). (B) Estimated branch lengths with fixed species topology calculated from alignments of CNEs from the Hmx2/3 genomic region, the order of trees (left to right, top to bottom) match the numbered CNEs from above (9703 – 9757). Non-focal branches (dark grey), bat branches are coloured as follows: Old World fruit bats (black); echolocating Yinpterochiroptera (green); Yangochiroptera (brown) and cetacean branches are coloured as follows: non-echolocating minke whale (light grey) and echolocating toothed whales (blue). [file 12862_2014_261_MOESM7_ESM.pdf]

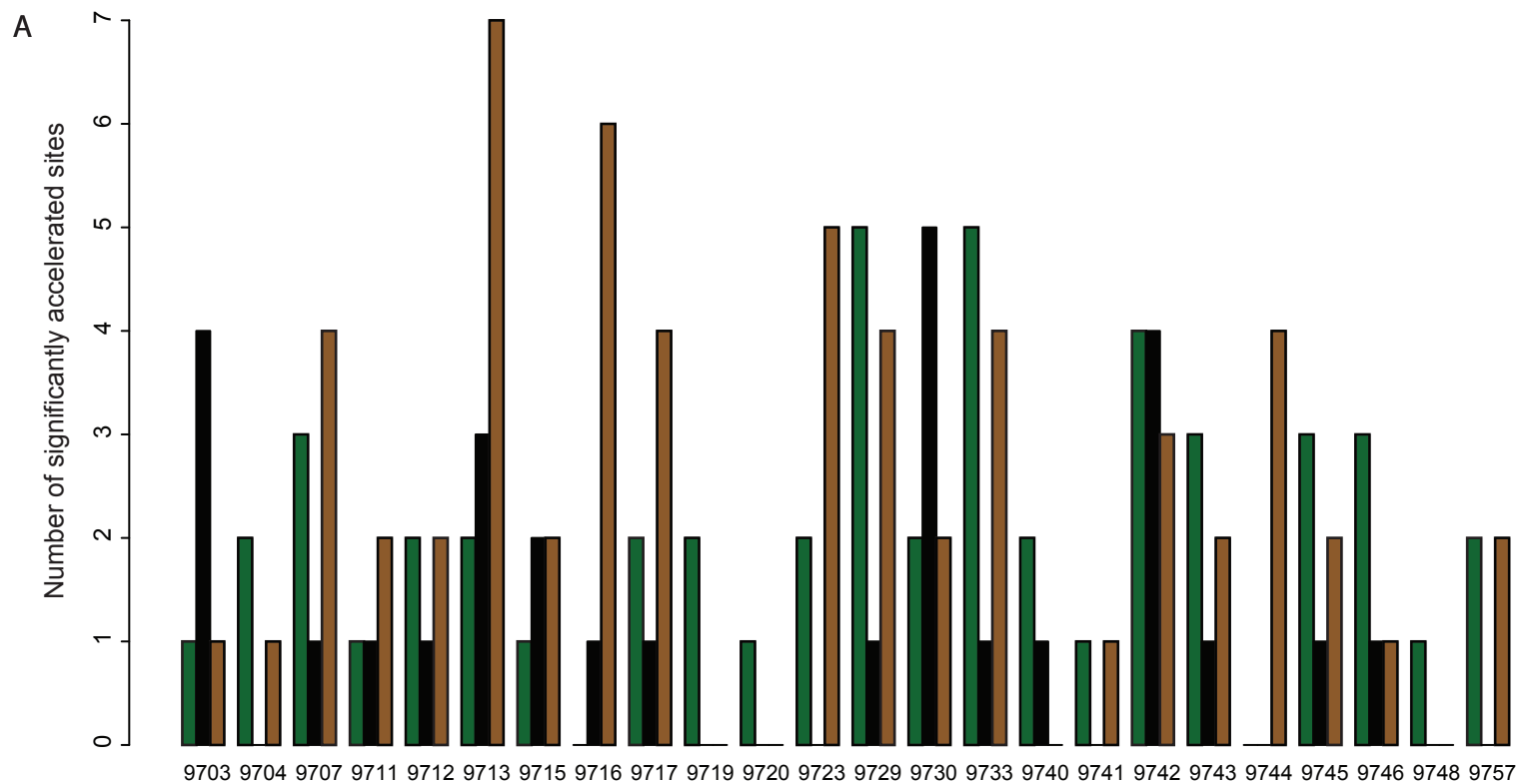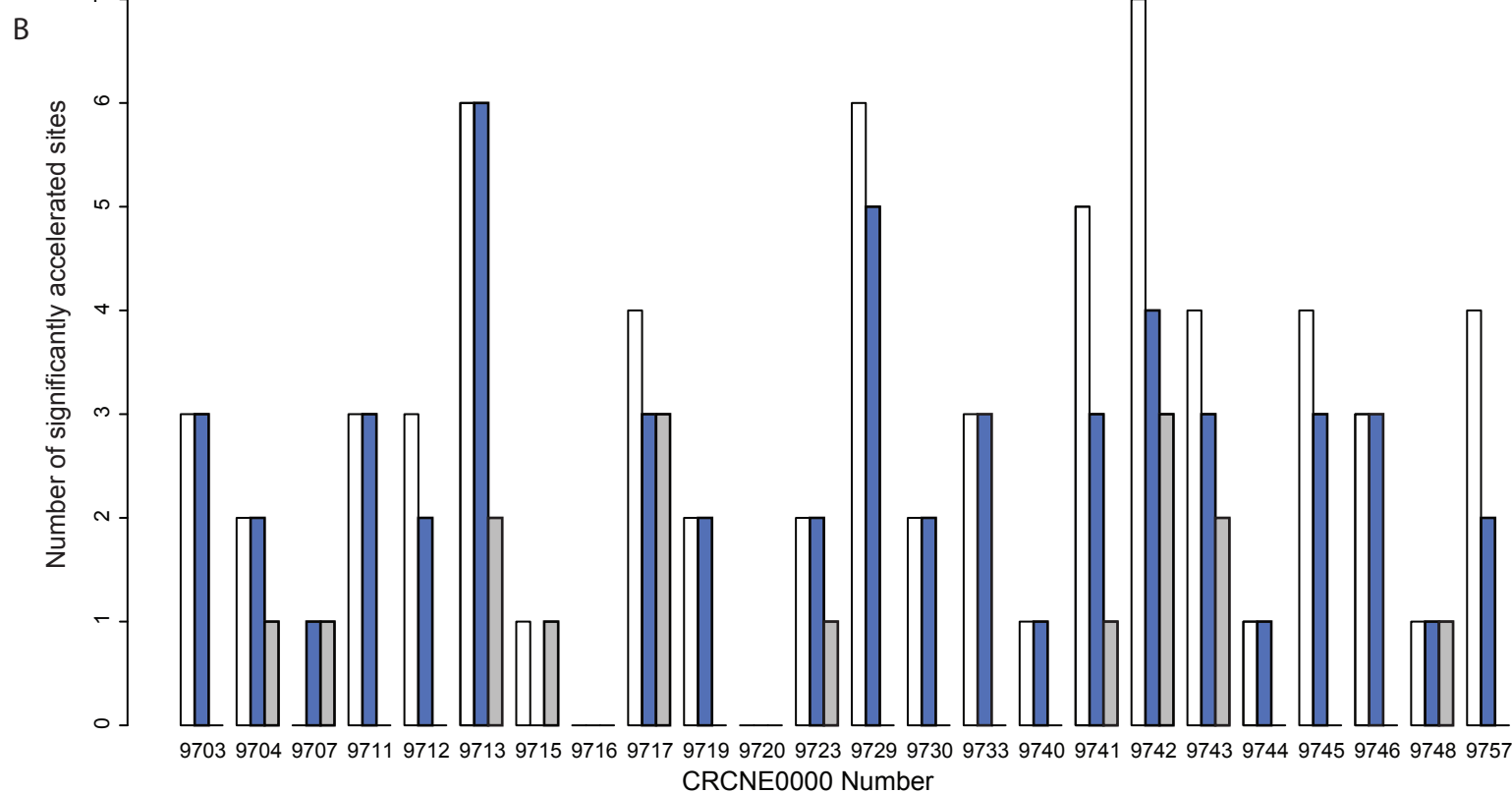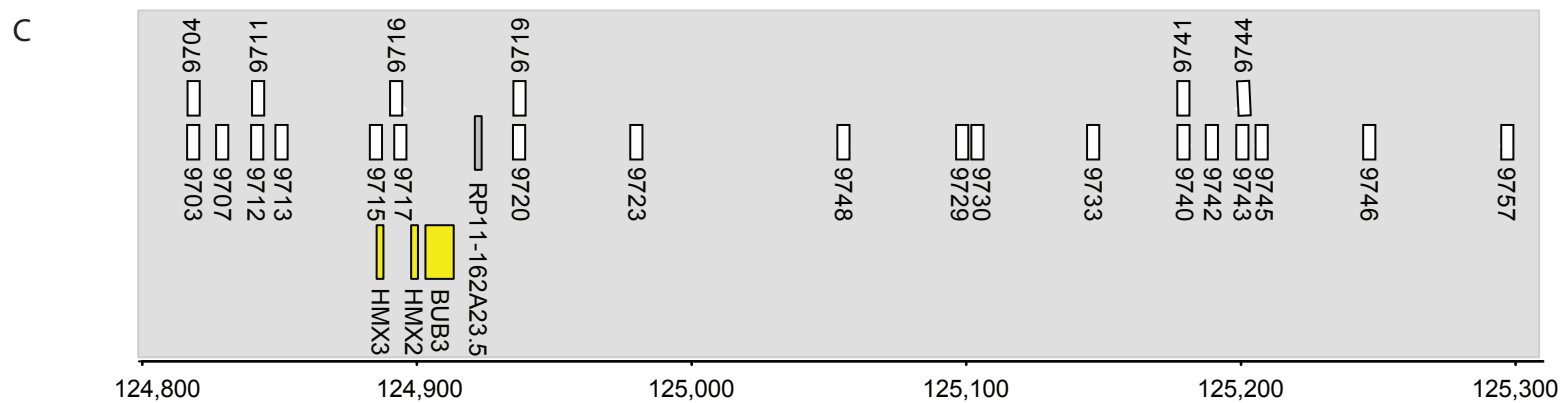

Supplement: Additional file 8: Figure S7. — Number of significantly accelerated sites for 24 CNE sequence alignments from the Hmx2/3 gene region for (A) bats and (B) cetaceans as calculated by phyloP. (A) Coloured bars represent echolocating Yinpterochiroptera (green); Old World fruit bats (black) and Yangochiroptera (brown). (B) Coloured bars represent all cetaceans (white); echolocating toothed whales (blue) and minke whale (light grey). (C) Approximate genomic location (in kilo-bases) of the 24 CNEs (white blocks) under study and the three proximate genes (yellow blocks; HMX3, HMX2 and BUB3 – left to right) – see legend of Additional file 7: Figure S6 for full details. [file 12862_2014_261_MOESM8_ESM.pdf]
