# Supplementary material for: Divergent evolutionary rates in vertebrate and mammalian specific conserved non-coding elements (CNEs) in echolocating mammals
Source: BMC Evol Biol. 2014 Dec 19;14:261. doi: 10.1186/s12862-014-0261-5 (PMC4302572; doi:10.1186/s12862-014-0261-5)
Supplement: Additional file 4: Figure S3. — Branch-specific substitution rates across CNEs from 83 genomic regions. (A) Bat branches: (i) ancestral echolocating Yinpterochiroptera branch; (ii) R. ferrumequinum; (iii) M. lyra; (iv) ancestral Old World fruit bat branch; (v) E. helvum; (vi) P. vampyrus; (vii) ancestral Yangochiroptera branch; (viii) M. lucifugus; (ix) P. parnellii; (x) ancestral common bat branch; (xi) ancestral Yinpterochiroptera branch; (xii) graphical representation of the branches under study. (B) Cetartiodactyla branches: (i) ancestral Cetartiodactyla branch (ii) B. taurus; (iii) T. truncatus. [file 12862_2014_261_MOESM4_ESM.pdf]

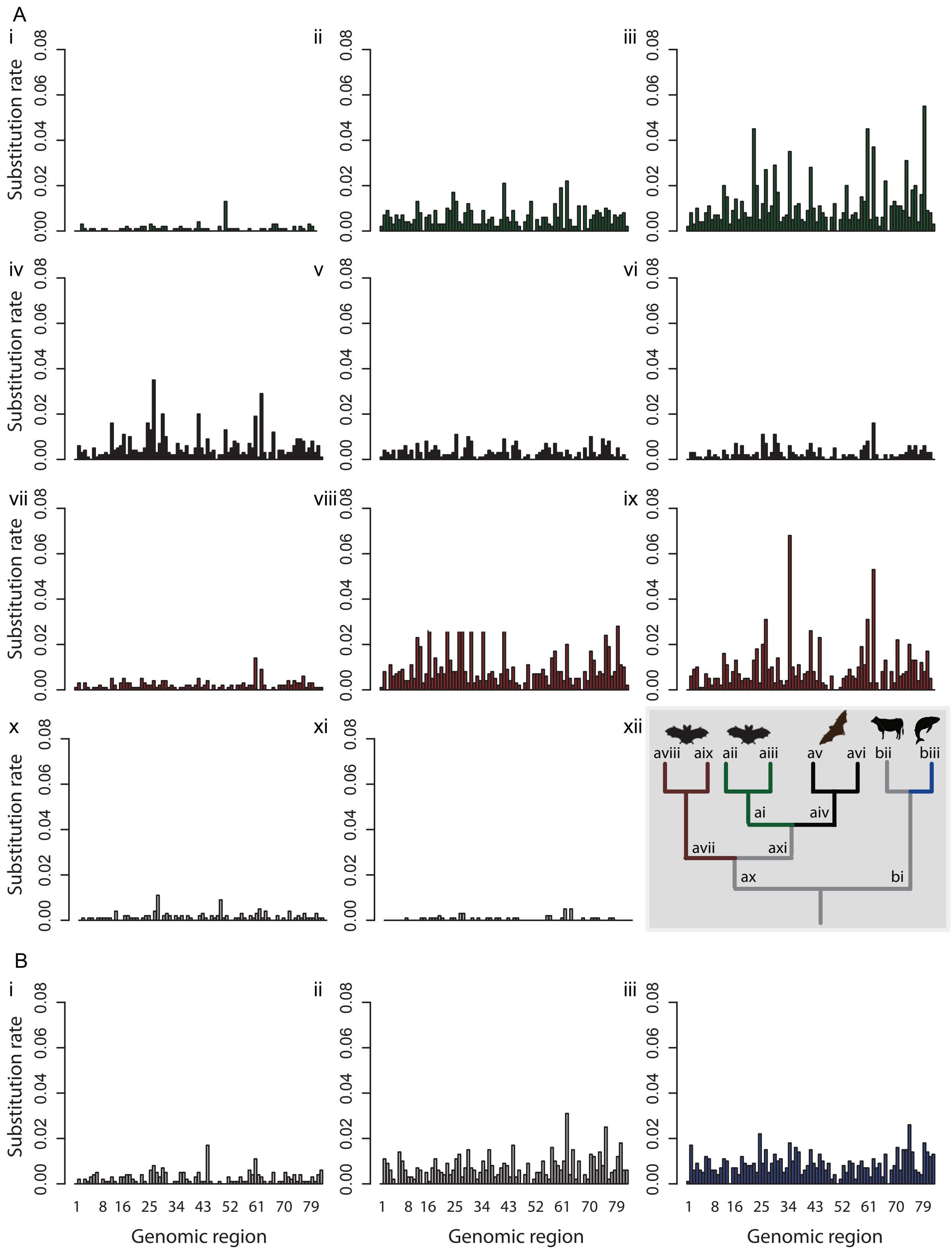

A
i
ii
iii
iv
v
vi
vii
viii
ix
x
xi
xii
Substitution rate
0.00
0.02
0.04
0.06
0.08
aviii
aix
aii
aiii
av
avi
bii
biii
ai
aiv
avii
axi
ax
bi
B
1
8
16
25
34
43
52
61
70
79
Genomic region
